# Inter-city movement pattern of notifiable infectious diseases in China: a social network analysis

**DOI:** 10.1016/j.lanwpc.2024.101261

**Published:** 2024-12-13

**Authors:** Lin-Jie Yu, Peng-Sheng Ji, Xiang Ren, Yan-He Wang, Chen-Long Lv, Meng-Jie Geng, Jin-Jin Chen, Tian Tang, Chun-Xi Shan, Sheng-Hong Lin, Qiang Xu, Guo-Lin Wang, Li-Ping Wang, Simon I. Hay, Wei Liu, Yang Yang, Li-Qun Fang

**Affiliations:** a State Key Laboratory of Pathogen and Biosecurity, Academy of Military Medical Science, Beijing, PR China; b Center for Disease Control and Prevention (Health Inspection Office) of Yuhang District, Hangzhou, Zhejiang, PR China; c Department of Statistics, Franklin College of Arts and Science, University of Georgia, GA, United States; d Division of Infectious Disease, Key Laboratory of Surveillance and Early-warning on Infectious Disease, Chinese Center for Disease Control and Prevention, Beijing, PR China; e The 968th Hospital of Joint Logistics Support Force of PLA, Jinzhou, Liaoning, PR China; f Department of Health Metrics Sciences, School of Medicine, University of Washington, Seattle, WA, United States; g Institute for Health Metrics and Evaluation, University of Washington, Seattle, WA, United States

**Keywords:** Human mobility, Network analysis, Disease migration

## Abstract

**Background:**

Co-existence of efficient transportation networks and geographic imbalance of medical resources greatly facilitated inter-city migration of patients of infectious diseases in China.

**Methods:**

To characterize the migration patterns of major notifiable infectious diseases (NIDs) during 2016–2020 in China, we collected migratory cases, who had illness onset in one city but were diagnosed and reported in another, from the National Notifiable Infectious Disease Reporting System, and conducted a nationwide network analysis of migratory cases of major NIDs at the city (prefecture) level.

**Findings:**

In total, 2,674,892 migratory cases of NIDs were reported in China during 2016–2020. The top five diseases with the most migratory cases were hepatitis B, tuberculosis, hand, foot and mouth disease (HFMD), syphilis, and influenza, accounting for 79% of all migratory cases. The top five diseases with the highest proportions of migratory cases were all zoonotic or vector-borne (37.89%‒99.98%). The network analysis on 14 major diseases identified three distinct migration patterns, where provincial capitals acted as key node cities: short distance (e.g., pertussis), long distance (e.g., HIV/AIDS), and mixed (e.g., HFMD). Strong drivers for patient migration include population mobility and labor flow intensities between cities as well as the economic development level of the destination city.

**Interpretation:**

Collaborative prevention and control strategies should target cities experiencing frequent patient migration and cater to unique migration patterns of each disease. Addressing disparity in healthcare accessibility can also help alleviate case migration and thereby reduce cross-regional transmission.

**Funding:**

10.13039/501100012166National Key Research and Development Program of China.

**Research in context**

**Evidence before this study**

We searched PubMed database on June 14th, 2024 by terms “((Communicable Diseases [MeSH Major Topic]) AND ((Human Migration [MeSH Major Topic]) OR (human mobility))) AND (China [Title/Abstract])”, without language restrictions. Among 81 articles found, 64 related articles revealed the associations between human mobility and infectious diseases, mainly focusing the sexually transmitted diseases represented by HIV/AIDS and pandemic infectious diseases such as COVID-19. Many studies identified significant associations between human mobility and the spread of infectious diseases, e.g., migrant workers and cross-border travelers served as drivers while travel restrictions served as an inhibitor for disease transmission. Most of these studies focused on individual infectious diseases. Only one study examined the effect of human mobility changes on multiple NIDs during the COVID-19 pandemic, but the study was limited to a single city. In addition, these studies focused on either long-distance (international or inter-provincial) and short-distance travels (between cities within a province or between areas within a city), but not both. There lacks a comprehensive comparison of inter-city migration patterns and network structures across different infectious diseases at the national scale and a thorough investigation on potential socioeconomic and geographical drivers for the migration of NID cases.

**Added value of this study**

In this study, we conducted a nationwide epidemiological study on the migration patterns of major NIDs during 2016–2020 in China. We characterized the similarities and differences of the number and proportion of migratory cases among NIDs and mapped city-specific inflow and outflow proportions of the NIDs. We constructed city-level networks to study the inter-city migration of 14 major NIDs and developed machine learning models to associate the flow of migratory cases with socio-economic and geographic variables for the 14 major NIDs. We found zoonotic and vector-borne diseases were associated with higher proportions of migratory cases. We identified three typical migration modes of infectious disease patients: short distance between adjacent cities represented by pertussis, brucellosis, mumps, hepatitis E, hepatitis A and Shigellosis; long distance for HIV/AIDS, syphilis, gonorrhea, and tuberculosis; and mixed for HFMD, influenza, hepatitis B and hepatitis C. We found strong connections between several developing inland cities in southwestern China and developed coastal cities in the Yangtze River Delta and the Pearl River Delta in the long-distance mode. We demonstrated that the intensity of population mobility and labor flow between cities were the most important driving factors and provincial capitals acted as key node cities.

**Implications of all the available evidence**

Migratory NID cases mainly flow from under-developed areas towards developed provincial capitals or coastal cities, suggesting geographic disparity in healthcare resources as an underlying driver for the migration. The similarity between the migration network structure of sexually transmitted diseases and the labor force flow implies the role of geographic disparity in economic development as another driver. Collaborative prevention and control strategies between cities bearing frequent patient migration and policies reducing disparity in healthcare accessibility across regions are needed.

## Introduction

A key component in preventing and controlling infectious diseases is accurate characterization of the role of human mobility in disease spread, especially in densely populated countries like China.^1^, ^2^, ^3^, ^4^, ^5^, ^6^, ^7^ Many studies have demonstrated the impact of human mobility on the spatial spread of a variety of infectious diseases, including but not limited to COVID-19, influenza, measles, hand-foot-and-mouth disease (HFMD), dengue and HIV/AIDS.^8^, ^9^, ^10^, ^11^, ^12^, ^13^ While network analysis has been widely and successfully applied to fields like child trafficking, illegal wildlife trade, and human mobility patterns, few studies have examined the role of human mobility in disease transmission from a network perspective, possibly due to the challenge of tracking the travel history of individual patients.^14^, ^15^, ^16^, ^17^, ^18^ The spectrum of diseases spreading via short- and long-distance migration remains unclear, and less is known about the relative importance between short-distance vs. long-distance travel. While long-distance traveling of patients or carriers of infectious pathogens is less frequent, it could help seed the pathogens in previously less exposed, and thus more susceptible, populations. This may help to sustain endemicity even if the pathogens are not highly transmissible locally.^19^ The relative importance of short-vs. long-distance traveling on the transmission dynamics of infectious diseases may be context-dependent, driven by demographic, environmental and socioeconomic features of the population as well as biological characteristics of the underlying pathogens.^13^^,^^20^

Since its reopening in the early 1980s, China has undergone rapid urbanization, resulting in a highly mobile population,^3^^,^^4^ facilitating inter-regional spread of infectious diseases. During its modernization, China established a real-time internet-based infectious disease surveillance system, the National Notifiable Infectious Disease Reporting System (NNIDRS),^21^ which has covered over 168,000 various medical and health institutions across China; the timely reporting rate for notifiable infectious diseases nationwide has reached over 99%, with the average time from diagnosis to reporting being approximately 4 h.^22^ The NNIDRS captures comprehensive data on notifiable infectious diseases (NID), including demographic, clinical and diagnostic information for every individual case. In particular, the availability of addresses at the times of disease onset and reporting for each case makes it possible to characterize domestic inter-regional migration patterns for a variety of infectious diseases. Here we perform a nationwide network analysis of migratory cases of major notifiable infectious disease at the city (prefecture) level in China.

## Methods

### Data collection

Data on NIDs reported from 1 January 2016 to 31 December 2020 was collected from the NNIDRS. According to *the Law on the Prevention and Control of Infectious Diseases in the People’s Republic of China*, 39 types of notifiable infectious diseases were reported in NNIDRS. We further divided three primary disease groups into finer disease types: the viral hepatitis cases were separated into hepatitis A, hepatitis B, hepatitis C, hepatitis D, and hepatitis E, the dysentery cases into shigellosis and amoebic dysentery, and the typhoid/paratyphoid cases into typhoid and paratyphoid. We excluded 2 diseases (untyped viral hepatitis; infectious diarrhea other than cholera, dysentery and typhoid) due to unclear pathogenesis. In total, we assessed 44 infectious diseases in this study. Similar to previous studies, a migratory case is defined as a patient whose residential city on the date of reporting differs from the residential address of the case at disease onset (referred to as current address in the NNIDRS), regardless of their motivation of movement (business, tourism, medical consultation, etc.). According to the National Guidelines of Infectious Diseases Report (http://cdcp.gd.gov.cn/zwgk/jsbzywj/content/post_3437495.html), the current address of an individual case in the NNIDRS refers to his/her residential place at the disease onset. The detailed definition of current address of an individual case is given in Appendix (p 2); otherwise, the patient is considered a local case.^17^^,^^18^^,^^23^ Data on inter-city human mobility among the 337 prefectural cities in China were obtained by web crawling from AMAP (https://trp.autonavi.com/migrate/page.do), which is the biggest location-based data provider in China (Appendix p 35). Inter-city labor flow intensity was calculated using the Microdata of 2015 (1% Population Survey of China) provided by the National Bureau of Statistics (https://microdata.stats.gov.cn/#/). Specifically, the calculation was based on the geographical information of the migrant workers who had left their hometowns and worked in other cities for more than six months (Appendix p 36). The maps depicting the distributions of infectious diseases were based on the map of China (reference number: GS (2022) 1873) obtained from the official website of the Ministry of Civil Affairs (http://xzqh.mca.gov.cn/map). Other demographic and socioeconomic data about the cities involved in the analysis were also collected. Details of data collection and processing are given in the Appendix pp 2 and 3.

Written informed consent was waived by the National Health Commission of China for the surveillance of notifiable infectious disease. All identifiable personal information was removed from the data by China CDC before any analysis. This study was approved by the Institutional Review Boards of the Academy of Military Medical Science (IRB number: AF/SC-08/02.343).

### Descriptive analysis

The overall proportion of migratory cases is defined as P=M/N×100% where M is the number of migratory cases, and N is the total number of NID cases reported to the NNIDRS. Migratory proportions were calculated for different cities, time periods, age and sex groups, and specific types of infectious diseases, respectively. We also mapped city-specific inflow and outflow proportions of the NIDs. Inflow proportion of a given city is the ratio of the number of patients who were reported in the city but had onset elsewhere to the total number of patients who were reported in the city. Outflow proportion is the ratio of the number of patients who had symptom onset in the city but were reported elsewhere to the total number of patients who had symptom onset in the city. Specifically,

$$P_{inflow,i}=\frac{\sum_{j\neq i}^{n}N_{j,i}}{\sum_{j\neq i}^{n}N_{j,i}\;+\;N_{i,i}}\;\times \;100\%$$

$$P_{outflow,i}=\frac{\sum_{j\neq i}^{n}N_{i,j}}{\sum_{j\neq i}^{n}N_{i,j}\;+\;N_{i,i}}\;\times \;100\%$$

where $N_{i,j}$ is the number of cases who had symptom onset in city i and were reported in city j, and n is the total number of cities. $\sum_{j\neq i}^{n}N_{j,i}$ and $\sum_{j\neq i}^{n}N_{i,j}$ represent the total number cases imported to and exported from city of i, respectively.

In addition, we compared delays from disease onset to diagnosis and case fatality rates between migratory cases and local cases as a whole as well as between the following finer groups: (i) migratory cases from non-capital cities to capital cities, likely representing patients seeking better healthcare quality; (ii) all other migrant cases; (iii) local cases in capital cities; and (iv) local cases in non-capital cities.

### Social network analysis

Out of the 44 NIDs, fourteen had >10,000 domestic migratory cases and accounted for 98.10% of all migratory cases reported to NNIDRS. We constructed city-level networks to study the inter-city migration of these 14 major NIDs, where cities align with prefectures (a prefecture has several counties) in the Chinese administrative division system. In each network, a node refers to a city, a directed edge indicates existence of migratory cases who had symptom onset in the origin city and were reported in the destination city, and the edge weight reflects the number of migratory cases. The following summary statistics and methods were used to characterize the features of infectious disease migration networks. A brief introduction of the social network analysis and the network statistics used were present in the Appendix pp 3, 4 and 37.

*Node Strength* reflects how active a node is in a weighted network in term of the weighted sum of edges connected to other nodes,^24^ and is defined as,

$$S_{i}=\sum_{j=1,j\neq i}^{n}w_{ij}\;+\;w_{ji}$$

where n is the number of nodes (cities) in the network, $w_{ij}$ is the weight of the directed edge from node i to node j, and $w_{ji}$ is the weight of the directed edge from node j to node i.

*Node betweenness* is the number of the shortest directed paths (i.e., the path between two nodes that minimizes the sum of the weights along the path) between all pairs of nodes that pass through an intermediary node,^24^ indicating how important a node is as a bridge for the paths the network. The betweenness centrality of the node is defined as,

$$B_{i}=\frac{1}{\left(n-1\right)\left(n-2\right)}\sum_{j=1,j\neq i}^{n}\sum_{k=1,k\neq i\neq j}^{n}\frac{g_{jk}\left(i\right)}{g_{jk}}\text{,}$$

where n is the number of nodes in the network, $g_{jk}\left(i\right)$ is the number of shortest paths between node j and node k passing through node i, and $g_{jk}$ is the total number of shortest paths between node j and node k.

*Community detection* is a procedure to study the underlying structural patterns of a network. In this study we used the Louvain algorithm to detect the potential community pattern of the infectious disease migration network (IDMN), which is an unsupervised hierarchical clustering algorithm based on optimizing the increase in modularity (defined later) when a node i is moved from its current community to a new community. This algorithm recursively merges detected communities into a single node and perform clustering on the condensed graphs.^25^ To avoid noise from weak links, we only included the edges with more than 10 migratory cases for community detection and performed a sensitivity analysis by changing this threshold to 20 (Appendix p 38). The modularity Q of a network is calculated as,

$$Q=\sum_{c}\left[\frac{W_{C,in}}{2W_{tot}}\;-\;{\gamma \left(\frac{W_{C,con}}{2W_{tot}}\right)}^{2}\right]\text{,}$$

where $W_{C,in}$ is the sum of weights of edges among the internal nodes of community C, $W_{C,con}$ is the sum of weights of all edges connecting to community C with external nodes, and $W_{tot}$ is the sum of weights of all edges in the graph. γ is the resolution parameter that affects the number of the communities, where a higher value leads to more communities. The main results of community detection in this study were produced with the default resolution at γ=1 where modularity recovered to its original definition (if resolution is less than 1, the algorithm favors larger communities, and greater than 1 favors smaller communities). We provided a Shiny website to display community detection results at different resolutions and show graded risk levels of importing and exporting infectious diseases for each city (https://huashuiguaishou.shinyapps.io/mccx/).

*Network backbone* reflects the fundamental structure of a network. We applied the disparity filter algorithm and the force-directed edge bundling algorithm to visualize the backbone structure of each IDMN. Disparity filter is a network reduction algorithm that removes unimportant links at a significance level α (in this study α = 0.05) to identify the backbone structure.^26^ The force-directed edge bundling algorithm clusters edges with similar attributes (i.e., similarity in direction and proximity) together to facilitate visual interpretation.^27^ For better visualization, edge bundling was applied to backbone edges with more than 10 migratory cases for the top 5 diseases, more than 5 cases for the top 6 to 10, and more than one for the remaining diseases, where diseases were ranked according to the numbers of migratory cases. We used packages “backbone” and “edgebundle” in R 4.2.3 to perform the disparity filter and force-directed edge bundling in this study.

### Network modeling

To identify potential driving factors for the spread of infectious diseases via human mobility, we used the eXtreme Gradient Boosting (XGBoost) model to associate the flow of migratory cases with socio-economic and geographic variables for the 14 major NIDs.^28^ We collected 12 variables to serve as the predictors, of which five are features of the edges (human mobility intensity, labor flow intensity, geographic distance, intra-province or not, and geographically adjacent or not), and six are socioeconomic features of the origin and destination nodes (per capita gross regional products [GRP], proportion of secondary and tertiary industry in local GRP, and whether the city is a provincial capital or not). The last one is the average annual disease incidence rate of the origin city. Metrics used to measure the performance of the models are common part of commuters (CPC), Pearson correlation coefficients (PCC) and root mean squared logarithmic error (RMSLE). We used the SHapley Additive exPlanations (SHAP) approach to quantify the relative importance of the potential socioeconomic and geographical factors in predicting the migratory flows of NIDs.^29^ It is a game theory method to explain the output of any machine learning model, where the core idea is to calculate the marginal contribution of features to the model's output, and then explain the “black box model” at both the global and local levels. SHAP builds an additive explanation model, where all features are considered “contributors”. For each predicted instance, the model produces a prediction value, and the Shapley value is the numerical value assigned to each feature in that instance, where a larger absolute Shapley value means higher importance. We calculated the SHAP importance by averaging the absolute Shapley values per feature across all pair of cities to estimate their contribution to the prediction of case flows:^29^^,^^30^

$$S_{j}=\frac{1}{n}\sum_{i=1}^{n}\left|\phi_{j}^{\left(i\right)}\right|,I_{j}=\frac{S_{j}}{\sum_{j=1}^{k}S_{j}}\;\times \;100\%$$

where $\phi_{j}^{\left(i\right)}$ is the Shapley value of feature j on the $i^{th}$ pair of cities, $S_{j}$ is the mean absolute Shapley value of feature j across all pair of cities, and $I_{j}$ is the normalized $S_{j}$ to reflect the relative contribution.

As an alternative, we also fitted an ordinary gravity model to the same data and compared its predictive performance with the XGBoost model. More details on the network modeling approach are given in the Appendix pp 4−6 and 8−13.

### Role of the funding source

The funders of the study had no role in study design, data collection, data analyses, interpretation of the results, or the writing of the manuscript. The corresponding authors had full access to all the data and had final responsibility for the decision to submit for publication.

## Results

### Epidemiological characteristics

From 2016 to 2020, the NNIDRS reported 32,621,414 lab-confirmed or clinically diagnosed cases of the 39 types (44 subtypes) of NIDs in total across the mainland of China, of whom 2,674,892 (8.20%) cases were determined as inter-city migratory cases according to different addresses at the times of disease onset and reporting.

The top five diseases with the largest number of migratory cases were hepatitis B (771,423 cases; accounting for 15.02% of all reported cases for hepatitis B), tuberculosis (469,204 cases; 11.73%), HFMD (349,092 cases; 3.71%), syphilis (310,715 cases; 12.69%) and influenza (218,749 cases; 3.51%), accounting for 79.23% of the overall migratory cases (Table 1 and Appendix pp 14 and 15). The top five diseases with the highest proportions of migratory cases were all zoonotic or vector-borne, including malaria (99.98%, 12,630/12,632), kala-azar (49.91%, 537/1076), plague (41.67%, 5/12), Japanese encephalitis (40.68%, 2004/4926) and hydatid disease (37.89%, 8934/23,578). The lowest proportions were found for acute hemorrhagic conjunctivitis (1.35%, 2396/177,229), mumps (2.39%, 26,739/1,117,231) and shigellosis (2.53%, 11,606/458,685).

**Table 1:** Number and proportion of migratory cases of 44 notifiable infectious diseases in the mainland of China during 2016–2020.

Rank^a^	Disease	Total number of cases	Migratory cases		Type of migration		
			Number	Proportion^b^	Intra-province	Inter-province	From abroad
**1**	Hepatitis B	5,136,056	771,423	15.02%	561,274	205,884	4265
**2**	Tuberculosis	3,999,387	469,204	11.73%	342,723	123,171	3310
**3**	Hand-foot-and-mouth disease	9,409,017	349,092	3.71%	245,308	97,264	6520
**4**	Syphilis	2,447,645	310,715	12.69%	189,334	117,416	3965
**5**	Influenza	6,230,343	218,749	3.51%	134,005	67,619	17,125
**6**	Hepatitis C	1,109,374	144,945	13.07%	105,008	36,796	3141
**7**	HIV/AIDS	803,553	138,995	17.30%	68,967	53,926	16,102
**8**	Gonorrhea	612,682	77,938	12.72%	38,433	37,835	1670
**9**	Brucellosis	220,077	39,271	17.84%	29,793	9405	73
**10**	Mumps	1,117,231	26,739	2.39%	18,327	7477	935
**11**	Hepatitis E	133,935	22,049	16.46%	16,221	5634	194
**12**	Pertussis	72,710	18,243	25.09%	12,244	5956	43
13	Malaria^d^	12,632	12,630	99.98%	0	0	12,630
**14**	Shigellosis	458,685	11,606	2.53%	6566	4542	498
**15**	Hepatitis A	91,265	10,893	11.94%	8248	2460	185
16	Hemorrhagic fever with renal syndrome	49,844	9903	19.87%	8306	1582	15
17	Scarlet fever	311,635	9260	2.97%	5794	2976	490
18	Hydatid disease	23,578	8934	37.89%	7567	1355	12
19	Measles	38,999	6250	16.03%	4666	1340	244
20	Dengue	37,993	4251	11.19%	1789	516	1946
21	Typhoid	37,969	3291	8.67%	2428	770	93
22	Acute hemorrhagic conjunctivitis	177,229	2396	1.35%	1491	798	107
23	Japanese encephalitis	4926	2004	40.68%	1460	504	40
24	Rubella	44,820	1702	3.80%	1190	470	42
25	Paratyphoid	11,062	963	8.71%	737	202	24
26	Kala-azar	1076	537	49.91%	407	129	1
27	Rabies	2084	484	23.22%	364	117	3
28	Amoebic dysentery	4690	470	10.02%	336	115	19
29	Typhus	5329	429	8.05%	357	56	16
30	Leprosy	2201	404	18.36%	304	97	3
31	Schistosomiasis	6958	202	2.90%	169	30	3
32	Anthrax	1549	188	12.14%	157	31	0
33	Hepatitis D	1734	188	10.84%	146	40	2
34	Avian flu H7N9	864	166	19.21%	120	46	0
35	Leptospirosis	1225	160	13.06%	133	27	0
36	Epidemic cerebrospinal meningitis	489	121	24.74%	96	21	4
37	Neonatal tetanus	457	88	19.26%	63	24	1
38	Plague^c^	12	5	42%	2	3	0
39	Cholera	96	4	4%	1	3	0
40	Diphtheria^c^	2	0	0%	0	0	0
41	Avian flu H5N1^c^	1	0	0%	0	0	0
42	Filariasis	0	0	–	0	0	0
43	Poliomyelitis	0	0	–	0	0	0
44	Severe acute respiratory syndrome	0	0	–	0	0	0
**Total**		32,621,414	2,674,892	8.20%	1,814,534	786,637	73,721

a Ranked by the disease specific number of migratory cases.

b Proportion of migratory cases in all reported cases for each specific notifiable infectious disease.

c The migratory proportion of diseases with few cases (≤20 cases) might not be reliable.

d The last indigenous malaria case was reported on March 17, 2016 in Yingjiang County, Yunnan Province, China. “–” indicates that the total number of reported cases is 0.

**Fig. 1:**
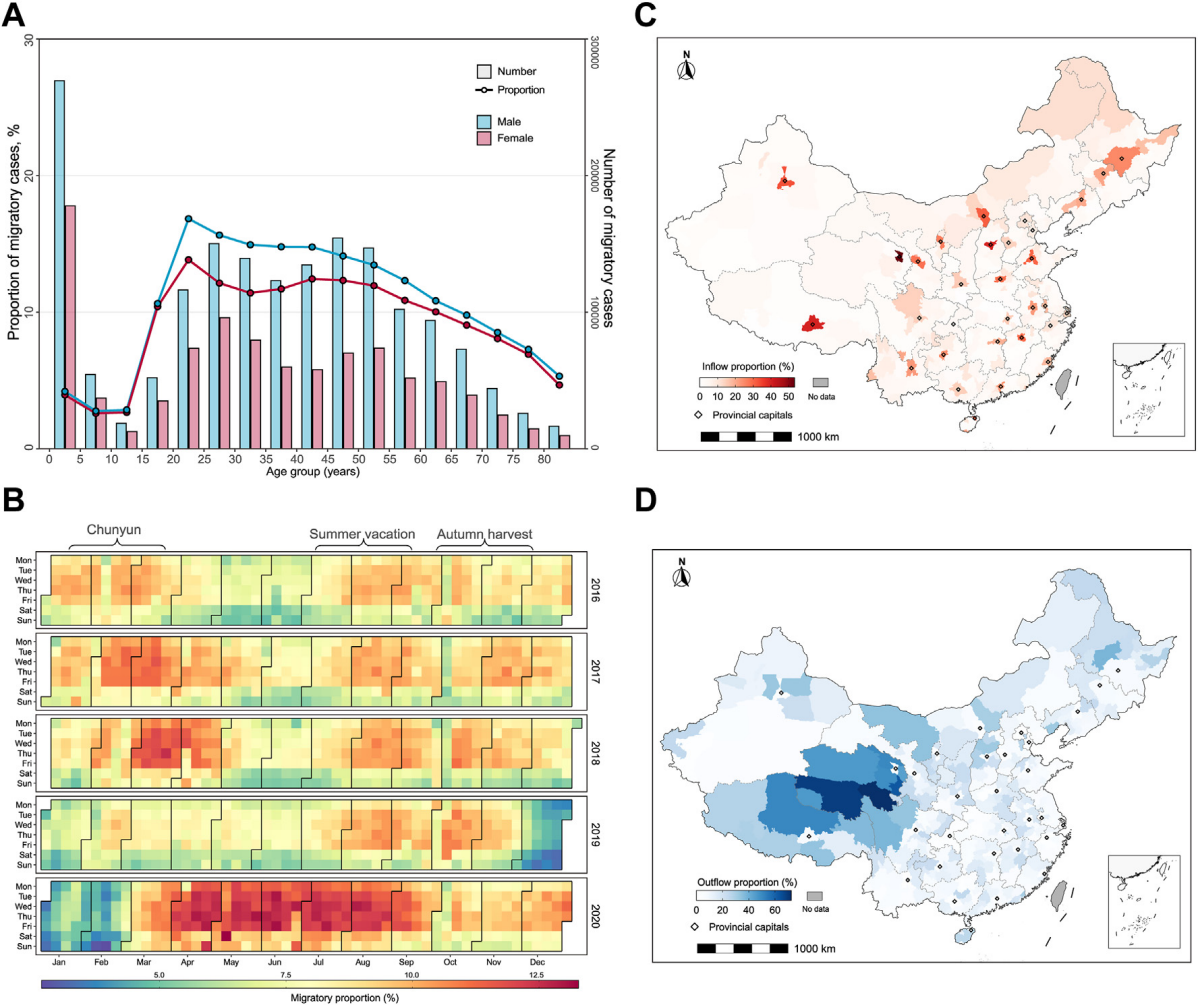
**Characteristics of migratory cases of notifiable infectious diseases during 2016**–**2020 in China.** (**A**) Numbers (connected dots) and proportions (bars) of migratory cases by sex and age group; (**B**) Temporal distribution of proportions of migratory cases by day of the week; (**C**) Spatial distribution of proportion of inflow cases among all reported cases at the city level; and (**D**) Spatial distribution of proportion of outflow cases among all reported cases at the city level. Centers of provincial capital cities are marked by dots in (**C**) and (**D**).

Combining all NIDs, children under five had the largest number of migratory cases among all five-year age groups, but working-age adults (16–60 years old) had the highest proportions (10.41%–16.84%) of migratory cases. Male patients were more likely to be migratory cases than female patients (Fig. 1A and Appendix p 16). For most diseases, migratory cases tended to be slightly younger than local cases; in particular, there were more children under 2 years old among the migratory cases for diseases dominated by pediatric infections such as influenza, HFMD and pertussis (Appendix p 39). The top 3 occupation categories associated with the highest number of cases exhibited a high degree of similarity between migratory cases and local cases, showing only minor variations in their respective rankings (Appendix pp 17−19). Farmer was the leading occupation for most diseases. In general, temporal peaks of proportions of migratory cases coincided with the travel rush periods associated with traditional holidays including lunar new year (also called Chunyun), summer vacation and the harvesting season in China in each year (Fig. 1B). The pattern is most obvious for acute infections like influenza, HFMD, mumps, and shigellosis (Appendix p 40). The proportions of migratory HIV/AIDS and pertussis cases declined from 2016 to 2019, while those of tuberculosis, gonorrhea, and hepatitis A increased (Appendix pp 20, 40, and 41). In 2020, the first year of the SARS-CoV-2 pandemic, the migratory proportions of many diseases (HFMD, brucellosis, mumps, pertussis, etc.) declined, likely a result of nonpharmaceutical interventions (Appendix p 41), but the overall migratory proportion increased notably (Fig. 1B).

We mapped the proportions of inflow and outflow cases among all reported cases in each city. Provincial capital cities had higher inflow proportions than other cities (Fig. 1C), likely due to their better socioeconomic development and access to medical resources. In contrast, less developed cities, such as those in western China, had higher outflow proportions than the rest of the country (Fig. 1D). The spatial imbalance in the inflow and outflow of migratory cases is similar across diseases, though the exact cities with high outflow proportions may differ (Appendix pp 42−45). Cities with high outflow proportions for brucellosis are mainly in the north, while those for pertussis are in central and eastern China (Appendix pp 42−45). Compared to locally reported cases, migratory cases had longer delays from symptom onset to diagnosis for pertussis and hepatitis C, and shorter delays for tuberculosis, brucellosis and HIV/AIDS (Appendix p 21). They also had higher case fatality ratios (CFR) for influenza, pertussis and shigellosis, and lower CFRs for tuberculosis and HIV/AIDS (Appendix p 22). A look into finer grouping of migratory and local cases by city type (provincial capital vs. noncapital) revealed that the longer delays in the diagnosis of pertussis and hepatitis C for migratory cases mainly occurred in those traveling from noncapital cities to capital cities, and the longer delays in the diagnosis of tuberculosis and HIV/AIDS for local cases mainly occurred in those living in noncapital cities (Appendix p 23). The higher CFR of influenza among migratory patients was driven by those traveling from noncapital cities to capital cities, but this is not the case for pertussis and shigellosis (Appendix p 24). While migratory HIV/AIDS patients showed an overall lower CFR than local patients, the CFR was actually as high among migratory patients traveling from noncapital to capital cities as among local patients in noncapital cities. For hepatitis B, hepatitis C and hepatitis E, the CFRs were clearly led by local patients in capital cities (Appendix p 24), despite the similarity in the overall CFR of between migratory and local patients (Appendix p 22). For hepatitis E, the CFR was also relatively high among migratory patients from noncapital cities to capital cities.

### Network patterns

During 2016–2020, 97.24% (2,601,171/2,674,892) of migratory cases were domestic, of which 69.76% (1,814,534/2,601,171) were intra-provincial migrants. We conducted a social network analysis on fourteen infectious diseases that accounted for 98.10% (2,551,836/2,601,171) of all the domestic migratory cases (Table 1). For each of the 14 diseases, the associated IDMN covered almost all of the 337 major Chinese cities (Table 2). The network density was positively associated with the number of migratory cases across the 14 IDMNs (Pearson *r* = 0.870, *P* < 0.001). The highest network density of 0.279 was found for hepatitis B, i.e., migration of hepatitis B cases was observed in 27.9% of the 337 × 336 directional city pairs), followed by syphilis (0.236) and tuberculosis (0.196; Table 2). Regardless of disease type, the distributions of the node degree (total number of directed edges) and node strength (total number of migratory cases, inflow and outflow) were highly skewed to the right in all the IDMNs, suggesting a large proportion of migratory cases were associated with only a few cities (Appendix p 46). In terms of node strength and node betweenness, provincial capital cities showed much higher influence than other cities for all the 14 major IDMNs (Appendix p 25). The top 20 cities in terms of node strength and node betweenness for each disease are listed in Appendix pp 26−32. By node strength, Chengdu, Changsha, Zhengzhou, Guangzhou, and Chongqing were among the top 20 for most (11−12 out of 14) major NIDs. By node betweenness, Chongqing, Shenzhen, Chengdu, Beijing, and Xi’An were ranked among the top 20 for most (11−14) major NIDs. Of these highly ranked cities, Beijing and Chongqing are administratively parallel with provinces, and the rest are provincial capitals except for Shenzhen which is one of the most economically developed cities in China.

**Table 2:** Characteristics of migration networks for 14 major notifiable infectious diseases in the mainland of China during 2016–2020.

Disease	Domestic migratory cases	Nodes	Edges	Density	City clusters^a^	Modularity
Hepatitis B	767,158	337	31,561	0.279	15	0.769
Tuberculosis	465,894	337	22,230	0.196	17	0.806
HFMD	342,572	337	14,931	0.132	19	0.838
Syphilis	306,750	337	26,683	0.236	15	0.752
Influenza	201,624	337	14,285	0.126	14	0.790
Hepatitis C	141,804	337	13,970	0.123	16	0.831
HIV/AIDS	122,893	337	18,214	0.161	15	0.718
Gonorrhea	76,268	337	15,010	0.133	18	0.703
Brucellosis	39,198	330	2664	0.025	18	0.805
Mumps	25,804	337	5099	0.045	23	0.894
Hepatitis E	21,855	335	3294	0.029	20	0.867
Pertussis	18,200	315	1127	0.011	18	0.814
Shigellosis	11,108	337	3412	0.030	22	0.906
Hepatitis A	10,708	337	2374	0.021	21	0.885

a The number of city clusters, where each cluster contains ≥3 cities.

**Fig. 2:**
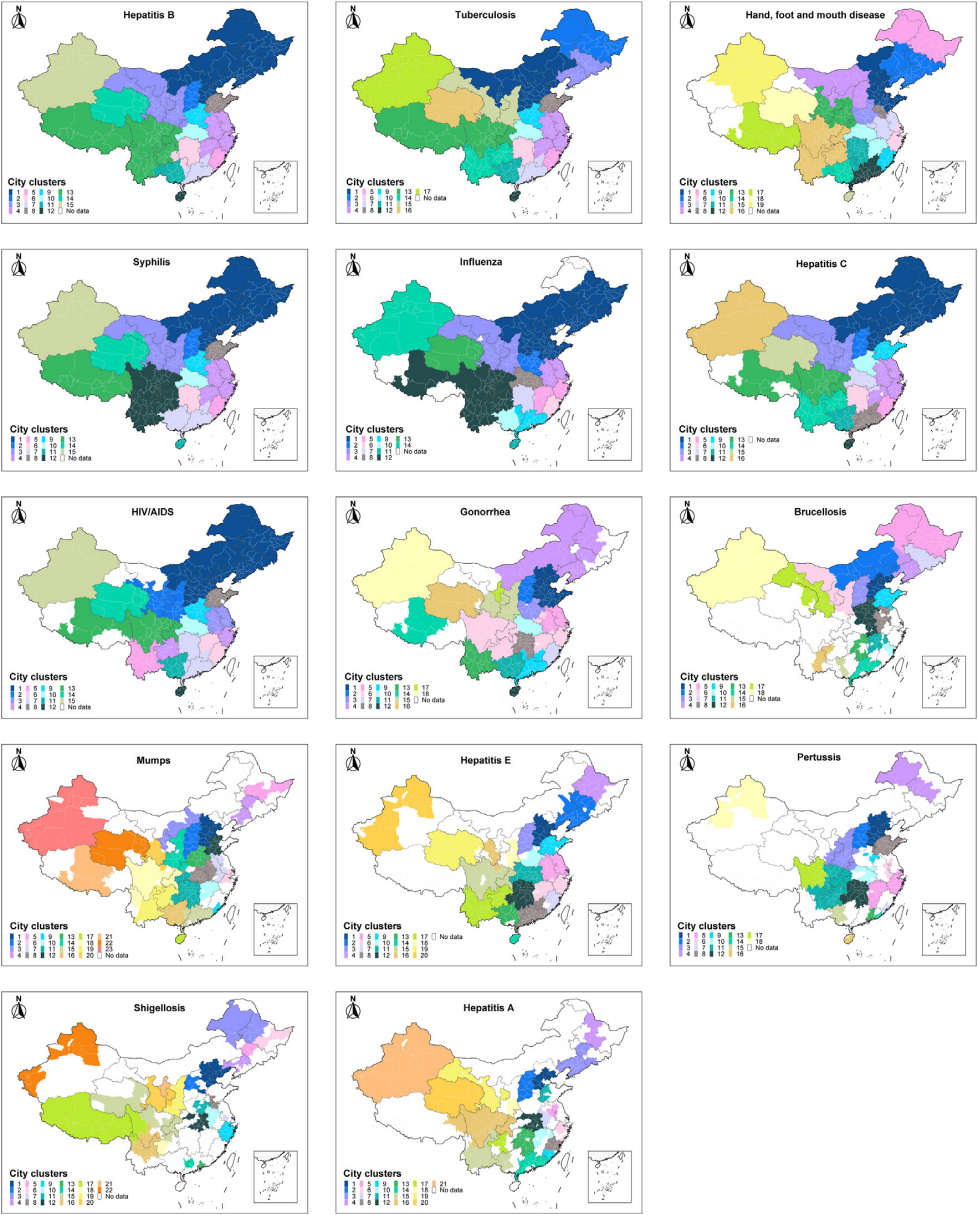
**Network community structure for fourteen major infectious diseases.** Cities in the same community are colored the same, representing a sub-network.

We further identified clustering patterns of the cities in these IDMNs. For most diseases, the community structure of the IDMNs largely coincided with the geographic structure of adjacent provinces (Fig. 2). The overall community structures of chronic diseases (Hepatitis B, Tuberculosis, syphilis, Hepatitis C, and HIV/AIDS) more or less resemble each other. For the two major sexually transmitted diseases, gonorrhea and HIV/AIDS, inland cities of Guizhou Province in Southwest China and coastal cities of Zhejiang Province in East China were clustered together. The clustering pattern of each city for each major infectious disease can be found on an interactive Shiny website (https://huashuiguaishou.shinyapps.io/mccx/).

**Fig. 3:**
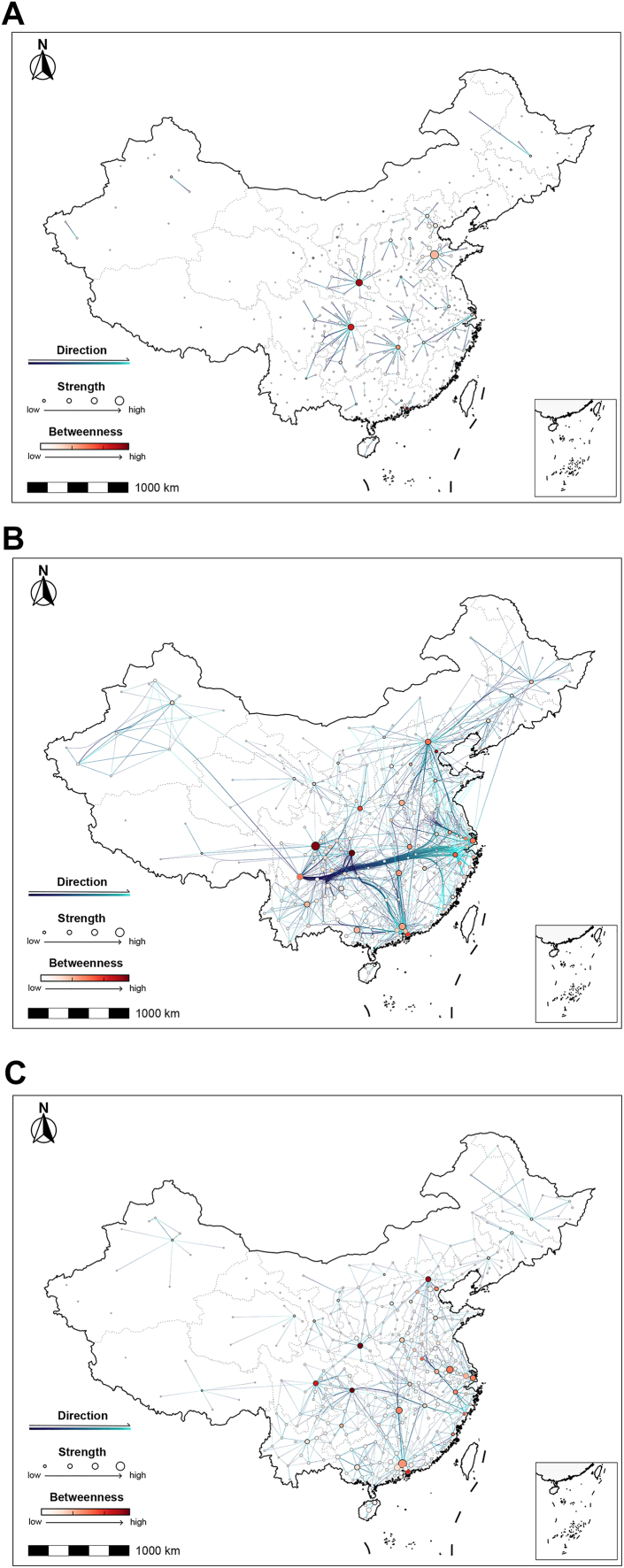
**Network backbones of migration networks for (A) pertussis, representing the short distance migration mode, (B) HIV/AIDS, representing the long distance migration mode, and (C) hand, foot and mouth disease, representing the mixed mode.** The color of each directed edge changes from dark blue to bright blue to represent the direction of migration from the origin to the destination. The size of each node (city) represents the node strength of the city in the migration network. Each node is colored by the node betweenness in the network, with darker red indicating a larger value of node betweenness.

A visualization of the backbones of the IDMNs revealed three typical modes of major connections. (1) The short-distance mode represented by pertussis, brucellosis, mumps, hepatitis E, hepatitis A and Shigellosis (Fig. 3A and Appendix p 47), where backbone paths were mostly either between adjacent cities within a province or between capital cities of adjacent provinces. (2) The long-distance mode represented by HIV/AIDS, tuberculosis, syphilis, and gonorrhea (Fig. 3B and Appendix p 48), where bundled directed paths connected cities in Southwest and Central China to the coastal economically-developed urban agglomerations in the Yangtze River Delta near Shanghai and the Pearl River Delta around Guangzhou. (3) The mixed mode seen for HFMD, hepatitis B, influenza, and hepatitis C (Fig. 3C and Appendix p 49), where a mixture of short-distance and long-distance modes coexisted. Long-distance connections between Beijing and northeastern provincial capitals such as Harbin and Changchun are observed for tuberculosis, syphilis, hepatitis B, influenza, and hepatitis C.

### Socioeconomic and geographical drivers

**Fig. 4:**
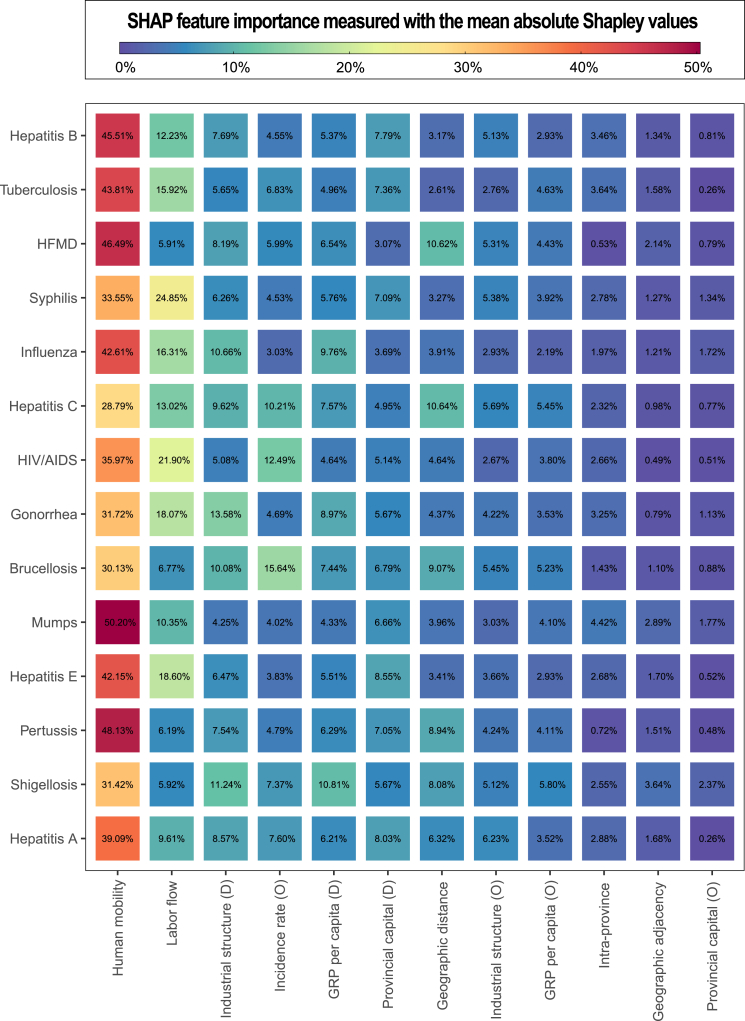
**The SHAP feature importance of predictors for infectious disease migration.** The numbers in the grids represent the relative SHAP importance (measured as the mean absolute Shapley values) of the XGBoost models, scaled to sum up to 100% for each row. The grids are also colored in red (blue) for high (low) relative importance.

To identify potential drivers for the migration of NID cases, we further associated the structure of the IDMNs with urban indicators using regression models. The XGBoost model outperformed the ordinary gravity model for all the 14 major diseases as shown by multiple metrics based on the test data (Appendix p 33) and goodness of fit based on all data (Appendix pp 50−52). Here we describe important drivers for disease migration as factors with mean absolute Shapley values ≥ 10% based on the XGBoost models (Fig. 4). The exact association, particularly the direction of such association, of each risk factor with the SHAP values are shown in Appendix pp 53−61. The most relevant feature for the migration of human cases was human mobility intensity, contributing 29–50% to all the 14 diseases, which is not unexpected as large-scale between-city human mobility increased the chance of the migration of patients of infectious diseases. Labor flow intensity was another important driver, contributing 10−25% to the migration of sexually transmitted or bloodborne (HIV/AIDS, syphilis, gonorrhea, HBV and HCV), air-borne (TB, influenza, mumps), and fecal-oral (HEV) diseases. Geographical distance was negatively associated with the migration of HFMD and HCV. Incidence rate of the origin city was positively associated with the migration of HCV, HIV/AIDS and brucellosis. GRP per capita of the destination city was positively associated with the migration of shigellosis. Relative contributions of binary indicators are usually below 10%, but these indicators clearly differentiate the travel frequency of patients (Appendix p 61). Migration of cases was more frequent between pairs of cities with the following features: within the same province, spatially adjacent cities and either one being the provincial capital city.

## Discussion

Over four decades of modernization, China has experienced a significant increase in human movement, which has inevitably facilitated the spread of infectious diseases. In addition, the geographical imbalance in economy and accessibility to healthcare may have fueled the migration of patients. Utilizing the surveillance data from the NNIDRS, we conducted a comprehensive network-based analyses on the migration patterns of major notifiable infectious diseases and associated driving factors in China. The goal if our study was to inform programs and policies for preventing and controlling spread of infectious diseases and reducing geographic disparity in healthcare resources. Modern network analysis methodology has been applied in infectious diseases epidemiology including but not limited to monitoring key populations with HIV, tracking transmission of influenza, and detecting pre-outbreak signals of HFMD.^31^, ^32^, ^33^ Our study demonstrated its utility in modeling patient mobility when coupled with national surveillance data.

During 2016–2020, 8.20% of NID cases identified in China were inter-city migratory cases. The proportion of cases which were identified as migratory is exceptionally high for zoonotic and vector-borne diseases such as brucellosis, hydatid disease, kala-azar, and plague, which is closely related to inequality in local healthcare accessibility. These diseases had high incidences in under-developed areas where disease diagnosis and treatment capacities are inadequate, leading to a high frequency of cross-regional healthcare-seeking behavior.^34^, ^35^, ^36^ Key intervention measures should be focused on the radiation and sinking of quality healthcare resources, which resonates with the recent policy of the Chinese government on reducing geographical disparities in healthcare accessibility as parts of the efforts in advancing the nation’s modernization (https://www.gov.cn/zhengce/202407/content_6963770.htm). High proportions of migratory patients were also found for some vaccine-preventable pediatric diseases such as pertussis, measles, Japanese encephalitis, epidemic cerebrospinal meningitis, and neonatal tetanus. Routine vaccination of children is required for these infectious diseases, according to the Chinese Expanded Program of Immunization. However, the coverage of vaccination in migrant children was relatively low and often delayed.^37^

Our network analysis demonstrated strong connections between several developing inland cities in southwestern China and developed coastal cities in the Yangtze River Delta and the Pearl River Delta in the migration networks of TB, syphilis, HIV/AIDS, and gonorrhea (Appendix p 35). Additional connections with the Yangtze River Delta can be traced back to a cluster of cities near the border between Henan Province and Anhui Province, which are the two main providers of labor workers to Shanghai, the center of the Yangtze River Delta.^38^ Such disease flows are consistent with labor force flows driven by geographic disparity in economic development.^39^ Due to the synergy of relatively poor living conditions, lack of knowledge about STI prevention, and lack of STI clinics in their home villages, migrant labor workers are known to be at high risk of sexually transmitted diseases and tuberculosis and are often diagnosed and reported in cities where they work.^40^^,^^41^ Since 2016, China’s healthcare insurance system underwent a major reform, streamlining the reimbursement process for medical expenses across provinces. This reform has further facilitated inter-regional mobility of patients and improved access to healthcare services for migrant workers whose health insurances are usually provided by agencies at their hometown.^42^^,^^43^

Long-term temporal trends in the proportions of migratory cases of the NIDs reflect the impact of or need for control policies. From 2016 to 2019, migratory proportions of HIV/AIDS and pertussis decreased, likely benefitting from increased HIV screening and routine vaccination against *Bordetella pertussis* in the migrant workers and their families. On the other hand, growing proportions of migratory cases of tuberculosis, gonorrhea, and hepatitis A indicate the need for strengthening the surveillance and control of these diseases among the migrant population. Some temporal changes warrant careful interpretation. In 2020, a rise in the overall proportion of migratory cases among reported cases of NIDs was observed in the first half of the year. The most likely reason is that the intensive nonpharmaceutical interventions (NPI) for controlling the COVID-19 pandemic changed the spectrum of other diseases (Appendix p 62).^44^ Specifically, acute infectious diseases with low proportions of migratory cases, such as influenza and HFMD, had a sharp decrease under NPIs, whereas chronic infections with relatively high migration proportions, like hepatitis B and tuberculosis, were much less sensitive to the NPIs, leading to an overall increase in the proportion of migratory cases.

We observed that migratory cases exhibited notably higher CFRs for several acute NIDs including HFMD, influenza, pertussis, and shigellosis, but lower CFRs for two chronic NIDS, tuberculosis and HIV/AIDS. The higher CFR of the acute diseases in migratory patients could be partly associated with referral or transfer of severe patients from clinics in resource-limited regions to better equipped hospitals in metro cities. Given that HFMD, influenza and pertussis disproportionally affect children and are vaccine-preventable, a helpful addition to the general policies addressing geographic disparity in healthcare services would be to increase vaccine coverage among children in migrant families. The lower CFR of the chronic NIDs in migratory patients could be related to better quality of care and availability of more advanced treatments in large cities, e.g., treatments for multidrug-resistant TB are often lacking at county-level health facilities.^45^ In addition, a previous study found that a large proportion of inter-provincial migratory TB patients were working age males traveling from inland cities to developed coastal cities, suggesting that these patients were likely healthier than local TB patients.^46^ However, the lower CFR of migratory HIV/AIDS patients should be interpreted with caution, as we observed that the CFR of HIV/AIDS patients traveling from non-capital to capital cities was as high as local cases in noncapital cities (Appendix p 24), despite better health care in capital cities. Henceforth, these traveling patients, together with local patients in noncapital cities, might have been associated with more progressed AIDS stages and should be prioritized for antiretroviral treatments.

A few recent changes in health policies have affected the migration patterns of infectious disease patients in China. For example, the increasing availability and affordability of pre-surgical tests and public health screening campaigns for HBV, HCV, HIV, and *Treponema pallidum* have facilitated early detection and treatment of these bloodborne and sexually transmitted diseases, especially among migrants. Detections of latent syphilis via public health screening or routine pre-surgical tests accounted for 79.55% of all reported syphilis cases during 2016–2020.^47^, ^48^, ^49^ In addition, the Chinese government has been actively improving the capacity of primary healthcare services and building regional medical centers to alleviate the geographic disparity in medical resources.^50^^,^^51^ However, these fragmented and localized strategies may not be adequate to fully address the challenges raised by large-scale human movement. Given the infectious disease migration network we identified, we encourage policy-makers to foster close collaborations in prevention and control programs between major case-exporting and case-importing cities or provinces, for example, on top of streamlined health insurance payment systems across regions, to create channels for sharing medical and vaccination records to better serve migratory patients. For sexually transmitted diseases, educational programs on disease prevention and physical examination programs targeting migrant workers could be co-sponsored by labor-exporting and labor-importing cities.

A few limitations of our work should be acknowledged. First, current findings might have been biased by limitations in data quality. Recall bias is common for diseases with a long incubation period, e.g., TB and HIV, and self-reported residential addresses could be false for sensitive diseases such as STDs. Second, intermediate cities that each patient might have visited along the trip between the origin and the destination are unknown. However, such visits are primarily transient and thus unlikely to severely affect our findings about the key structural features of the IDMNs. Third, the surveillance database NNIDRS did not capture specific symptoms or disease stage of each patient, making it difficult to assess the risk of onward transmission during and after traveling. In addition, the flows for NID patients cannot be equated to inter-city transmission risks, as diseases are often transmitted by asymptomatic individuals or mild cases who do not seek healthcare and are thus not reported by the surveillance system; in particular, vector-borne diseases such as malaria and dengue would require established ecological niches of relevant mosquito species, which are absent in most northern cities of China. Moreover, although we explored a few potential driving factors for the migration of NID patients using an ecological modeling approach and SHAP importance, ecological fallacies are inevitable as the analysis was done at the city level, and no causal interpretation should be made. Finally, the inter-city labor flow intensity used in our analysis was based on survey data from 2015, which may not reflect changes from 2016 to 2020. More recent data from the seven^th^ census in China (2020) has not yet been publicly released yet. The validity of our results is nevertheless supported by the fact that the travel patterns of migrant workers remained largely similar from 2015 to 2020, according to the summary statistics on inter-city labor flow provided in the from annual monitoring survey reports of the National Bureau of Statistics (Appendix p 35).

While human mobility is the primary driver for the spread of most infectious diseases, control strategies should factor in the differential migration patterns of patients closely related to the underlying transmission mechanisms and clinical features of the diseases. This study underscores the importance of ensuring equitable access to primary healthcare services and calls for collaborative efforts in screening and caring for migratory patients between cities bearing the heaviest patient flows.

## Contributors

Conceptualization: LJY, YY, LQF, PSJ, WL, XR, SIH, LPW.

Methodology: LJY, TW, YHW, LCL, YT, YY, PSJ.

Data collection: LJY, XR, LCL, JJC, TT, YHW, YQS, YNL, MCL, TTL, YBQ, CXS, QX, SHL, LPW.

Visualization: LJY, TW, JJC, YT.

Supervision: YY, LQF, WL, LPW.

Writing—original draft: LJY.

Writing—review & editing: LQF, YY, WL, YHW, LPW, SIH, PSJ.

## Data sharing statement

Raw data of reported cases for each notifiable infectious disease are not publicly available and are protected due to data privacy laws. De-identified and aggregated data may be requested from the corresponding author (Dr. Li-Qun Fang) with permission from the data provider (Li-Ping Wang). The authors do not have permission to share the inter-city human mobility data provided by AMAP, and data requests should be directed to the company (https://lbs.amap.com/).

## Editor note

The Lancet Group takes a neutral position with respect to territorial claims in published maps and institutional affiliations.

## Declaration of interests

Authors declare that they have no competing interests.
