# Supplementary material for: Inter-city movement pattern of notifiable infectious diseases in China: a social network analysis
Source: Lancet Reg Health West Pac. 2024 Dec 13;54:101261. doi: 10.1016/j.lanwpc.2024.101261 (PMC11700286; doi:10.1016/j.lanwpc.2024.101261)
Supplement: Appendix [file mmc1.docx]

**Supplementary appendix**

Supplement to: Inter-city Movement Pattern of Notifiable Infectious Diseases in China from A Network Perspective

**Contents**

Supplementary Text 2−6

Supplemental References 7

Supplemental Tables 8−34

Supplemental Figures 35−62

**Supplementary Text**

**Data collection and manipulation**

***The surveillance system***

The National Notifiable Infectious Disease Reporting System (NNIDRS) is an internet-based real-time reporting system for 39 types/subtypes of notifiable diseases in mainland China, as mandated by the Law of the People’s Republic of China on the Prevention and Treatment of Infectious Diseases.^1^ This system covers over 168 000 health facilities in all prefectural cities in mainland China (http://www.gov.cn/xinwen/2022-06/21/content_5696881.htm). The standard diagnosis criteria for these notifiable infectious diseases are shown in appendix pp 14 and 15. Standardized data on individual cases are transmitted electronically from hospitals, local CDC offices, community health centers, township health centers and village clinics to a central database located at the China CDC. Demographic data such as name, sex, age, and residence, clinical data such as dates of symptom onset, diagnosis, hospital visit, and death, and laboratory results (if available) are first logged into hospital electronic health record systems by physicians or trained hospital staff, which are then reviewed and formally uploaded to the NNRDS by the staff. These data are further verified by staff at local CDC for mistakes, omissions, and duplications. Some laboratory tests are performed or confirmed by local CDC. In addition to patient data, reporting physicians and hospitals and reporting, updating, review and finalizing dates are also recorded in the NNIDRS.

***Definition of current address of an individual case in the NNIDRS***

In this study, the current address of an individual case in the NNIDRS, as defined by the National Guidelines of Infectious Diseases Report (http://cdcp.gd.gov.cn/zwgk/jsbzywj/content/post_3437495.html), refers to his/her residential location at the disease onset. Specifically, (1) for patients who travel for medical consultation or treatment, the current address should be the patient's permanent residential residence (e.g., for a patient with the hand, foot and mouth disease who traveled from city A to city B for medical treatment, the current address should be his/her residential address in city A.); (2) for cases whose illness occurred during trips, the current address should be the address of the temporary address at illness onset; (3) for emerging infectious disease cases imported from abroad who cannot provide their current address, the current address should be the address of the reporting hospital, but the country of origin is captured by the NNIDRS; and (4) for cases of people in custody or serving a sentence, the current address should be filled in with the address of their place of custody or serving a sentence.

***Inter-city human mobility***

Data on inter-city human mobility in China were obtained by web crawling from the websites of AMAP (https://trp.autonavi.com/migrate/page.do). The AMAP human mobility index is a summary indicator for the inter-city movement records of users of the location-based service offered by AMAP (https://lbs.amap.com/). In this study, we obtained history data of the AMAP human mobility index during Jan 1 2019 to Mar 31 2020 as data before 2019 were not available. Considering that human mobility was largely restricted under the impact of COVID-19 non-pharmaceutical interventions, in this study we used the daily human mobility index among cities during Jan 1 2019 to Dec 31 2019 to represent the general pattern of inter-city human mobility in China (Appendix p 35).

***Inter-city labor flow intensity***

Data on inter-city labor flow intensity was extracted from the database of Microdata of 2015 1% Population Survey of China, which covered a total of 1 371 252 anonymized individual records (1‰ of the total population) with information on gender, age, ethnicity, occupation, migration flows, collected by the National Bureau of Statistics (https://microdata.stats.gov.cn/#;http://www.stats.gov.cn/zt_18555/zdtjgz/cydc/xw/202302/t20230221_1917243.htm). According to the questions #9 (time of leaving the household registration place) and #10 (reasons of leaving the household registration place) in the survey questionnaire, we identified 93 867 records as migrant workers who had left their hometown and worked in other cities for more than 6 months, and we calculated the inter-city labor flow intensities among the 337 prefectural cities based on these records (Appendix p 36).

***Social Network Analysis***

Social network analysis (SNA) is a set of methods and theories that examine the structure and patterns of social relationships, often using graph theory. It involves mapping and measuring the relationships and flows between people, groups, organizations, or other connected entities. SNA can help in understanding how these connections influence behavior, information flow, and the overall dynamics within a group or organization (appendix p 37). SNA has been widely used to understand the epidemiology of infectious diseases including disease monitoring and tracing of epidemics, including but not limited to monitoring key populations with HIV,^2^ tracking transmission of influenza,^3^ and detecting pre-outbreak signals of HFMD.^4^

In the context of a directed weighted network, we can interpret the following network statistics:

*Node Degree*

In-degree: The number of incoming edges to a node. This can be interpreted as the level of popularity or receptivity; for example, in a social media context, it would represent the number of followers a user has.

Out-degree: The number of outgoing edges from a node. This could indicate the level of activity or outreach; for instance, the number of users a person follows on a social platform.

*Node Strength*

In-strength: The sum of the weights of all incoming edges. If the weights represent, for example, the frequency or intensity of communication, in-strength would reflect the total amount of interaction a node receives.

Out-strength: The sum of the weights of all outgoing edges. This would show the total amount of interaction a node initiates or contributes to.

*Shortest Path*: In a directed weighted network, the shortest path between two nodes (often referred to as the source and the destination) is defined as the path with the minimum sum of edge weights from the source node to the destination node. The weight of an edge represents the cost, distance, or some other metric associated with traversing that edge. In the context of a transportation network, where nodes represent locations and edges represent routes between them with weights as distances or travel times, the shortest path would be the most efficient route directly from one location to another. In a communication network, nodes might represent devices or servers, and edges could represent connections with weights as latency or bandwidth. The shortest path would then be the connection with the lowest latency or highest bandwidth.

*Network Density*: Network density is the ratio of the number of actual directed edges to the total number of possible directed edges in the network. In a directed network, the maximum number of possible edges is n * (n - 1), where n is the number of nodes, because each node can have an edge to every other node, but not to itself. A high network density suggests a well-connected network where many nodes are directly linked, which may facilitate rapid diffusion of information or resources. Conversely, a low-density network indicates that there are fewer direct connections, which might slow down the spread of information or require more intermediaries for communication.

***Economic development***

We collected city-level data about per capita gross regional products (GRP) and industrial structure (measure with the proportion of secondary and tertiary industry sectors in local GRP) in 2019 to represent the economy development status of each city. We did not use data of more recent years which were much affected by the COVID-19 pandemic. GRP per capita and the proportion of secondary and tertiary industry sectors in local GRP of the cities were obtained from the statistical communiqué of local economic and social development on the website of the bureaus of statistics of each city.

**Network modeling**

***eXtreme Gradient Boosting model (XGBoost)***

XGBoost is a machine learning model belonging to the class of Gradient Boosting Decision Tree (GBDT) methods.^5^ For small to medium sized datasets, XGBoost has faster and better prediction performance than traditional machine learning models due to the change in the learning method and the addition of regular function. In our study, the XGBoost model is used to associate the flow intensity of diagnosed cases along the directed edges of the migration network of infectious diseases with features of nodes (cities). Each disease was analyzed separately. For each disease model, each pair of city contributes two data points to the model.

***Response (outcome) variables***

*Migration cases:* The total number of cases of a specific disease traveling from an origin city to a destination city during the study period.

***Explanatory variables***

Features associated with the directed city pair:

1. Human mobility intensity from the origin city to the destination city.
2. Labor flow intensity from the origin city to the destination city.
3. Geographic distance between the origin and destination cities.
4. Geographic adjacency: Whether the origin and destination cities were geographically adjacent, 1=adjacent and 0=not adjacency.
5. Intra-province indicator: Whether the two cities are within the same province, 1=yes and 0=no.
6. Features associated with each node(city): The feature names are appended with “(O)” or “(D)” to distinguish whether it is a feature of the origin city or the destination city.
7. Incidence rate (O): Annual incidence rate (per 100 000 people) of infectious diseases in the origin city.
8. GRP per capita (O) and GRP per capita (D): Gross Regional Products per capita of the origin and destination cities.
9. Industrial structure (O) and Industrial structure (D): Proportions of local GRP accounted by secondary and tertiary industry sectors of the origin and destination cities.
10. Provincial capital (O) and Provincial capital (D): Whether the origin or destination city is the provincial capital or not, 1=yes and 0=no.

***Metrics***

The CPC is a similarity measure based on the Sørensen-Dice index in ecology, computing to what extent the observed flows are correctly reproduced, on average, by the simulated network.^6^ It varies between 0, when no agreement is found, and 1, when the two networks are identical. CPC is defined as follows:

$$CPC\left( T,\tilde{T} \right)=\frac{2NCC(T,\tilde{T})}{NC\left( T \right)+NC(\tilde{T})}$$

$$NCC\left( T,\tilde{T} \right)=\sum_{i=1}^{n} \sum_{j=1}^{n} \min\left( T_{ij},\tilde{T}_{ij} \right)$$

$$NC\left( T \right)=\sum_{i=1}^{n} \sum_{j=1}^{n} T_{ij}$$

where $T$ is the observed origin-destination matrix of migratory flows and $\tilde{T}$ is the simulated one from model prediction. When $NC\left( T \right)\cong NC(\tilde{T})$, the CPC represents the percentage of connections correctly predicted by the model.

Besides, Pearson correlation coefficient ($\rho$) and RMSLE (root mean squared logarithmic error) between observed and model-predicted flows were also calculated. The RMSLE is defined as follows:

$$RMSLE=\sqrt{\frac{1}{n(n-1)}\sum_{i=1}^{n} \sum_{j=1, j\neq i}^{n} \left[ \ln\left( T_{ij}+1 \right)-\ln(\tilde{T}_{ij}+1) \right]^{2}}$$

RMSLE is a suitable metric when the data has a long-tail right-skewed distribution.

***Data split***

We randomly divided the collection of all directional city pairs into two parts and used 70% as the training set and the remaining 30% as the test set. In order to make the data roughly balanced in terms of spatial distribution of disease incidence, sampling of city pairs was weighted by the disease incidence of the origin city.

***Hyperparameter tuning***

We used the Bayesian optimization procedures with the “ParBayesianOptimization” package in R4.2.3 for fine tuning of the hyperparameters of the XGBoost models, and the hyperparameter values used in the final models were listed in appendix p 8.

***Gravity model***

As an alternative to the XGBoost models, we also fitted ordinary gravity models to the migratory flows of infectious diseases with the same features (predictors). The gravity model used in this study is defined as follows:

$$\hat{\mu_{ij}}=\exp\left( \beta_{0}+\beta_{1}\ln\left( {PopMov}_{ij} \right)+\beta_{2}\ln\left( {Labor}_{ij} \right)+\beta_{3}\ln\left( {Dist}_{ij} \right)+\beta_{4}GeoAdj+\beta_{5}IntraProv+\beta_{6}{Inci}_{i}+\beta_{7}{GRPpc}_{i}+\beta_{8}{GRPpc}_{j}+\beta_{9}{pGRP23}_{i}+\beta_{10}{pGRP23}_{j}+\beta_{11}{ProvCap}_{i}+\beta_{12}{ProvCap}_{j} \right)$$

where $\mu_{ij}$ represents the estimated mean number of migratory cases between origin city $i$ and destination city $j$. Here we used the subscripts i and j instead of “(O)” and “(D)” to indicate origin city and destination city in the variable names. The gravity models were constructed with the “gravity” package in R4.2.3. Coefficient estimates of the gravity models were listed in appendix pp 9−13.

**Model assumption of independence**

Like in typical regression analysis, both XGBoost and the gravity model assume the outcome units (case flow between each pair of cities) are independent after conditioning on predictors, i.e., the residuals are independent. Patient flow as well as the general population flow between cities are largely associated with population size, population density, economical development, and administrative status. Our predictors well represent all these key factors. We did not include population size as a predictor because it is often highly correlated with population density. After conditioning on these key factors, it is reasonable to assume the residual dependence among the outcomes is relatively weak, to a level that will not compromise the results on the statistical significance of the predictors. However, we caution readers that, if the residual dependence is strong, some of the significant findings may be false positive due to under-estimation in the variances.

**Supplemental References**

1. Wang L, Wang Y, Jin S, et al. Emergence and control of infectious diseases in China. *Lancet (London, England)* 2008; **372**(9649): 1598-605.
2. Rwabiyago OE, Katale A, Bingham T, et al. Social network strategy (SNS) for HIV testing: a new approach for identifying individuals with undiagnosed HIV infection in Tanzania. *AIDS Care* 2024; **36**(sup1):201-210.
3. Chan J, Holmes A, Rabadan R. Network analysis of global influenza spread. *PLoS Comput Biol.* 2010; **6**(11):e1001005.
4. Zhang X, Xie R, Liu Z, Pan Y, Liu R, Chen P. Identifying pre-outbreak signals of hand, foot and mouth disease based on landscape dynamic network marker. *BMC Infect Dis.* 2021; **21**(Suppl 1):6.
5. Chen T, Guestrin C. XGBoost: a scalable tree boosting system. *ACM* 2016.
6. Sørensen T. A method of establishing group of equal amplitude in plant sociobiology based on similarity of species content and its application to analyses of the vegetation on Danish commons. *Biologiske Skrifter* 1948; **5**: 1-34.

**Supplemental Tables**

**Table S1. Final fine-tuned hyperparameter values used for the XGBoost models to evaluate predictors for the migration reported cases of 14 notifiable infectious diseases.**

| **Disease** | **Hyperparameters** | | | | | | | | | |
| --- | --- | --- | --- | --- | --- | --- | --- | --- | --- | --- |
|  | $\boldsymbol{eta}$ | $\boldsymbol{nrounds}$ | $\boldsymbol{tweedie\_variance\_power}$ | $\boldsymbol{max\_depth}$ | $\boldsymbol{gamma}$ | $\boldsymbol{alpha}$ | $\boldsymbol{lambda}$ | $\boldsymbol{min\_child\_weight}$ | $\boldsymbol{subsample}$ | $\boldsymbol{colsample\_bytree}$ |
| Hepatitis B | 0·05 | 1523 | 1·47 | 5 | 8 | 1·97 | 0·73 | 67 | 0·72 | 0·98 |
| Tuberculosis | 0·05 | 2237 | 1·44 | 3 | 0 | 5·00 | 5·00 | 1 | 0·98 | 0·84 |
| HFMD | 0·05 | 685 | 1·80 | 8 | 10 | 4·90 | 4·99 | 100 | 0·60 | 0·88 |
| Syphilis | 0·05 | 1893 | 1·40 | 4 | 3 | 1·67 | 3·17 | 21 | 0·80 | 0·99 |
| Influenza | 0·05 | 2503 | 1·65 | 3 | 1 | 3·74 | 0·23 | 74 | 0·97 | 0·78 |
| Hepatitis C | 0·05 | 1517 | 1·74 | 6 | 4 | 0·17 | 2·28 | 11 | 0·85 | 0·65 |
| HIV/AIDS | 0·05 | 1163 | 1·31 | 4 | 6 | 4·30 | 4·11 | 93 | 0·87 | 0·68 |
| Gonorrhea | 0·05 | 2949 | 1·32 | 3 | 0 | 4·25 | 2·32 | 100 | 0·98 | 0·91 |
| Brucellosis | 0·05 | 1183 | 1·63 | 4 | 1 | 2·43 | 2·06 | 40 | 0·86 | 0·79 |
| Mumps | 0·05 | 550 | 1·44 | 3 | 0 | 2·13 | 3·81 | 100 | 0·60 | 1·00 |
| Hepatitis E | 0·05 | 693 | 1·09 | 3 | 3 | 2·39 | 2·54 | 5 | 0·68 | 0·96 |
| Pertussis | 0·05 | 771 | 1·67 | 3 | 4 | 3·64 | 3·71 | 35 | 0·97 | 0·85 |
| Shigellosis | 0·05 | 941 | 1·59 | 5 | 3 | 3·11 | 1·68 | 87 | 0·76 | 0·77 |
| Hepatitis A | 0·05 | 465 | 1·47 | 5 | 8 | 1·97 | 0·73 | 67 | 0·72 | 0·98 |

Annotation, (1) $eta$: Step size shrinkage used to prevents overfitting. After each boosting step, eta shrinks the feature weights to make the boosting process more conservative. (2) $nrounds$: the number of rounds for boosting. (3) $tweedie\_variance\_power$: Parameter that controls the variance of the Tweedie distribution, set closer to 2 to shift towards a gamma distribution or 1 towards a Poisson distribution. (4) $max\_depth$: Maximum depth of a tree. (5) $gamma$: Minimum loss reduction required to make a further partition on a leaf node of the tree. (6) $alpha$: L1 regularization term on weights. (7) $lambda$: L2 regularization term on weights. (8) $min\_child\_weight$: Minimum sum of instance weight needed in a child leaf node. (9) $subsample$: Subsample ratio of the training instances. (10) $colsample\_bytree$: the subsample ratio of columns when constructing each tree.

**Table S2. Coefficient estimates of the gravity models.**

| **Variable** | | **Coefficient estimate** | | **Standard error** | **z value** | ***p*-value** |
| --- | --- | --- | --- | --- | --- | --- |
| **Hepatitis B** | | | | | | |
| Intercept | | -2·651 | | 0·321 | -8·250 | <0·001 |
| Incidence rate (O) | | 0·002 | | 0·0003 | 5·697 | <0·001 |
| Human mobility intensity | | 0·622 | | 0·012 | 50·882 | <0·001 |
| Labor flow intensity | | 0·706 | | 0·021 | 33·003 | <0·001 |
| Geographic distance | | 0·211 | | 0·033 | 6·306 | <0·001 |
| Geographic adjacency | | 0·771 | | 0·114 | 6·759 | <0·001 |
| Intra-province | | 0·397 | | 0·072 | 5·494 | <0·001 |
| GRP per capita (O) | | -0·064 | | 0·004 | -14·658 | <0·001 |
| Provincial capital (O) | | 0·309 | | 0·041 | 7·500 | <0·001 |
| Industrial structure (O) | | -0·023 | | 0·002 | -11·212 | <0·001 |
| Provincial capital (D) | | 1·401 | | 0·043 | 32·867 | <0·001 |
| Industrial structure (D) | | 0·015 | | 0·002 | 7·394 | <0·001 |
| GRP per capita (D) | | -0·025 | | 0·005 | -4·802 | <0·001 |
| **Tuberculosis** | | | | | | |
| Intercept | | -6·806 | | 0·455 | -14·953 | <0·001 |
| Incidence rate (O) | | 0·005 | | 0·0003 | 15·379 | <0·001 |
| Human mobility intensity | | 0·676 | | 0·015 | 46·311 | <0·001 |
| Labor flow intensity | | 0·749 | | 0·019 | 40·075 | <0·001 |
| Geographic distance | | 0·548 | | 0·037 | 14·960 | <0·001 |
| Geographic adjacency | | 1·081 | | 0·108 | 9·974 | <0·001 |
| Intra-province | | 0·597 | | 0·078 | 7·690 | <0·001 |
| GRP per capita (O) | | -0·111 | | 0·006 | -17·510 | <0·001 |
| Provincial capital (O) | | 0·249 | | 0·066 | 3·795 | <0·001 |
| Industrial structure (O) | | -0·014 | | 0·003 | -5·583 | <0·001 |
| Provincial capital (D) | | 1·108 | | 0·044 | 25·201 | <0·001 |
| Industrial structure (D) | | 0·011 | | 0·003 | 3·291 | <0·001 |
| GRP per capita (D) | | 0·067 | | 0·007 | 9·412 | <0·001 |
| **Hand-foot-and-mouth disease** | | | | | | |
| Intercept | | -3·393 | | 0·428 | -7·936 | <0·001 |
| Incidence rate (O) | | 0·002 | | 0·0001 | 13·692 | <0·001 |
| Human mobility intensity | | 0·690 | | 0·014 | 50·673 | <0·001 |
| Labor flow intensity | | 0·650 | | 0·023 | 28·428 | <0·001 |
| Geographic distance | | 0·097 | | 0·036 | 2·695 | 0·007 |
| Geographic adjacency | | 1·622 | | 0·106 | 15·241 | <0·001 |
| Intra-province | | 0·172 | | 0·067 | 2·572 | 0·010 |
| GRP per capita (O) | | -0·009 | | 0·006 | -1·602 | 0·109 |
| Provincial capital (O) | | 0·336 | | 0·040 | 8·326 | <0·001 |
| Industrial structure (O) | | -0·008 | | 0·002 | -3·386 | <0·001 |
| Provincial capital (D) | | 1·096 | | 0·040 | 27·352 | <0·001 |
| Industrial structure (D) | | -0·007 | | 0·003 | -2·354 | 0·019 |
| GRP per capita (D) | | -0·017 | | 0·007 | -2·432 | 0·015 |
| **Syphilis** | | | | | | |
| Intercept | | | -2·431 | 0·297 | -8·185 | <0·001 |
| Incidence rate (O) | | | 0·005 | 0·0004 | 13·198 | <0·001 |
| Human mobility intensity | | | 0·566 | 0·010 | 59·083 | <0·001 |
| Labor flow intensity | | | 0·707 | 0·016 | 45·126 | <0·001 |
| Geographic distance | | | 0·165 | 0·025 | 6·608 | <0·001 |
| Geographic adjacency | | | 0·729 | 0·073 | 9·954 | <0·001 |
| Intra-province | | | 0·279 | 0·056 | 5·026 | <0·001 |
| GRP per capita (O) | | | -0·070 | 0·004 | -15·828 | <0·001 |
| Provincial capital (O) | | | 0·430 | 0·032 | 13·464 | <0·001 |
| Industrial structure (O) | | | -0·029 | 0·001 | -19·441 | <0·001 |
| Provincial capital (D) | | | 1·098 | 0·030 | 36·388 | <0·001 |
| Industrial structure (D) | | | 0·014 | 0·002 | 6·605 | <0·001 |
| GRP per capita (D) | | | 0·041 | 0·004 | 10·158 | <0·001 |
| **Influenza** | | | | | | |
| Intercept | | | -8·652 | 0·523 | -16·547 | <0·001 |
| Incidence rate (O) | | | 0·001 | 0·0002 | 8·957 | <0·001 |
| Human mobility intensity | | | 0·704 | 0·016 | 43·899 | <0·001 |
| Labor flow intensity | | | 0·565 | 0·022 | 25·230 | <0·001 |
| Geographic distance | | | 0·458 | 0·047 | 9·681 | <0·001 |
| Geographic adjacency | | | 1·375 | 0·141 | 9·783 | <0·001 |
| Intra-province | | | 0·443 | 0·084 | 5·253 | <0·001 |
| GRP per capita (O) | | | 0·024 | 0·006 | 3·967 | <0·001 |
| Provincial capital (O) | | | 0·523 | 0·040 | 12·941 | <0·001 |
| Industrial structure (O) | | | -0·016 | 0·002 | -6·487 | <0·001 |
| Provincial capital (D) | | | 0·858 | 0·040 | 21·556 | <0·001 |
| Industrial structure (D) | | | 0·027 | 0·004 | 7·672 | <0·001 |
| GRP per capita (D) | | | 0·041 | 0·006 | 6·625 | <0·001 |
| **Hepatitis C** | | | | | | |
| Intercept | | | -3·891 | 0·436 | -8·914 | <0·001 |
| Incidence rate (O) | | | 0·020 | 0·001 | 26·985 | <0·001 |
| Human mobility intensity | | | 0·569 | 0·016 | 35·165 | <0·001 |
| Labor flow intensity | | | 0·676 | 0·018 | 36·552 | <0·001 |
| Geographic distance | | | 0·283 | 0·043 | 6·592 | <0·001 |
| Geographic adjacency | | | 0·869 | 0·089 | 9·743 | <0·001 |
| Intra-province | | | 0·588 | 0·075 | 7·815 | <0·001 |
| GRP per capita (O) | | | -0·081 | 0·008 | -10·202 | <0·001 |
| Provincial capital (O) | | | 0·332 | 0·048 | 6·866 | <0·001 |
| Industrial structure (O) | | | -0·028 | 0·002 | -13·668 | <0·001 |
| Provincial capital (D) | | | 1·255 | 0·043 | 29·293 | <0·001 |
| Industrial structure (D) | | | 0·007 | 0·002 | 3·004 | 0·003 |
| GRP per capita (D) | | | 0·014 | 0·006 | 2·467 | 0·014 |
| **HIV/AIDS** | | | | | | |
| Intercept | -9·388 | | | 0·331 | -28·392 | <0·001 |
| Incidence rate (O) | 0·036 | | | 0·0004 | 90·177 | <0·001 |
| Human mobility intensity | 0·713 | | | 0·011 | 63·516 | <0·001 |
| Labor flow intensity | 0·661 | | | 0·014 | 45·902 | <0·001 |
| Geographic distance | 0·734 | | | 0·028 | 26·458 | <0·001 |
| Geographic adjacency | 0·305 | | | 0·076 | 4·020 | <0·001 |
| Intra-province | 0·331 | | | 0·054 | 6·174 | <0·001 |
| GRP per capita (O) | -0·024 | | | 0·005 | -5·085 | <0·001 |
| Provincial capital (O) | 0·365 | | | 0·032 | 11·526 | <0·001 |
| Industrial structure (O) | -0·005 | | | 0·002 | -2·341 | 0·019 |
| Provincial capital (D) | 0·703 | | | 0·029 | 24·479 | <0·001 |
| Industrial structure (D) | 0·006 | | | 0·002 | 3·252 | 0·001 |
| GRP per capita (D) | 0·049 | | | 0·004 | 13·231 | <0·001 |
| **Gonorrhea** | | | | | | |
| Intercept | -6·764 | | | 0·434 | -15·571 | <0·001 |
| Incidence rate (O) | 0·001 | | | 0·001 | 0·778 | 0·437 |
| Human mobility intensity | 0·614 | | | 0·012 | 51·791 | <0·001 |
| Labor flow intensity | 0·816 | | | 0·019 | 43·518 | <0·001 |
| Geographic distance | 0·476 | | | 0·033 | 14·235 | <0·001 |
| Geographic adjacency | 0·067 | | | 0·076 | 0·881 | 0·379 |
| Intra-province | 0·138 | | | 0·057 | 2·434 | 0·015 |
| GRP per capita (O) | -0·059 | | | 0·005 | -11·058 | <0·001 |
| Provincial capital (O) | 0·162 | | | 0·042 | 3·839 | <0·001 |
| Industrial structure (O) | -0·011 | | | 0·002 | -5·938 | <0·001 |
| Provincial capital (D) | 0·670 | | | 0·037 | 18·107 | <0·001 |
| Industrial structure (D) | 0·011 | | | 0·003 | 3·906 | <0·001 |
| GRP per capita (D) | 0·046 | | | 0·004 | 10·459 | <0·001 |
| **Brucellosis** | | | | | | |
| Intercept | 5·802 | | | 1·965 | 2·952 | 0·003 |
| Incidence rate (O) | 0·038 | | | 0·002 | 17·404 | <0·001 |
| Human mobility intensity | 0·773 | | | 0·112 | 6·924 | <0·001 |
| Labor flow intensity | 0·269 | | | 0·067 | 3·989 | <0·001 |
| Geographic distance | -0·061 | | | 0·223 | -0·273 | 0·785 |
| Geographic adjacency | 1·395 | | | 0·211 | 6·625 | <0·001 |
| Intra-province | 0·477 | | | 0·370 | 1·289 | 0·197 |
| GRP per capita (O) | -0·069 | | | 0·039 | -1·759 | 0·079 |
| Provincial capital (O) | 0·045 | | | 0·225 | 0·202 | 0·840 |
| Industrial structure (O) | -0·045 | | | 0·012 | -3·871 | <0·001 |
| Provincial capital (D) | 2·339 | | | 0·245 | 9·546 | <0·001 |
| Industrial structure (D) | -0·095 | | | 0·008 | -11·909 | <0·001 |
| GRP per capita (D) | 0·089 | | | 0·020 | 4·424 | <0·001 |
| **Mumps** | | | | | | |
| Intercept | -5·136 | | | 0·660 | -7·786 | <0·001 |
| Incidence rate (O) | 0·018 | | | 0·001 | 12·426 | <0·001 |
| Human mobility intensity | 0·660 | | | 0·024 | 27·152 | <0·001 |
| Labor flow intensity | 0·386 | | | 0·027 | 14·074 | <0·001 |
| Geographic distance | 0·252 | | | 0·061 | 4·127 | <0·001 |
| Geographic adjacency | 1·316 | | | 0·120 | 10·964 | <0·001 |
| Intra-province | 0·525 | | | 0·098 | 5·357 | <0·001 |
| GRP per capita (O) | -0·044 | | | 0·010 | -4·181 | <0·001 |
| Provincial capital (O) | 0·430 | | | 0·062 | 6·982 | <0·001 |
| Industrial structure (O) | -0·020 | | | 0·005 | -3·875 | <0·001 |
| Provincial capital (D) | 1·185 | | | 0·066 | 18·071 | <0·001 |
| Industrial structure (D) | -0·004 | | | 0·004 | -1·009 | 0·313 |
| GRP per capita (D) | -0·017 | | | 0·008 | -2·105 | 0·035 |
| **Hepatitis E** | | | | | | |
| Intercept | -4·692 | | | 0·863 | -5·435 | <0·001 |
| Incidence rate (O) | 0·053 | | | 0·015 | 3·452 | <0·001 |
| Human mobility intensity | 0·530 | | | 0·033 | 16·054 | <0·001 |
| Labor flow intensity | 0·707 | | | 0·039 | 18·267 | <0·001 |
| Geographic distance | -0·005 | | | 0·078 | -0·068 | 0·946 |
| Geographic adjacency | 0·837 | | | 0·137 | 6·105 | <0·001 |
| Intra-province | 0·295 | | | 0·174 | 1·699 | 0·089 |
| GRP per capita (O) | -0·084 | | | 0·013 | -6·574 | <0·001 |
| Provincial capital (O) | 0·295 | | | 0·098 | 3·026 | 0·002 |
| Industrial structure (O) | -0·037 | | | 0·005 | -6·780 | <0·001 |
| Provincial capital (D) | 1·767 | | | 0·085 | 20·714 | <0·001 |
| Industrial structure (D) | 0·024 | | | 0·006 | 3·923 | <0·001 |
| GRP per capita (D) | 0·011 | | | 0·011 | 1·050 | 0·294 |
| **Pertussis** | | | | | | |
| Intercept | -10·509 | | | 1·849 | -5·684 | <0·001 |
| Incidence rate (O) | 0·210 | | | 0·027 | 7·863 | <0·001 |
| Human mobility intensity | 0·919 | | | 0·076 | 12·167 | <0·001 |
| Labor flow intensity | 0·551 | | | 0·062 | 8·912 | <0·001 |
| Geographic distance | 0·028 | | | 0·241 | 0·118 | 0·906 |
| Geographic adjacency | 1·640 | | | 0·307 | 5·350 | <0·001 |
| Intra-province | -0·449 | | | 0·279 | -1·610 | 0·107 |
| GRP per capita (O) | -0·069 | | | 0·041 | -1·689 | 0·091 |
| Provincial capital (O) | -0·372 | | | 0·226 | -1·647 | 0·100 |
| Industrial structure (O) | -0·044 | | | 0·010 | -4·574 | <0·001 |
| Provincial capital (D) | 2·526 | | | 0·227 | 11·124 | <0·001 |
| Industrial structure (D) | 0·059 | | | 0·015 | 4·003 | <0·001 |
| GRP per capita (D) | -0·057 | | | 0·023 | -2·482 | 0·013 |
| **Shigellosis** | | | | | | |
| Intercept | -6·425 | | | 1·203 | -5·339 | <0·001 |
| Incidence rate (O) | 0·025 | | | 0·003 | 8·522 | <0·001 |
| Human mobility intensity | 0·566 | | | 0·032 | 17·599 | <0·001 |
| Labor flow intensity | 0·377 | | | 0·036 | 10·481 | <0·001 |
| Geographic distance | 0·415 | | | 0·098 | 4·231 | <0·001 |
| Geographic adjacency | 1·954 | | | 0·242 | 8·078 | <0·001 |
| Intra-province | 0·732 | | | 0·161 | 4·544 | <0·001 |
| GRP per capita (O) | 0·006 | | | 0·015 | 0·408 | 0·683 |
| Provincial capital (O) | 0·445 | | | 0·078 | 5·737 | <0·001 |
| Industrial structure (O) | -0·022 | | | 0·005 | -4·484 | <0·001 |
| Provincial capital (D) | 1·447 | | | 0·089 | 16·194 | <0·001 |
| Industrial structure (D) | -0·003 | | | 0·007 | -0·364 | 0·716 |
| GRP per capita (D) | -0·039 | | | 0·014 | -2·891 | 0·004 |
| **Hepatitis E** | | | | | | |
| Intercept | -6·366 | | | 1·051 | -6·054 | <0·001 |
| Incidence rate (O) | 0·094 | | | 0·007 | 12·618 | <0·001 |
| Human mobility intensity | 0·655 | | | 0·045 | 14·709 | <0·001 |
| Labor flow intensity | 0·585 | | | 0·040 | 14·583 | <0·001 |
| Geographic distance | 0·508 | | | 0·113 | 4·512 | <0·001 |
| Geographic adjacency | 1·036 | | | 0·156 | 6·639 | <0·001 |
| Intra-province | 0·704 | | | 0·167 | 4·220 | <0·001 |
| GRP per capita (O) | -0·082 | | | 0·020 | -4·062 | <0·001 |
| Provincial capital (O) | 0·201 | | | 0·127 | 1·589 | 0·112 |
| Industrial structure (O) | -0·034 | | | 0·007 | -4·828 | <0·001 |
| Provincial capital (D) | 1·730 | | | 0·098 | 17·609 | <0·001 |
| Industrial structure (D) | -0·001 | | | 0·006 | -0·218 | 0·827 |
| GRP per capita (D) | -0·069 | | | 0·014 | -5·005 | <0·001 |

**Table S3. Summary of the 44 types of notifiable infectious diseases in mainland China during 2016–2020.**

| **Disease** | **Abbreviation** | **Diagnostic criteria^a^** | **Number of incident cases** | **Annual incidence**  **(per 100,000)** |
| --- | --- | --- | --- | --- |
| **Respiratory transmitted diseases** | | | | |
| Influenza |  | WS285-2008 | 6 230 343 | 88·44 |
| Tuberculosis |  | WS288-2008 | 3 999 387 | 56·77 |
| Mumps |  | WS270-2007 | 1 117 231 | 15·85 |
| Scarlet fever |  | WS282-2008 | 311 635 | 4·42 |
| Pertussis |  | WS274-2007 | 72 710 | 1·03 |
| Rubella |  | WS297-2008 | 44 820 | 0·63 |
| Measles |  | WS296-2008 | 38 999 | 0·55 |
| Epidemic cerebrospinal meningitis | ECM | WS295-2008 | 489 | <0·01 |
| Diphtheria |  | WS275-2007 | 2 | <0·01 |
| Severe acute respiratory syndrome | SARS | WS286-2008 | 0 | 0 |
| **Direct-contact or fecal-oral transmitted diseases** | | | | |
| Hand-foot-and-mouth disease | HFMD | WS588-2018 | 9 409 017 | 133·56 |
| Shigellosis |  | WS287-2008 | 458 685 | 6·51 |
| Acute hemorrhagic conjunctivitis | AHC | WS217-2001 | 177 229 | 2·51 |
| Hepatitis E |  | WS301-2008 | 133 935 | 1·90 |
| Hepatitis A |  | WS298-2008 | 91 265 | 1·29 |
| Typhoid |  | WS280-2008 | 37 969 | 0·53 |
| Paratyphoid |  | WS280-2008 | 11 062 | 0·16 |
| Amoebic dysentery |  | WS287-2008 | 4690 | 0·06 |
| Leprosy |  | WS291-2018 | 2201 | 0·03 |
| Neonatal tetanus |  | WS272-2007 | 457 | <0·01 |
| Cholera |  | WS289-2008 | 96 | <0·01 |
| Poliomyelitis |  | WS294-2016 | 0 | 0 |
| **Blood and sexually transmitted diseases** | | | | |
| Hepatitis B |  | WS299-2008 | 5 136 056 | 72·90 |
| Syphilis |  | WS273-2018 | 2 447 645 | 34·74 |
| Hepatitis C |  | WS213-2008 | 1 109 374 | 15·74 |
| Human immunodeficiency virus infection / Acquired immune deficiency syndrome | HIV/  AIDS | WS293-2008 | 803 553 | 11·40 |
| Gonorrhea |  | WS296-2008 | 612 682 | 8·69 |
| Hepatitis D |  | WS300-2008 | 1734 | 0·02 |
| **Zoonotic or vector-borne diseases** | | | | |
| Brucellosis |  | WS269-2007 | 220 077 | 3·12 |
| Hemorrhagic fever with renal syndrome | HFRS | WS278-2008 | 49 844 | 0·70 |
| Dengue |  | WS216-2018 | 37 993 | 0·53 |
| Hydatid diseases |  | WS257-2006 | 23 578 | 0·33 |
| Malaria |  | WS259-2006 | 12 632 | 0·17 |
| Schistosomiasis |  | WS261-2006 | 6958 | 0·09 |
| Typhus |  | WS215-2001 | 5329 | 0·07 |
| Japanese encephalitis |  | WS214-2008 | 4926 | 0·06 |
| Rabies |  | WS281-2008 | 2084 | 0·02 |
| Anthrax |  | WS283-2008 | 1549 | 0·02 |
| Leptospirosis |  | WS290-2008 | 1225 | 0·01 |
| Kala-azar |  | WS258-2006 | 1076 | 0·01 |
| Avian flu H7N9 | H7N9 | DTP H7N9 | 864 | 0·01 |
| Plague |  | WS279-2008 | 12 | <0·01 |
| Avian flu H5N1 | H5N1 | WS284-2008 | 1 | <0·01 |
| Filariasis |  | WS260-2006 | 0 | 0 |
| **Total** |  | – | 32 621 414 | 463·07 |

^a^ These diagnostic criteria of notifiable infectious diseases were available from the websites of China CDC (https://icdc.chinacdc.cn/zcfgybz/bz/index_1.html &. https://www.chinacdc.cn/jkzt/crb/zl/rgrgzbxqlgg/rgrglgyh/).

**Table S4. Gender and age distribution of local and migratory cases for 39 notifiable infectious diseases from 2016 to 2020.**

| **Disease** |  | **Male (N, %)** | |  |  | **Age (Median, IQR)** | |
| --- | --- | --- | --- | --- | --- | --- | --- |
|  |  | **Local cases** | **Migrate cases** | |  | **Local cases** | **Migrate cases** |
| AHC |  | 92 752 (53·05%) | 1422 (59·35%) | |  | 37·49 (15·85, 55·08) | 30·26 (19·90, 43·64) |
| Amobiec dysentery | | 2392 (56.68%) | 282 (60·00%) | |  | 8·68 (1·35, 49·58) | 39·26 (23·47, 54·63) |
| Anthrax |  | 984 (72·30%) | 151 (80·32%) | |  | 42·02 (31·82, 51·12) | 44·29 (35·28, 52·17) |
| Brucellosis | | 129 641 (71.70 %) | 28 543 (72·68%) | |  | 49·76 (39·82, 58·29) | 48·19 (36·27, 56·77) |
| Cholera |  | 46 (50·00%) | 4 (100·00%) | |  | 36·08 (27·48, 53·68) | 40·97 (34·77, 46·41) |
| Dengue |  | 18 782 (55·66%) | 2429 (57·14%) | |  | 39·77 (28·46, 53·01) | 31·89 (23·74, 44·44) |
| ECM |  | 251 (68·21%) | 78 (64·46%) | |  | 11·38 (2·12, 16·28) | 8·00 (1·00, 15·15) |
| Gonorrhea | | 448 157 (83.81%) | 66 337 (85·12%) | |  | 30·00 (24·00, 39·88) | 28·38 (23·14, 35·71) |
| H7N9 |  | 495 (70·92%) | 116 (69·88%) | |  | 58·33 (47·00, 67·19) | 53·81 (41·13, 63·16) |
| Hepatitis A | | 45 401 (56.49%) | 6213 (57·04%) | |  | 47·57 (33·36, 61·14) | 46·66 (31·84, 58·25) |
| Hepatitis B | | 2 740 365 (62.79%) | 527 966 (68·44%) | |  | 46·47 (33·48, 57·99) | 44·11 (32·18, 54·47) |
| Hepatitis C | | 546 005 (56.61%) | 83 952 (57·92%) | |  | 52·87 (43·30, 64·18) | 50·53 (41·40, 60·41) |
| Hepatitis D | | 936 (60.54%) | 122 (64·89%) | |  | 49·20 (35·70, 60·48) | 44·13 (32·87, 54·28) |
| Hepatitis E | | 72 242 (64.57%) | 15 437 (70·01%) | |  | 54·08 (44·00, 64·73) | 51·36 (41·00, 61·51) |
| HFMD |  | 5 366 705 (59·24%) | 212 245 (60·8%) | |  | 2·35 (1·43, 3·79) | 2·00 (1·28, 3·38) |
| HFRS |  | 29 040 (72·71%) | 7452 (75·25%) | |  | 50·31 (38·41, 60·96) | 47·81 (35·16, 57·00) |
| HIV/AIDS |  | 512 418 (77·11%) | 110 906 (79·79%) | |  | 46·21 (32·24, 59·65) | 37·89 (27·90, 49·98) |
| Hydatid disease | | 7042 (48.09%) | 4304 (48·18%) | |  | 45·25 (32·82, 57·33) | 42·94 (31·17, 53·60) |
| Influenza |  | 3 201 771 (53·26%) | 121 056 (55·34%) | |  | 7·64 (3·98, 21·72) | 6·98 (3·00, 26·64) |
| Japanese encephalitis | | 1561 (53.42%) | 1128 (56·29%) | |  | 46·34 (10·83, 63·70) | 29·64 (10·06, 54·52) |
| Kala-azar |  | 334 (61·97%) | 356 (66·29%) | |  | 7·76 (1·41, 48·31) | 26·29 (2·00, 46·51) |
| Leprosy |  | 1234 (68·67%) | 262 (64·85%) | |  | 43·53 (29·97, 55·3) | 45·07 (32·25, 54·70) |
| Leptospirosis | | 755 (70.89%) | 132 (82·50%) | |  | 54·29 (41·85, 64·82) | 53·72 (45·44, 61·66) |
| Malaria |  | 2 (100·00%) | 11 746 (93·00%) | |  | 19·00 (11·00, 27·00) | 40·50 (31·22, 48·00) |
| Measles |  | 17 924 (54·73%) | 3844 (61·50%) | |  | 6·16 (0·87, 30·29) | 1·15 (0·70, 9·13) |
| Mumps |  | 652 180 (59·81%) | 16 851 (63·02%) | |  | 8·71 (5·82, 12·12) | 9·38 (6·00, 15·92) |
| Neonatal tetanus | | 232 (62.87%) | 63 (71·59%) | |  | 0·02 (0·02, 0·03) | 0·03 (0·02, 0·04) |
| paratyphoid | | 5384 (53.31%) | 572 (59·40%) | |  | 25·28 (3·21, 52·05) | 26·34 (2·91, 47·29) |
| Pertussis |  | 28 289 (51·94%) | 9701 (53·18%) | |  | 0·78 (0·35, 2·74) | 0·60 (0·32, 1·60) |
| Plague |  | 4 (57·14%) | 3 (60·00%) |  |  | 47·31 (46·14, 59·53) | 46·24 (46·24, 55·94) |
| Rabies |  | 1130 (70·62%) | 338 (69·83%) | |  | 57·97 (44·21, 67·36) | 50·27 (30·01, 62·32) |
| Rubella |  | 25 178 (58·39%) | 1010 (59·34%) | |  | 16·65 (13·87, 21·33) | 18·98 (14·70, 24·91) |
| Scarlet fever | | 183 202 (60.59%) | 5744 (62·03%) | |  | 5·99 (4·68, 7·27) | 5·64 (4·38, 7·05) |
| Schistosomiasis | | 5063 (74.94%) | 140 (69·31%) | |  | 53·67 (46·95, 62·02) | 61·39 (50·25, 69·00) |
| Shigellosis | | 240 378 (53.77%) | 6526 (56·23%) | |  | 27·49 (2·82, 54·50) | 23·40 (2·70, 45·88) |
| Syphilis |  | 1 057 693 (49·5%) | 152 618 (49·12%) | |  | 48·76 (32·74, 64·59) | 42·69 (29·61, 55·84) |
| Tuberculosis | | 2 441 533 (69.16%) | 311 131 (66·31%) | |  | 51·54 (32·66, 65·31) | 44·00 (26·28, 59·55) |
| Typhoid |  | 17 887 (51·58%) | 1861 (56·55%) | |  | 32·25 (9·80, 53·69) | 28·66 (10·11, 48·89) |
| Typhus |  | 2370 (48·37%) | 232 (54·08%) | |  | 48·06 (23·58, 61·36) | 42·28 (15·44, 56·01) |

**Table S5. Top 3 occupation categories* associated with the highest number of local and migratory cases for 39 notifiable infectious diseases from 2016 to 2020.**

| **Disease** |  | **Local cases** | |  |  | **Migrate cases** | |  |
| --- | --- | --- | --- | --- | --- | --- | --- | --- |
|  |  | **Rank 1 (%)** | **Rank 2 (%)** | **Rank 3 (%)** |  | **Rank 1 (%)** | **Rank 2 (%)** | **Rank 3 (%)** |
| AHC |  | Farmers (50·78%) | Students (13·18%) | Children at home (7·68%) | | Farmers (28·63%) | Unemployed (14·11%) | Children at home (11·02%) |
| Amobiec dysentery | | Children at home (42.75%) | Farmers (23·93%) | Unemployed (7·91%) | | Farmers (35·32%) | Unemployed (22·34%) | Children at home (15·11%) |
| Anthrax |  | Herders (53·71%) | Farmers (36·66%) | Students (2·72%) | | Farmers (48·4%) | Herders (31·91%) | Unemployed (9·04%) |
| Brucellosis | | Farmers (80.09%) | Herders (5·58%) | Unemployed (3·83%) | | Farmers (72·76%) | Unemployed (7·45%) | Herders (3·96%) |
| Cholera |  | Unemployed (19·57%) | Goverment employees (18·48%) | Farmers (18·48%) | | Goverment employees (50·00%) | Farmers (25·00%) | Migrant workers (25·00%) |
| Dengue |  | Farmers (17·76%) | Unemployed (17·73%) | Businessmen (14·59%) | | Farmers (25·45%) | Businessmen (16·04%) | Unemployed (14·73%) |
| ECM |  | Students (42·12%) | Children at home (33·42%) | Farmers (6·79%) | | Children at home (43·80%) | Students (35·54%) | Farmers (8·26%) |
| Gonorrhea | | Farmers (28.47%) | Unemployed (24·32%) | Businessmen (15·91%) | | Farmers (25·22%) | Unemployed (25·14%) | Businessmen (20·67%) |
| H7N9 |  | Farmers (41·4%) | Retirees (18·62%) | Unemployed (11·75%) | | Farmers (40·96%) | Unemployed (15·66%) | Retirees (11·45%) |
| Hepatitis A | | Farmers (44.62%) | Unemployed (15·2%) | Retirees (9·04%) | | Farmers (38·92%) | Unemployed (18·66%) | Retirees (7·26%) |
| Hepatitis B | | Farmers (59.05%) | Unemployed (14·38%) | Retirees (4·93%) | | Farmers (42·44%) | Unemployed (20·11%) | Unknown (9·04%) |
| Hepatitis C | | Farmers (56.32%) | Unemployed (16·7%) | Retirees (8·85%) | | Farmers (45·96%) | Unemployed (20·96%) | Unknown (7·39%) |
| Hepatitis D | | Farmers (59.57%) | Unemployed (12·35%) | Retirees (6·27%) | | Farmers (38·30%) | Unemployed (21·28%) | Businessmen (9·57%) |
| Hepatitis E | | Farmers (48.35%) | Unemployed (15·35%) | Retirees (12·27%) | | Farmers (44·55%) | Unemployed (19·43%) | Unknown (8·00%) |
| HFMD |  | Children at home (69·35%) | Children in nursery (26·09%) | Students (3·98%) | | Children at home (78·01%) | Children in nursery (18·4%) | Students (2·87%) |
| HFRS |  | Farmers (69·07%) | Unemployed (8·77%) | Industrial workers (4·65%) | | Farmers (60·92%) | Unemployed (14·23%) | Students (4·40%) |
| HIV/AIDS |  | Farmers (52·19%) | Unemployed (17·61%) | Businessmen (6·93%) | | Farmers (31·26%) | Unemployed (25·49%) | Businessmen (10·68%) |
| Hydatid disease | | Farmers (52.15%) | Herders (20·68%) | Unemployed (7·16%) | | Herders (37·89%) | Farmers (31·17%) | Unemployed (9·06%) |
| Influenza |  | Students (32·42%) | Children at home (23·59%) | Children in nursery (18·27%) | | Children at home (33·88%) | Students (19·36%) | Children in nursery (14·67%) |
| Japanese encephalitis | | Farmers (50.41%) | Students (19·34%) | Children at home (12·56%) | | Farmers (42·66%) | Students (24·85%) | Children at home (11·83%) |
| Kala-azar |  | Children at home (45·64%) | Farmers (31·35%) | Students (6·12%) | | Children at home (37·62%) | Farmers (31·47%) | Unemployed (7·08%) |
| Leprosy |  | Farmers (74·12%) | Unemployed (6·84%) | Students (6·18%) | | Farmers (53·96%) | Unemployed (12·13%) | Businessmen (8·42%) |
| Leptospirosis | | Farmers (78.03%) | Unemployed (6·20%) | Retirees (2·63%) | | Farmers (51·25%) | Unemployed (15·00%) | Businessmen (5·62%) |
| Malaria |  | Children at home (50·00%) | Unknown (50·00%) | - |  | Farmers (36·40%) | Industrial workers (16·84%) | Unemployed (10.08%) |
| Measles |  | Children at home (47·62%) | Farmers (17·12%) | Students (7·94%) | | Children at home (69·87%) | Farmers (10·51%) | Students (5·44%) |
| Mumps |  | Students (58·17%) | Children in nursery (20·37%) | Children at home (11·44%) | | Students (49·13%) | Children at home (16·29%) | Children in nursery (15·37%) |
| Neonatal tetanus | | Children at home (100.00%) | - | - |  | Children at home (100·00%) | - | - |
| paratyphoid | | Farmers (30.66%) | Children at home (28·01%) | Students (13·74%) | | Children at home (28·45%) | Farmers (24·92%) | Unemployed (11·32%) |
| Pertussis |  | Children at home (83·49%) | Children in nursery (9·91%) | Students (5·07%) | | Children at home (88·18%) | Children in nursery (7·58%) | Students (3·61%) |
| Plague |  | Herders (42·86%) | Farmers (14·29%) | Others (14·29%) | | Herders (60·00%) | Farmers (20·00%) | Industrial workers (20·00%) |
| Rabies |  | Farmers (75·62%) | Students (7·12%) | Children at home (4·50%) | | Farmers (62·81%) | Students (9·71%) | Unemployed (9·50%) |
| Rubella |  | Students (63·65%) | Industrial workers (8·24%) | Unemployed (6·27%) | | Students (41·89%) | Children at home (13·75%) | Unemployed (11·69%) |
| Scarlet fever | | Children in nursery (44.99%) | Students (37·60%) | Children at home (16·59%) | | Children in nursery (41·91%) | Students (30·09%) | Children at home (25·91%) |
| Schistosomiasis | | Farmers (86.55%) | Fisherman (7·27%) | Retirees (2·12%) | | Farmers (64·36%) | Unemployed (15·35%) | Retirees (8·42%) |
| Shigellosis | | Farmers (30.49%) | Children at home (29·3%) | Students (10·27%) | | Children at home (31·26%) | Farmers (21·55%) | Unemployed (10·92%) |
| Syphilis |  | Farmers (46·04%) | Unemployed (23·68%) | Retirees (8·16%) | | Farmers (32·9%) | Unemployed (29·33%) | Unknown (9·49%) |
| Tuberculosis | | Farmers (63.17%) | Unemployed (13·10%) | Retirees (5·03%) | | Farmers (51·48%) | Unemployed (18·23%) | Students (8·68%) |
| Typhoid |  | Farmers (39·02%) | Children at home (17·39%) | Students (12·52%) | | Farmers (29·05%) | Children at home (18·99%) | Unemployed (14·13%) |
| Typhus |  | Farmers (62·33%) | Students (12·33%) | Children at home (7·29%) | | Farmers (52·91%) | Students (16·32%) | Children at home (7·69%) |

*A total of 20 occupational categories were reported for all cases, including business persons, government employees, catering persons, farmers, fisherman, herders, industrial workers, medical faculty, migrant workers, children in nursery, nurses & nannies, public place workers, retirees, sailors and long-distant drivers, children at home, students, teachers, unemployed, others not falling into any specific category mentioned above and those whose occupation is unknown.

**Table S6. Temporal trend of the annual proportion of migratory cases for 14 notifiable infectious diseases from 2016 to 2019.** Results presented are the coefficients of year obtained from linear regressions, with the annual proportions as the response variable and year as the predictor.

| **Disease** | ***β*** | **Standard error** | ***t* value** | ***p* value** |
| --- | --- | --- | --- | --- |
| Hepatitis B | 0·570 | 0·163 | 3·495 | 0·073 |
| Tuberculosis | 1·071 | 0·065 | 16·381 | 0·004 |
| HFMD | -0·039 | 0·054 | -0·713 | 0·550 |
| Syphilis | 0·375 | 0·070 | 5·378 | 0·033 |
| Influenza | 0·153 | 0·166 | 0·917 | 0·456 |
| Hepatitis C | -0·006 | 0·057 | -0·102 | 0·928 |
| HIV/AIDS | -1·443 | 0·245 | -5·899 | 0·028 |
| Gonorrhea | 1·395 | 0·135 | 10·372 | 0·009 |
| Brucellosis | 0·008 | 0·219 | 0·036 | 0·974 |
| Mumps | 0·076 | 0·038 | 1·988 | 0·185 |
| Hepatitis E | 0·213 | 0·190 | 1·118 | 0·380 |
| Pertussis | -2·408 | 0·515 | -4·674 | 0·043 |
| Shigellosis | 0·206 | 0·068 | 3·035 | 0·094 |
| Hepatitis A | 1·377 | 0·158 | 8·711 | 0·013 |

**Table S7. Duration from symptom onset to diagnosis of local and migratory cases for 14 major notifiable infectious diseases in China during 2016–2020.** Median delays that are ≥2 days longer than the other case group are colored in red.

| **Disease** | **Local cases (day)** | |  | **Migratory cases (day)** | | ***p*-value^a^** |
| --- | --- | --- | --- | --- | --- | --- |
|  | **Median (IQR)** | **Mean (SD)** |  | **Median (IQR)** | **Mean (SD)** |  |
| Hepatitis B | 5 (1, 21) | 64·51 (195·16) |  | 6 (2, 26) | 66·26 (196·52) | <0·001 |
| Tuberculosis | 23 (9, 49) | 46·07 (86·24) |  | 20 (7, 47) | 45·70 (88·82) | <0·001 |
| Hand, foot and mouth disease | 2 (1, 3) | 2·78 (9·51) |  | 2 (1, 3) | 3·20 (11·99) | <0·001 |
| Syphilis | 3 (1, 10) | 22·81 (89·89) |  | 4 (1, 12) | 24·29 (90·74) | <0·001 |
| Influenza | 2 (1, 3) | 3·38 (12·18) |  | 2 (1, 4) | 4·38 (13·90) | <0·001 |
| Hepatitis C | 4 (1, 14) | 46·51 (150·63) |  | 6 (2, 24) | 67·05 (183·79) | <0·001 |
| HIV/AIDS | 14 (7, 36) | 87·52 (231·04) |  | 12 (6, 33) | 96·91 (258·29) | <0·001 |
| Gonorrhea | 4 (2, 7) | 8·88 (26·27) |  | 4 (2, 8) | 9·79 (31·41) | 0·874 |
| Brucellosis | 15 (6, 33) | 34·11 (74·41) |  | 13 (4, 32) | 34·23 (75·27) | <0·001 |
| Mumps | 2 (1, 3) | 3·35 (13·62) |  | 2 (1, 5) | 4·61 (14·46) | <0·001 |
| Hepatitis E | 7 (3, 14) | 15·41 (47·28) |  | 7 (3, 15) | 16·54 (53·24) | 0·001 |
| Pertussis | 13 (7, 21) | 17·39 (21·44) |  | 16 (9, 24) | 19·31 (19·00) | <0·001 |
| Shigellosis | 2 (1, 3) | 3·73 (12·82) |  | 2 (1, 4) | 4·97 (13·60) | <0·001 |
| Hepatitis A | 5 (2, 10) | 12·69 (45·50) |  | 5 (2, 11) | 13·11 (44·44) | 0·915 |

^a^*p-*values were based on two-sample Wilcoxon rank sum test.

**Table S8. Comparison of case fatality ratios (CFR) between local cases and migratory cases for 14 notifiable infectious diseases in China during 2016‒2020.** CFRs that are ≥0.2‰ higher than the other case group are colored in red.

| **Disease** | **Local Cases** | |  |  | **Migratory Cases** | | ***p*-value^a^** | |
| --- | --- | --- | --- | --- | --- | --- | --- | --- |
|  | **Crude CFR (‰)** | **Death/Total cases** |  | **Crude CFR (‰)** | | **Death/Total cases** |  |  |
| Hepatitis B | 0·337 | 1 469/4 364 633 |  | 0·367 | | 283/771 423 | 0·192 |  |
| Tuberculosis | 3·236 | 11 424/3 530 183 |  | 1·652 | | 775/469 204 | <0·001 |  |
| Hand, foot and mouth disease | 0·028 | 252/9 059 925 |  | 0·163 | | 57/349 092 | <0·001 |  |
| Syphilis | 0·079 | 169/2 136 930 |  | 0·077 | | 24/310 715 | 1·0 |  |
| Influenza | 0·085 | 512/6 011 594 |  | 0·306 | | 67/218 749 | <0·001 |  |
| Hepatitis C | 0·407 | 393/964 429 |  | 0·407 | | 59/144 945 | 1·0 |  |
| HIV/AIDS | 66·044 | 43 890/664 558 |  | 62·268 | | 8 655/138 995 | <0·001 |  |
| Gonorrhea | 0·004 | 2/534 744 |  | 0·013 | | 1/77 938 | 0·335 |  |
| Brucellosis | 0·017 | 3/180 806 |  | 0·051 | | 2/39 271 | 0·219 |  |
| Mumps | 0·001 | 1/1 090 492 |  | 0 | | 0/26 739 | 1·0 |  |
| Hepatitis E | 0·572 | 64/111 886 |  | 0·590 | | 13/22 049 | 0·878 |  |
| Pertussis | 0·055 | 3/54 467 |  | 0·274 | | 5/18 243 | 0·028 |  |
| Shigellosis | 0·013 | 6/447 079 |  | 0·258 | | 3/11 606 | 0·0012 |  |
| Hepatitis A | 0·187 | 15/80 372 |  | 0 | | 0/10 893 | 0·243 |  |

^a^*p* values were based on Fisher’s exact test.

**Table S9.** **Duration (days) from symptom onset to diagnosis between groups of migratory and local cases for 14 major notifiable infectious diseases in China during 2016–2020. The groups are based on provincial capital cities (capital cities) vs. other Chinese cities (non-capital cities).** Median delays that are ≥2 days longer than at least two other groups are colored in red.

| **Disease** | **Migratory cases**  **from noncapital cities**  **to capital cities** | | **Other migratory cases** | | **Local cases in**  **provincial capital cities** | | **Local cases in**  **general cities** | | ***p-*value^$^** |
| --- | --- | --- | --- | --- | --- | --- | --- | --- | --- |
|  | Median (IQR) | Mean (SD) | Median (IQR) | Mean (SD) | Median (IQR) | Mean (SD) | Median (IQR) | Mean (SD) |  |
| Hepatitis B | 7 (2‒24) | 61·66 (190·18) | 5 (2‒30) | 76·01 (208·97) | 7 (2‒32) | 83·58 (246·36) | 4 (1‒19) | 60·40 (181·98) | <0·001 |
| Tuberculosis | 20 (7‒48) | 45·73 (88·08) | 20 (7‒47) | 45·65 (90·54) | 21 (8‒47) | 45·63 (91·78) | 23 (9‒49) | 46·17 (84·99) | <0·001 |
| HFMD | 2 (1‒3) | 3·47 (13·60) | 2 (1‒3) | 2·86 (9·58) | 2 (1‒3) | 2·68 (9·44) | 2 (1‒3) | 2·82 (9·53) | <0·001 |
| Syphilis | 4 (1‒12) | 23·34 (87·66) | 3 (1‒12) | 25·83 (95·54) | 3 (1‒11) | 22·90 (92·44) | 3 (1‒9) | 22·78 (88·96) | <0·001 |
| Influenza | 2 (1‒4) | 4·38 (14·37) | 2 (1‒4) | 4·38 (13·19) | 2 (1‒3) | 2·93 (10·64) | 2 (1‒3) | 3·66 (13·03) | <0·001 |
| Hepatitis C | 7 (2‒27) | 72·09 (192·25) | 5 (2‒18) | 56·98 (165·12) | 5 (2‒19) | 54·93 (170·48) | 4 (1‒13) | 44·34 (145·01) | <0·001 |
| HIV/AIDS | 12 (7‒35) | 89·85 (241·92) | 11 (6‒31) | 103·91 (273·35) | 13 (7‒28) | 69·55 (202·12) | 14 (7‒40) | 94·70 (241·24) | <0·001 |
| Gonorrhea | 4 (2‒7) | 10·09 (35·07) | 4 (2‒8) | 9·52 (27·73) | 4 (2‒7) | 8·45 (24·53) | 4 (2‒7) | 9·06 (26·95) | <0·001 |
| Brucellosis | 11 (3‒31) | 31·00 (65·04) | 16 (6‒34) | 39·24 (88·60) | 15 (6‒35) | 34·46 (70·87) | 15 (6‒32) | 34·08 (74·76) | <0·001 |
| Mumps | 3 (1‒5) | 4·79 (14·46) | 2 (1‒4) | 4·41 (14·47) | 1 (1‒3) | 2·89 (11·01) | 2 (1‒3) | 3·49 (14·30) | <0·001 |
| Hepatitis E | 7 (3‒16) | 15·61 (42·91) | 7 (2‒15) | 19·45 (77·15) | 7 (3‒14) | 15·51 (45·50) | 7 (3‒14) | 15·36 (48·02) | <0·001 |
| Pertussis | 16 (10‒25) | 19·58 (18·79) | 14 (7‒22) | 17·59 (20·24) | 15 (8‒22) | 17·77 (17·38) | 13 (7‒20) | 17·14 (23·72) | <0·001 |
| Shigellosis | 2 (1‒4) | 4·80 (11·22) | 2 (1‒4) | 5·11 (15·27) | 2 (1‒3) | 3·36 (10·62) | 2 (1‒3) | 3·97 (14·06) | <0·001 |
| Hepatitis A | 5 (2‒11) | 12·76 (43·34) | 5 (2‒12) | 14·00 (47·11) | 5 (2‒11) | 13·74 (47·03) | 5 (2‒10) | 12·37 (45·03) | <0·001 |

$ *p-*values were based on Kruskal Walis test, which are all significant due to large sample size.

**Table S10. Comparison of case fatality ratios (CFR, ‰) between groups of migratory and local cases for 14 notifiable infectious diseases in China during 2016‒2020. The groups are based on provincial capital cities (capital cities) vs. other Chinese cities (non-capital cities).** CFRs that are ≥0.2‰ higher than at least two other groups are colored in red.

| **Disease** | **Migratory cases from**  **noncapital to capital cities** | **Other migratory cases** | **Local cases in**  **capital cities** | **Local cases in**  **noncapital cities** | ***p-*value^$^** |
| --- | --- | --- | --- | --- | --- |
| Hepatitis B | 0·38 (198/520 968) | 0·34 (85/250 455) | 0·79 (595/750 583) | 0·24 (874/3 614 050) | <0·001 |
| Tuberculosis | 1·55 (511/329 190) | 1·89 (264/140 014) | 3·92 (2498/636 410) | 3·08 (8926/2 893 773) | <0·001 |
| HFMD | 0·15 (29/193 483) | 0·18 (28/155 609) | 0·019 (46/2 406 900) | 0·031 (206/6 653 025) | <0·001 |
| Syphilis | 0·059 (11/187 547) | 0·11 (13/123 168) | 0·086 (47/547 981) | 0·077 (122/1 588 949) | 0·440 |
| Influenza | 0·35 (45/128 963) | 0·25 (22/89 786) | 0·10 (221/2 206 236) | 0·076 (291/3 805 358) | <0·001 |
| Hepatitis C | 0·37 (36/96 885) | 0·48 (23/48 060) | 0·70 (137/194 465) | 0·33 (256/769 964) | <0·001 |
| HIV/AIDS | 73·09 (4986/68 217) | 51·84 (3669/70 778) | 53·47 (9643/180 358) | 70·73 (34 247/484 200) | <0·001 |
| Gonorrhea | 0·00 (0/35 837) | 0·024 (1/42 101) | 0·00 (0/153 061) | 0·0052 (2/381 683) | 0·346 |
| Brucellosis | 0·042 (1/23 736) | 0·064 (1/15 535) | 0·063 (1/15 925) | 0·012 (2/164 881) | 0·094 |
| Mumps | 0·00 (0/13 669) | 0·00 (0/13 070) | 0·00 (0/256 279) | 0·0012 (1/834 213) | 0·259 |
| Hepatitis E | 0·66 (11/16 644) | 0·37 (2/5405) | 1·36 (44/32 348) | 0·25 (20/79 538) | <0·001 |
| Pertussis | 0·19 (3/15 718) | 0·79 (2/2525) | 0·14 (3/20 708) | 0·00 (0/33 759) | 0·001 |
| Shigellosis | 0·19 (1/5 212) | 0·31 (2/6394) | 0·00 (0/170 065) | 0·022 (6/277 014) | 0·001 |
| Hepatitis A | 0·00 (0/7706) | 0·00 (0/3187) | 0·33 (6/18 374) | 0·15 (9/61 998) | 0·269 |

$ *p*-values were based on Fisher’s exact test.

**Table S11. Comparison of node strength and node betweenness between the provincial capital cities and other Chinese cities.**

| **Disease/City type** | **Node strength** | | | | | **Node betweenness** | | | | |
| --- | --- | --- | --- | --- | --- | --- | --- | --- | --- | --- |
|  | **Mean** | **Q25** | **Q50** | **Q75** | ***P* value^a^** | **Mean** | **Q25** | **Q50** | **Q75** | ***P* value^a^** |
| **Hepatitis B** | | | | | | | | | | |
| Other cities | 3167·4 | 1167 | 2176 | 4382 | <0·001 | 0·003 | 0·001 | 0·002 | 0·004 | <0·001 |
| Provincial capitals | 18 229·2 | 5523 | 13 286 | 22 924 |  | 0·005 | 0·004 | 0·005 | 0·006 |  |
| **Tuberculosis** | | | | | | | | | | |
| Other cities | 1886·7 | 806 | 1403 | 2506 | <0·001 | 0·003 | 0·001 | 0·002 | 0·004 | <0·001 |
| Provincial capitals | 11 434·4 | 5188 | 10 041 | 15 388 |  | 0·008 | 0·005 | 0·008 | 0·009 |  |
| **Hand-foot-and-mouth disease** | | | | | | | | | | |
| Other cities | 1524·2 | 357 | 817 | 1990 | <0·001 | 0·003 | 0·001 | 0·002 | 0·003 | <0·001 |
| Provincial capitals | 7056·4 | 2064 | 5777 | 9161 |  | 0·013 | 0·008 | 0·011 | 0·018 |  |
| **Syphilis** | | | | | | | | | | |
| Other cities | 1292·3 | 724 | 1053 | 1786 | <0·001 | 0·003 | 0·001 | 0·002 | 0·003 | <0·001 |
| Provincial capitals | 7034·1 | 3628 | 5494 | 9258 |  | 0·007 | 0·005 | 0·007 | 0·009 |  |
| **Influenza** | | | | | | | | | | |
| Other cities | 833·9 | 208 | 448 | 989 | <0·001 | 0·003 | 0·001 | 0·002 | 0·004 | <0·001 |
| Provincial capitals | 4777·0 | 896 | 1835 | 5301 |  | 0·014 | 0·006 | 0·013 | 0·018 |  |
| **Hepatitis C** | | | | | | | | | | |
| Other cities | 580·2 | 222 | 400 | 719 | <0·001 | 0·003 | 0·001 | 0·002 | 0·004 | <0·001 |
| Provincial capitals | 3421·9 | 1502 | 2591 | 3748 |  | 0·014 | 0·008 | 0·011 | 0·016 |  |
| **HIV/AIDS** | | | | | | | | | | |
| Other cities | 529·7 | 177 | 358 | 635 | <0·001 | 0·002 | 0·001 | 0·002 | 0·003 | <0·001 |
| Provincial capitals | 2699·9 | 1121 | 1716 | 4241 |  | 0·011 | 0·007 | 0·010 | 0·013 |  |
| **Gonorrhea** | | | | | | | | | | |
| Other cities | 351·2 | 122 | 238 | 456 | <0·001 | 0·003 | 0·001 | 0·002 | 0·004 | <0·001 |
| Provincial capitals | 1454·3 | 349 | 1151 | 1895 |  | 0·013 | 0·006 | 0·010 | 0·014 |  |
| **Brucellosis** | | | | | | | | | | |
| Other cities | 174·2 | 12 | 32 | 182 | <0·001 | 0·004 | 0·000 | 0·001 | 0·006 | <0·001 |
| Provincial capitals | 848·5 | 90 | 233 | 1042 |  | 0·026 | 0·006 | 0·015 | 0·036 |  |
| **Mumps** | | | | | | | | | | |
| Other cities | 116·0 | 39 | 79 | 142 | <0·001 | 0·004 | 0·001 | 0·002 | 0·004 | <0·001 |
| Provincial capitals | 519·8 | 230 | 455 | 608 |  | 0·024 | 0·011 | 0·018 | 0·027 |  |
| **Hepatitis E** | | | | | | | | | | |
| Other cities | 85·0 | 28 | 60 | 113 | <0·001 | 0·004 | 0·000 | 0·002 | 0·005 | <0·001 |
| Provincial capitals | 576·8 | 214 | 394 | 699 |  | 0·034 | 0·012 | 0·023 | 0·051 |  |
| **Pertussis** | | | | | | | | | | |
| Other cities | 71·0 | 7 | 21 | 64 | <0·001 | 0·003 | 0·000 | 0·000 | 0·001 | <0·001 |
| Provincial capitals | 523·5 | 48 | 115 | 341 |  | 0·019 | 0·004 | 0·007 | 0·019 |  |
| **Shigellosis** | | | | | | | | | | |
| Other cities | 50·5 | 20 | 34 | 61 | <0·001 | 0·004 | 0·001 | 0·002 | 0·006 | <0·001 |
| Provincial capitals | 218·1 | 70 | 157 | 288 |  | 0·030 | 0·011 | 0·018 | 0·031 |  |
| **Hepatitis A** | | | | | | | | | | |
| Other cities | 42·7 | 17 | 30 | 49 | <0·001 | 0·005 | 0·000 | 0·003 | 0·007 | <0·001 |
| Provincial capitals | 269·4 | 111 | 148 | 329 |  | 0·033 | 0·012 | 0·026 | 0·045 |  |

^a^*P* values were calculated from the Wilcox rank sum test.

**Table S12. Top 20 cities ranked by node strength and betweenness in the migration network of infectious diseases. Underlined cities are provincial capitals.**

| **Rank** | **City** | **Node strength** | **City** | **Node betweenness** |
| --- | --- | --- | --- | --- |
| **Hepatitis B** | | | | |
| 1 | Guangzhou | 105 410 | Mianyang | 0·014 |
| 2 | Zhengzhou | 59 811 | Chengdu | 0·013 |
| 3 | Changsha | 34 107 | Chongqing | 0·013 |
| 4 | Jinan | 31 244 | Heze | 0·010 |
| 5 | Nanning | 25 941 | Wuhan | 0·010 |
| 6 | Shanghai | 24 257 | Putian | 0·010 |
| 7 | Hefei | 23 684 | Hefei | 0·009 |
| 8 | Haikou | 23 498 | Fuzhou | 0·009 |
| 9 | Wenchang | 22 633 | Nanchong | 0·008 |
| 10 | Taiyuan | 22 349 | Quanzhou | 0·008 |
| 11 | Wuhan | 22 275 | Zhoukou | 0·008 |
| 12 | Xining | 22 253 | Luzhou | 0·008 |
| 13 | Fuzhou | 21 952 | Nanchang | 0·008 |
| 14 | Foshan | 21 741 | Shenzhen | 0·007 |
| 15 | Urumqi | 21 237 | Yuncheng | 0·007 |
| 16 | Zhoukou | 17 323 | Shangqiu | 0·007 |
| 17 | Chengdu | 15 113 | Dazhou | 0·007 |
| 18 | Yongzhou | 14 636 | Baoding | 0·007 |
| 19 | Nanchang | 14 609 | Shiyan | 0·007 |
| 20 | Chongqing | 13 286 | Lanzhou | 0·007 |
| **Tuberculosis** | | | | |
| 1 | Guangzhou | 27 895 | Chongqing | 0·021 |
| 2 | Changsha | 26 912 | Chengdu | 0·017 |
| 3 | Xian | 24 788 | Bijie | 0·014 |
| 4 | Chengdu | 20 541 | Wuhan | 0·013 |
| 5 | Xining | 19 847 | Zunyi | 0·013 |
| 6 | Zhengzhou | 19 587 | Tianjin | 0·013 |
| 7 | Beijing | 18 419 | Baoding | 0·012 |
| 8 | Harbin | 16 687 | Dalian | 0·012 |
| 9 | Wuhan | 14 090 | Guiyang | 0·012 |
| 10 | Hefei | 12 624 | Shenzhen | 0·012 |
| 11 | Kunming | 12 461 | Harbin | 0·012 |
| 12 | Jinan | 11 175 | Wenzhou | 0·011 |
| 13 | Nanning | 11 153 | Ili | 0·011 |
| 14 | Nanchang | 11 127 | Cangzhou | 0·011 |
| 15 | Chongqing | 10 790 | Qianxinan | 0·010 |
| 16 | Liuzhou | 10 331 | Luzhou | 0·010 |
| 17 | Shanghai | 10 041 | Zhengzhou | 0·010 |
| 18 | Hangzhou | 9541 | Xining | 0·009 |
| 19 | Shijiazhuang | 9267 | Changchun | 0·009 |
| 20 | Bijie | 8956 | Xian | 0·009 |
| **Hand-foot-and-mouth disease** | | | | |
| 1 | Guangzhou | 32 166 | Chongqing | 0·035 |
| 2 | Nanjing | 19 766 | Xian | 0·035 |
| 3 | Changsha | 17 031 | Beijing | 0·032 |
| 4 | Foshan | 14 860 | Chengdu | 0·027 |
| 5 | Shanghai | 13 381 | Shenzhen | 0·026 |
| 6 | Suzhou | 12 277 | Wenzhou | 0·021 |
| 7 | Xian | 11 513 | Fuyang | 0·021 |
| 8 | Beijing | 11 250 | Nanjing | 0·019 |
| 9 | Nanning | 11 146 | Shanghai | 0·018 |
| 10 | Langfang | 10 256 | Fuzhou | 0·018 |
| 11 | Chengdu | 9575 | Changsha | 0·018 |
| 12 | Shenzhen | 9120 | Tianjin | 0·018 |
| 13 | Wenchang | 8932 | Hangzhou | 0·017 |
| 14 | Chongqing | 8747 | Guangzhou | 0·016 |
| 15 | Zhengzhou | 8525 | Hefei | 0·013 |
| 16 | Dongguan | 8327 | Baoding | 0·013 |
| 17 | Zhaoqing | 8027 | Guiyang | 0·013 |
| 18 | Huizhou | 7815 | Suzhou | 0·013 |
| 19 | Haikou | 7801 | Lanzhou | 0·013 |
| 20 | Hefei | 7361 | Heze | 0·012 |
| **Syphilis** | | | | |
| 1 | Guangzhou | 24 058 | Chengdu | 0·014 |
| 2 | Shanghai | 14 094 | Fuzhou | 0·012 |
| 3 | Hangzhou | 12 242 | Nanchong | 0·012 |
| 4 | Chengdu | 12 170 | Xining | 0·011 |
| 5 | Changsha | 12 030 | Chongqing | 0·011 |
| 6 | Zhengzhou | 9993 | Mianyang | 0·011 |
| 7 | Xian | 9933 | Tianjin | 0·011 |
| 8 | Chongqing | 9323 | Wuhan | 0·010 |
| 9 | Hefei | 9194 | Fuyang | 0·009 |
| 10 | Shenyang | 8982 | Nanchang | 0·009 |
| 11 | Beijing | 8964 | Shenyang | 0·009 |
| 12 | Fuzhou | 7719 | Zhengzhou | 0·009 |
| 13 | Kunming | 7259 | Wenzhou | 0·009 |
| 14 | Xining | 7014 | Hefei | 0·008 |
| 15 | Urumqi | 5644 | Deyang | 0·008 |
| 16 | Wuhan | 5494 | Xian | 0·008 |
| 17 | Wenchang | 5329 | Jinan | 0·008 |
| 18 | Guiyang | 5177 | Changsha | 0·008 |
| 19 | Taiyuan | 5038 | Qingdao | 0·007 |
| 20 | Wenzhou | 5026 | Beijing | 0·007 |
| **Influenza** | | | | |
| 1 | Beijing | 24 757 | Tianjin | 0·033 |
| 2 | Guangzhou | 21 250 | Shanghai | 0·03 |
| 3 | Changsha | 19 125 | Wuhan | 0·03 |
| 4 | Hangzhou | 15 834 | Chengdu | 0·029 |
| 5 | Langfang | 12 431 | Xian | 0·027 |
| 6 | Zhengzhou | 9158 | Chongqing | 0·025 |
| 7 | Xian | 9073 | Shenzhen | 0·023 |
| 8 | Foshan | 7995 | Baoding | 0·020 |
| 9 | Chengdu | 7295 | Beijing | 0·020 |
| 10 | Wuhan | 6581 | Shenyang | 0·018 |
| 11 | Shenzhen | 6005 | Changsha | 0·018 |
| 12 | Zhongshan | 5451 | Zhengzhou | 0·017 |
| 13 | Zhuhai | 5208 | Jinhua | 0·017 |
| 14 | Shaoxing | 4650 | Urumqi | 0·017 |
| 15 | Xianyang | 4367 | Hangzhou | 0·017 |
| 16 | Jinhua | 4107 | Yichang | 0·016 |
| 17 | Kunming | 4021 | Mianyang | 0·016 |
| 18 | Yueyang | 3892 | Luoyang | 0·015 |
| 19 | Jiaxing | 3741 | Nanjing | 0·015 |
| 20 | Haikou | 3711 | Lanzhou | 0·014 |
| **Hepatitis C** | | | | |
| 1 | Zhengzhou | 20 561 | Chongqing | 0·051 |
| 2 | Guangzhou | 12 484 | Xian | 0·031 |
| 3 | Changsha | 7521 | Chengdu | 0·025 |
| 4 | Taiyuan | 4877 | Zhengzhou | 0·025 |
| 5 | Zhumadian | 4835 | Beijing | 0·023 |
| 6 | Xian | 4617 | Wuhan | 0·020 |
| 7 | Zhoukou | 4514 | Changsha | 0·019 |
| 8 | Kunming | 4183 | Wenzhou | 0·018 |
| 9 | Nanning | 4010 | Shenyang | 0·017 |
| 10 | Urumqi | 3942 | Guangzhou | 0·015 |
| 11 | Xining | 3554 | Urumqi | 0·015 |
| 12 | Shanghai | 3209 | Huaihua | 0·014 |
| 13 | Hefei | 3005 | Mianyang | 0·014 |
| 14 | Wuhan | 2958 | Zhumadian | 0·014 |
| 15 | Wenchang | 2930 | Shenzhen | 0·014 |
| 16 | Chengdu | 2788 | Liangshan | 0·013 |
| 17 | Lanzhou | 2784 | Kunming | 0·013 |
| 18 | Jinan | 2592 | Xining | 0·012 |
| 19 | Haikou | 2591 | Harbin | 0·012 |
| 20 | Foshan | 2446 | Fuzhou | 0·011 |
| **HIV/AIDS** | | | | |
| 1 | Chengdu | 11 130 | Chongqing | 0·029 |
| 2 | Guangzhou | 6368 | Chengdu | 0·028 |
| 3 | Liangshan | 5474 | Tianjin | 0·026 |
| 4 | Nanning | 5248 | Shenzhen | 0·019 |
| 5 | Chongqing | 5084 | Xian | 0·019 |
| 6 | Beijing | 4802 | Hangzhou | 0·018 |
| 7 | Shanghai | 4370 | Beijing | 0·016 |
| 8 | Kunming | 4291 | Shanghai | 0·015 |
| 9 | Changsha | 4243 | Liangshan | 0·015 |
| 10 | Zhengzhou | 4239 | Suzhou | 0·014 |
| 11 | Guiyang | 3630 | Wuhan | 0·014 |
| 12 | Wuhan | 3205 | Changsha | 0·013 |
| 13 | Shenzhen | 2948 | Guangzhou | 0·012 |
| 14 | Xian | 2939 | Nanjing | 0·012 |
| 15 | Liuzhou | 2936 | Ningbo | 0·011 |
| 16 | Hangzhou | 2909 | Jinhua | 0·011 |
| 17 | Foshan | 2548 | Zhengzhou | 0·011 |
| 18 | Bijie | 2545 | Shijiazhuang | 0·011 |
| 19 | Dongguan | 2358 | Jinan | 0·011 |
| 20 | Laibin | 2286 | Kunming | 0·011 |
| **Gonorrhea** | | | | |
| 1 | Hangzhou | 4859 | Chongqing | 0·056 |
| 2 | Guangzhou | 4792 | Chengdu | 0·038 |
| 3 | Nanning | 3634 | Zhengzhou | 0·028 |
| 4 | Changsha | 3194 | Changsha | 0·023 |
| 5 | Foshan | 3154 | Beijing | 0·021 |
| 6 | Shanghai | 3060 | Xian | 0·020 |
| 7 | Jinhua | 2606 | Wenzhou | 0·020 |
| 8 | Zhengzhou | 2474 | Hefei | 0·017 |
| 9 | Jiaxing | 2251 | Nantong | 0·017 |
| 10 | Chongqing | 2103 | Hangzhou | 0·015 |
| 11 | Shaoxing | 2040 | Suzhou | 0·013 |
| 12 | Kunming | 1938 | Shenzhen | 0·013 |
| 13 | Wenzhou | 1868 | Shanghai | 0·013 |
| 14 | Xian | 1852 | Quanzhou | 0·013 |
| 15 | Wenchang | 1780 | Nanjing | 0·012 |
| 16 | Xiamen | 1732 | Fuzhou | 0·012 |
| 17 | Suzhou | 1714 | Yancheng | 0·011 |
| 18 | Chengdu | 1670 | Changchun | 0·011 |
| 19 | Haikou | 1543 | Urumqi | 0·011 |
| 20 | Nanjing | 1470 | Taizhou | 0·011 |
| **Brucellosis** | | | | |
| 1 | Urumqi | 4612 | Harbin | 0·103 |
| 2 | Harbin | 4230 | Guangzhou | 0·082 |
| 3 | Suihua | 2857 | Beijing | 0·081 |
| 4 | Holhot | 2839 | Urumqi | 0·080 |
| 5 | Hulunbuir | 2818 | Holhot | 0·071 |
| 6 | Qiqihar | 2458 | Ili | 0·06 |
| 7 | Jinan | 2334 | Lanzhou | 0·045 |
| 8 | Changji | 1971 | Shanghai | 0·041 |
| 9 | Yinchuan | 1695 | Zhengzhou | 0·04 |
| 10 | Shenyang | 1661 | Chongqing | 0·033 |
| 11 | Zhengzhou | 1394 | Aksu | 0·030 |
| 12 | Ulanqab | 1186 | Ulanqab | 0·029 |
| 13 | Tongliao | 1146 | Shenzhen | 0·028 |
| 14 | Jinzhou | 1076 | Mudanjiang | 0·027 |
| 15 | Lanzhou | 1065 | Hangzhou | 0·026 |
| 16 | Wuzhong | 1048 | Qiqihar | 0·025 |
| 17 | Taiyuan | 1019 | Dongguan | 0·024 |
| 18 | Heihe | 1010 | Anyang | 0·024 |
| 19 | Ili | 972 | Xvzhou | 0·023 |
| 20 | Xilingol | 962 | Hulunbuir | 0·023 |
| **Mumps** | | | | |
| 1 | Changsha | 2103 | Beijing | 0·094 |
| 2 | Taiyuan | 1130 | Chongqing | 0·077 |
| 3 | Zhengzhou | 1063 | Changsha | 0·055 |
| 4 | Wenchang | 1049 | Chengdu | 0·051 |
| 5 | Beijing | 957 | Zhengzhou | 0·043 |
| 6 | Haikou | 924 | Shenzhen | 0·042 |
| 7 | Guangzhou | 822 | Guangzhou | 0·037 |
| 8 | Langfang | 815 | Xian | 0·031 |
| 9 | Xian | 740 | Lanzhou | 0·030 |
| 10 | Wuhan | 657 | Suzhou | 0·028 |
| 11 | Tianjin | 559 | Harbin | 0·024 |
| 12 | Hefei | 555 | Tianjin | 0·024 |
| 13 | Chengdu | 544 | Wuhan | 0·024 |
| 14 | Foshan | 539 | Wenzhou | 0·022 |
| 15 | Nanning | 535 | Shijiazhuang | 0·021 |
| 16 | Shijiazhuang | 530 | Shangqiu | 0·021 |
| 17 | Bijie | 487 | Shanghai | 0·020 |
| 18 | Chongqing | 479 | Hangzhou | 0·019 |
| 19 | Nanchang | 463 | Kunming | 0·019 |
| 20 | Shenzhen | 458 | Hefei | 0·018 |
| **Hepatitis E** | | | | |
| 1 | Guangzhou | 2707 | Chongqing | 0·101 |
| 2 | Kunming | 1912 | Guangzhou | 0·091 |
| 3 | Wuhan | 1597 | Chengdu | 0·083 |
| 4 | Haikou | 1408 | Beijing | 0·076 |
| 5 | Wenchang | 1285 | Wuhan | 0·067 |
| 6 | Hangzhou | 1027 | Hefei | 0·064 |
| 7 | Taiyuan | 876 | Hangzhou | 0·062 |
| 8 | Hefei | 775 | Shanghai | 0·054 |
| 9 | Changsha | 713 | Shenzhen | 0·052 |
| 10 | Shanghai | 685 | Wenzhou | 0·051 |
| 11 | Shenyang | 640 | Harbin | 0·048 |
| 12 | Beijing | 553 | Xian | 0·046 |
| 13 | Chengdu | 510 | Kunming | 0·045 |
| 14 | Zhengzhou | 475 | Shenyang | 0·036 |
| 15 | Suzhou | 458 | Urumqi | 0·033 |
| 16 | Chongqing | 450 | Changsha | 0·031 |
| 17 | Qujing | 432 | Nanjing | 0·030 |
| 18 | Nanjing | 407 | Xinyang | 0·025 |
| 19 | Guiyang | 394 | Shijiazhuang | 0·023 |
| 20 | Foshan | 382 | Wuxi | 0·023 |
| **Pertussis** | | | | |
| 1 | Jinan | 4869 | Shenzhen | 0·137 |
| 2 | Xian | 3020 | Xian | 0·123 |
| 3 | Chongqing | 2440 | Chongqing | 0·106 |
| 4 | Tianjin | 1027 | Changsha | 0·062 |
| 5 | Dezhou | 904 | Jinan | 0·048 |
| 6 | Changsha | 891 | Shanghai | 0·039 |
| 7 | Jining | 875 | Wenzhou | 0·035 |
| 8 | Langfang | 864 | Jingzhou | 0·034 |
| 9 | Linyi | 835 | Yulin | 0·032 |
| 10 | Xianyang | 733 | Beijing | 0·029 |
| 11 | Beijing | 585 | Liaocheng | 0·028 |
| 12 | Weinan | 547 | Baoding | 0·028 |
| 13 | Shenzhen | 524 | Tianjin | 0·028 |
| 14 | Liaocheng | 507 | Nanchong | 0·024 |
| 15 | Heze | 505 | Lianyungang | 0·024 |
| 16 | Taiyuan | 449 | Dazhou | 0·021 |
| 17 | Dazhou | 437 | Nanchang | 0·021 |
| 18 | Guangan | 435 | Suzhou | 0·020 |
| 19 | Binzhou | 420 | Jining | 0·020 |
| 20 | Zunyi | 390 | Baotou | 0·019 |
| **Shigellosis** | | | | |
| 1 | Beijing | 774 | Beijing | 0·161 |
| 2 | Xian | 621 | Chongqing | 0·154 |
| 3 | Tianjin | 592 | Tianjin | 0·076 |
| 4 | Lanzhou | 438 | Xian | 0·069 |
| 5 | Lassa | 424 | Wuhan | 0·041 |
| 6 | Hangzhou | 422 | Shenzhen | 0·039 |
| 7 | Chongqing | 371 | Qingdao | 0·038 |
| 8 | Liangshan | 317 | Guangzhou | 0·037 |
| 9 | Langfang | 306 | Lanzhou | 0·035 |
| 10 | Chengdu | 290 | Taiyuan | 0·035 |
| 11 | Hefei | 285 | Chengdu | 0·028 |
| 12 | Wuhan | 282 | Dalian | 0·027 |
| 13 | Shenyang | 270 | Liangshan | 0·027 |
| 14 | Panzhihua | 269 | Hefei | 0·023 |
| 15 | Qiqihar | 263 | Suzhou | 0·023 |
| 16 | Dali | 232 | Shijiazhuang | 0·023 |
| 17 | Jiamusi | 217 | Lassa | 0·022 |
| 18 | Naqu | 217 | Xvzhou | 0·022 |
| 19 | Xvzhou | 210 | Shangqiu | 0·022 |
| 20 | Suzhou | 203 | Kunming | 0·021 |
| **Hepatitis A** | | | | |
| 1 | Taiyuan | 1577 | Chongqing | 0·169 |
| 2 | Chengdu | 775 | Guangzhou | 0·083 |
| 3 | Kunming | 650 | Chengdu | 0·072 |
| 4 | Xining | 596 | Beijing | 0·072 |
| 5 | Urumqi | 466 | Shanghai | 0·069 |
| 6 | Shenyang | 419 | Dalian | 0·068 |
| 7 | Guangzhou | 392 | Liangshan | 0·060 |
| 8 | Xinzhou | 388 | Shenyang | 0·054 |
| 9 | Lvliang | 388 | Hangzhou | 0·051 |
| 10 | Lanzhou | 385 | Changsha | 0·045 |
| 11 | Jinzhong | 326 | Taiyuan | 0·044 |
| 12 | Chongqing | 273 | Nanchong | 0·044 |
| 13 | Changsha | 252 | Urumqi | 0·042 |
| 14 | Liangshan | 241 | Luzhou | 0·038 |
| 15 | Wuhan | 235 | Fuzhou | 0·038 |
| 16 | Ganzi | 230 | Harbin | 0·036 |
| 17 | Shanghai | 210 | Wenzhou | 0·033 |
| 18 | Kashgar | 204 | Xvzhou | 0·031 |
| 19 | Beijing | 197 | Kunming | 0·030 |
| 20 | Yushu | 188 | Shenzhen | 0·030 |

**Table S13. Prediction performance metrics of the XGBoost models and the gravity models for infectious disease migration networks. Metric values showed in this table were calculated based on the test dataset (30% of total data).**

| **Disease** | **XGBoost model** | | | **Gravity model** | | |
| --- | --- | --- | --- | --- | --- | --- |
|  | **CPC** | **PCC** | **RMSLE** | **CPC** | **PCC** | **RMSLE** |
| Hepatitis B | 0·712 | 0·876 | 0·425 | 0·429 | 0·540 | 0·645 |
| Tuberculosis | 0·697 | 0·867 | 0·389 | 0·340 | 0·501 | 0·512 |
| HFMD | 0·628 | 0·822 | 0·323 | 0·407 | 0·627 | 0·423 |
| Syphilis | 0·735 | 0·917 | 0·387 | 0·396 | 0·623 | 0·510 |
| Influenza | 0·685 | 0·775 | 0·305 | 0·447 | 0·699 | 0·435 |
| Hepatitis C | 0·669 | 0·877 | 0·292 | 0·448 | 0·499 | 0·381 |
| HIV/AIDS | 0·711 | 0·902 | 0·326 | 0·465 | 0·663 | 0·389 |
| Gonorrhea | 0·706 | 0·937 | 0·280 | 0·344 | 0·465 | 0·401 |
| Brucellosis | 0·567 | 0·722 | 0·171 | 0·288 | 0·404 | 0·297 |
| Mumps | 0·547 | 0·777 | 0·195 | 0·470 | 0·647 | 0·221 |
| Hepatitis E | 0·622 | 0·810 | 0·160 | 0·480 | 0·683 | 0·195 |
| Pertussis | 0·618 | 0·895 | 0·118 | 0·201 | 0·691 | 0·182 |
| Shigellosis | 0·547 | 0·801 | 0·152 | 0·298 | 0·475 | 0·212 |
| Hepatitis A | 0·613 | 0·915 | 0·122 | 0·347 | 0·297 | 0·155 |

Abbreviations: CPC=common part of commuters; PCC=Pearson correlation coefficient; RMSLE=root mean squared logarithmic error.

**Table S14. Region distribution of migrant workers in China during 2015 to 2020 (×10 000 person).^$^**

| **Region#** |  | **Inter-province Flow** | **Intra-province Flow** |  | **Importation** | **Exportation** | **Year** |
| --- | --- | --- | --- | --- | --- | --- | --- |
| East |  | 858 | 4086 |  | 10 300 | 16 008 | 2015 |
|  |  | 837 | 3854 |  | 10 400 | 15 960 | 2016 |
|  |  | 826 | 3888 |  | 10 430 | 15 993 | 2017 |
|  |  | 812 | 3906 |  | 10 410 | 15 808 | 2018 |
|  |  | 821 | 3917 |  | 10 416 | 15 700 | 2019 |
|  |  | 719 | 3905 |  | 10 124 | 15 132 | 2020 |
| Middle |  | 4024 | 2568 |  | 9174 | 5599 | 2015 |
|  |  | 3897 | 2393 |  | 9279 | 5746 | 2016 |
|  |  | 3918 | 2474 |  | 9450 | 5912 | 2017 |
|  |  | 3889 | 2529 |  | 9538 | 6051 | 2018 |
|  |  | 3802 | 2625 |  | 9619 | 6223 | 2019 |
|  |  | 3593 | 2617 |  | 9447 | 6227 | 2020 |
| West |  | 2863 | 2485 |  | 7378 | 5209 | 2015 |
|  |  | 2794 | 2556 |  | 7563 | 5484 | 2016 |
|  |  | 2787 | 2683 |  | 7814 | 5754 | 2017 |
|  |  | 2727 | 2775 |  | 7918 | 5993 | 2018 |
|  |  | 2691 | 2864 |  | 8051 | 6173 | 2019 |
|  |  | 2557 | 2933 |  | 8034 | 6279 | 2020 |
| Northeast |  | * | * |  | 895 | 859 | 2015 |
|  |  | 138 | 465 |  | 929 | 904 | 2016 |
|  |  | 144 | 465 |  | 958 | 914 | 2017 |
|  |  | 166 | 462 |  | 970 | 905 | 2018 |
|  |  | 194 | 457 |  | 991 | 895 | 2019 |
|  |  | 183 | 452 |  | 955 | 853 | 2020 |

$ Data were obtained from the National Bureau of Statistics of China (https://www.stats.gov.cn/), using “survey report on labor workers” (农民工监测调查报告) as a search keyword. Specific websites (last accessed on Oct. 18, 2024) are

https://www.stats.gov.cn/sj/zxfb/202302/t20230203_1899104.html

https://www.stats.gov.cn/sj/zxfb/202302/t20230203_1899495.html

https://www.stats.gov.cn/sj/zxfb/202302/t20230203_1899920.html

https://www.gov.cn/xinwen/2019-04/30/content_5387773.htm

https://www.stats.gov.cn/sj/zxfb/202302/t20230203_1900710.html

# East (Beijing, Tianjin, Hebei, Shanghai, Jiangsu, Zhejiang, Fujian, Shandong, Guangdong, Hainan); Middle (Shanx, Anhui, Jiangxi, Henan, Hubei, Hunan); West (Inner Mongolia, Guangxi, Chongqing, Sichuan, Guizhou, Yunnan, Tibiet, Shaanxi, Gansu, Qinghai, Ningxia, Xinjiang); Northeast (Liaoning, Jilin, Heilongjiang).

* In 2015, Inter- and Intra-province flow of migrant worker of Northeast China were divided into those of East or Middle region of China (Liaoning Province into the East; Heilongjiang and Jilin Province into the Middle).

**Supplemental Figures**

**Figure S1. Temporal and spatial patterns of human movement intensity in China. (A) temporal trend of total inter-city human movement from Jan 1, 2019 to Mar 31, 2020. Peaks are marked by corresponding social events. Chunyun stands for the transportation peak associated with the Chinese New Year. (B) spatial pattern of total inter-city human movement in 2019.**


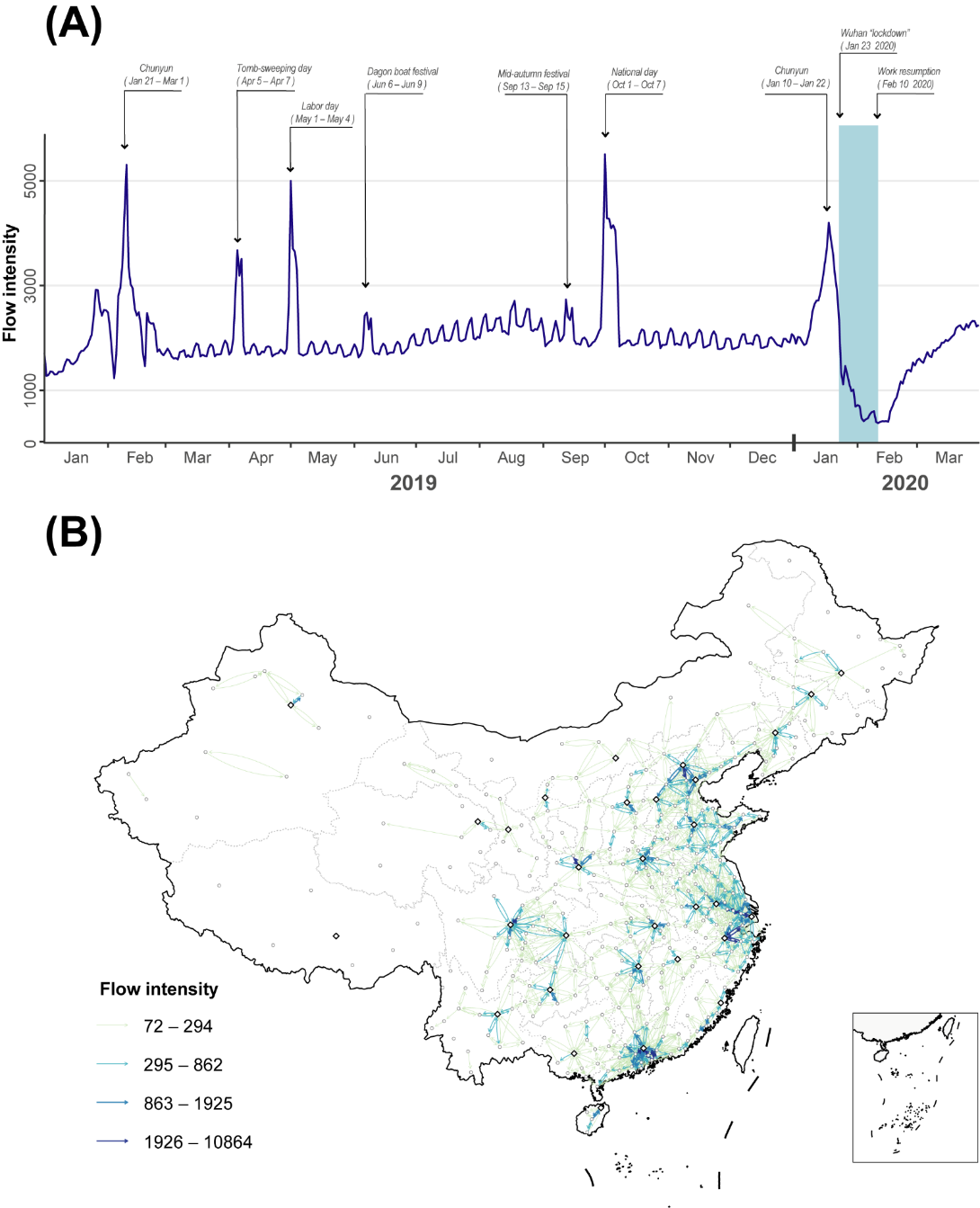


**Figure S2. Spatial pattern of inter-city labor flows in China during 2015.**


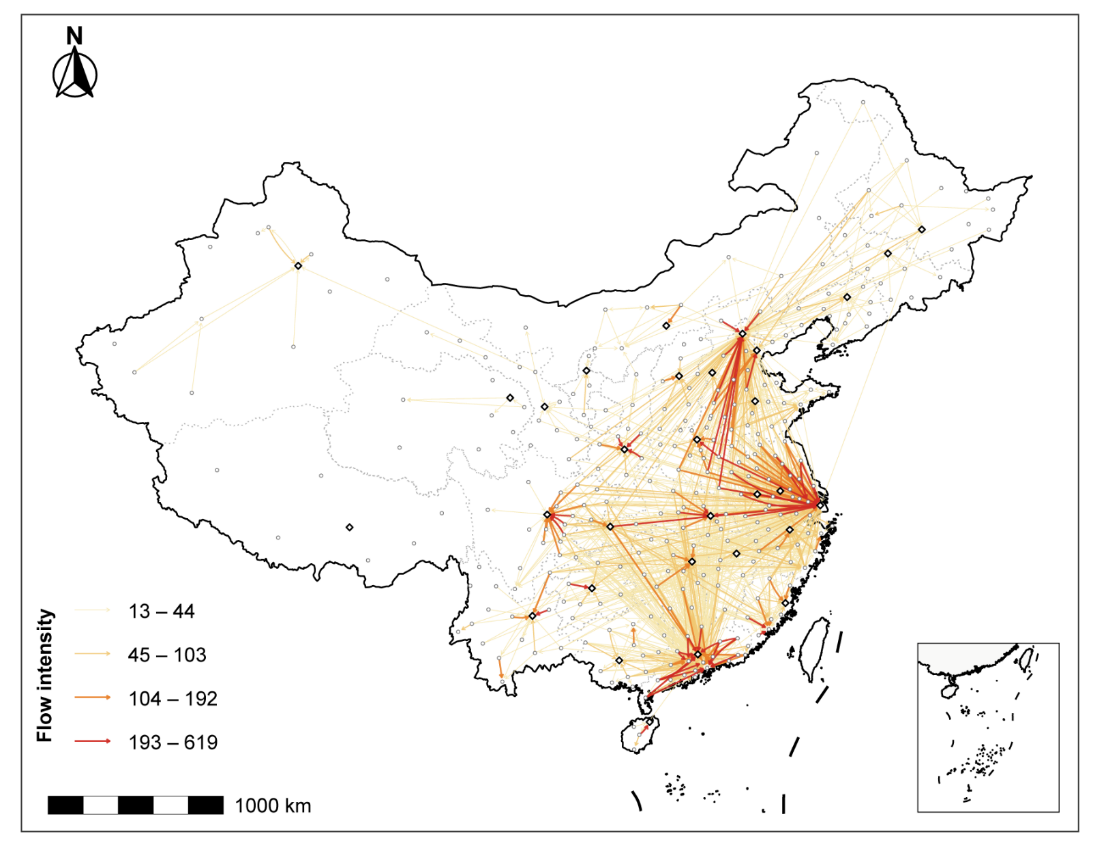


**Figure S3. Example diagram of network statistics.** The nodes ABCDEF are interconnected, and the purple lines in the diagram represent these connections. The thickness of the purple lines indicates the strength of these interconnections (for simplicity, the network in this diagram is undirected). The figure illustrates the strengths of the connections as follows: (A-B 2, A-C 3, B-D 2, B-E 4, C-D 2, C-E 6, D-E 3, E-F 1). The degree of a network node refers to its connectivity with other nodes in the network. For example, in the diagram, node E has a degree of 4 while node C has a degree of 3. The strength of a node is determined not only by the number of connections it has, but also by the strength of those connections. For instance, in this diagram, node E has a strength of 7, while node C has a strength of 11. Network density refers to the ratio between the actual number of connections between nodes in a network and the total possible number of connections between all nodes. In this particular network, the network density is calculated as 8/15 = 0.53. The calculation of betweenness centrality for nodes in the network is based on the shortest paths; specifically, the betweenness centrality of a node refers to the proportion of all shortest paths between any two nodes that pass through that particular node. The betweenness centralities of the nodes in this particular network are as follows: (A 0, B 0.3, C 0, D 0.3, E 0.4, F 0).

**
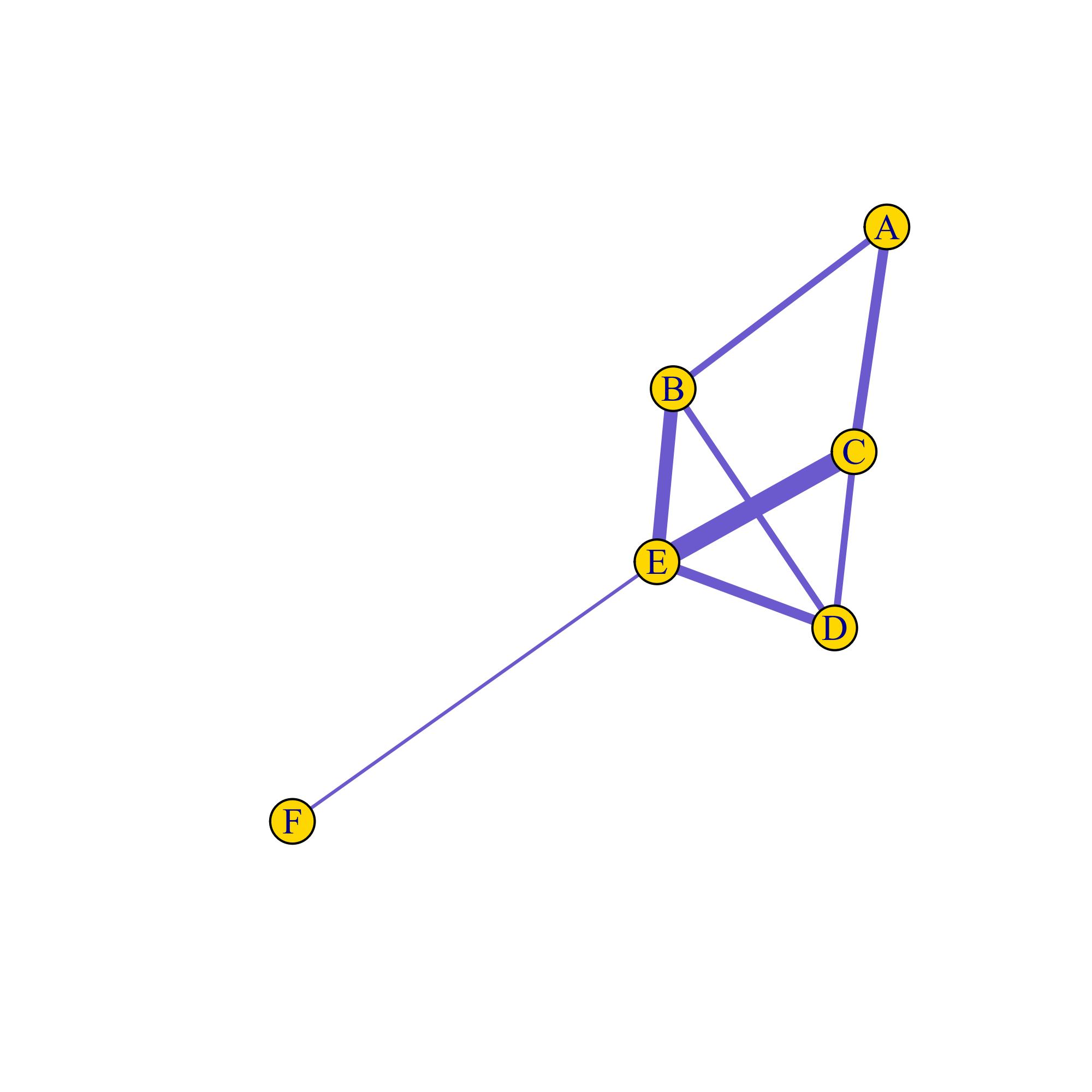
**

**Figure S4. Sensitivity analysis for the network community structure of the inter-city migration network of 14 notifiable infectious diseases when the threshold (the least number of migratory cases for an edge to be included) is changed to 20. Cities in the same color are clustered in the same community (sub-network) by the Louvain algorithm.**


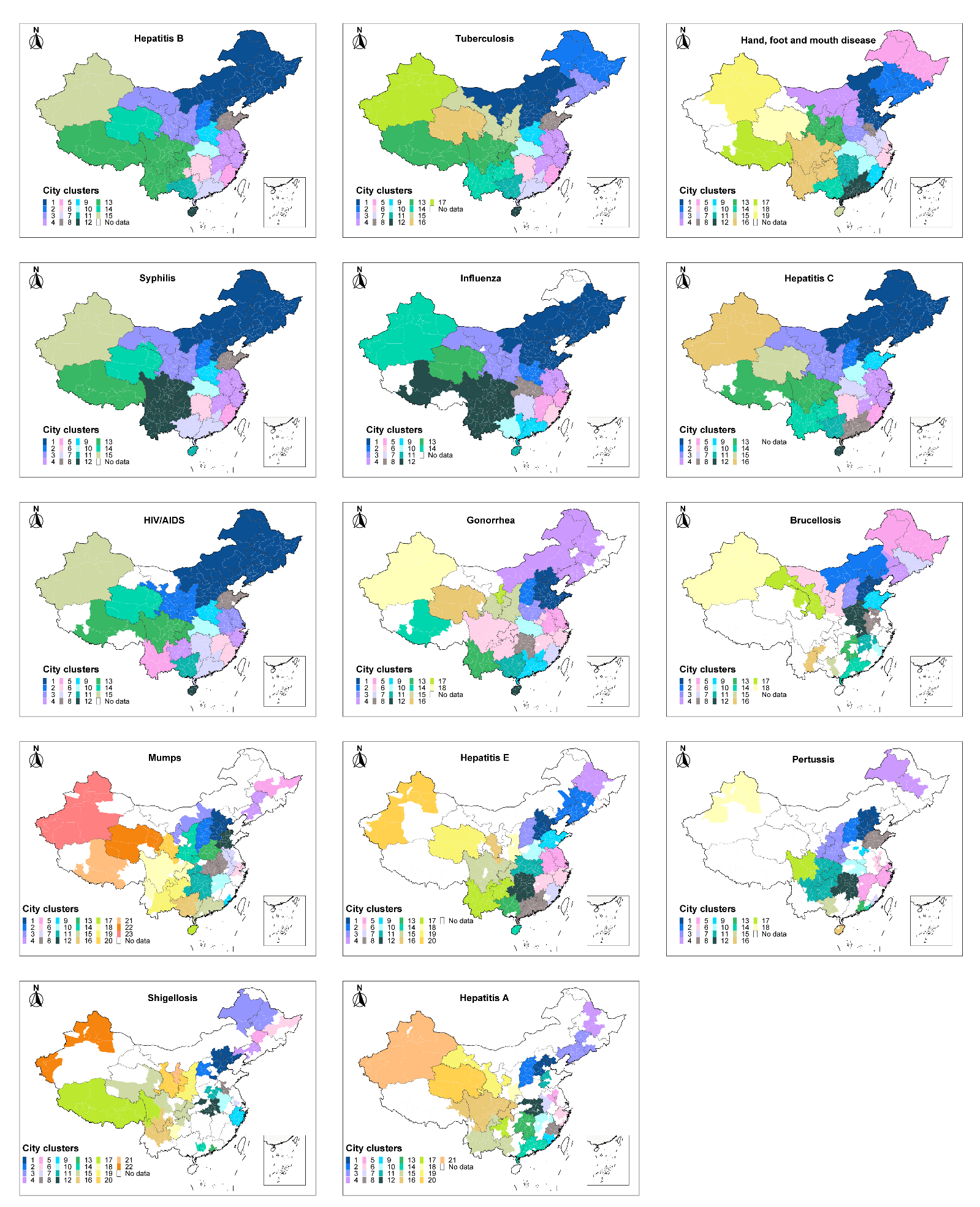


**Figure S5. Age distributions of migratory vs. local cases for the 14 major notifiable infectious diseases in China. Red and blue margins represent excessive portion of migratory cases and local cases, respectively, and grey represents the overlap.**


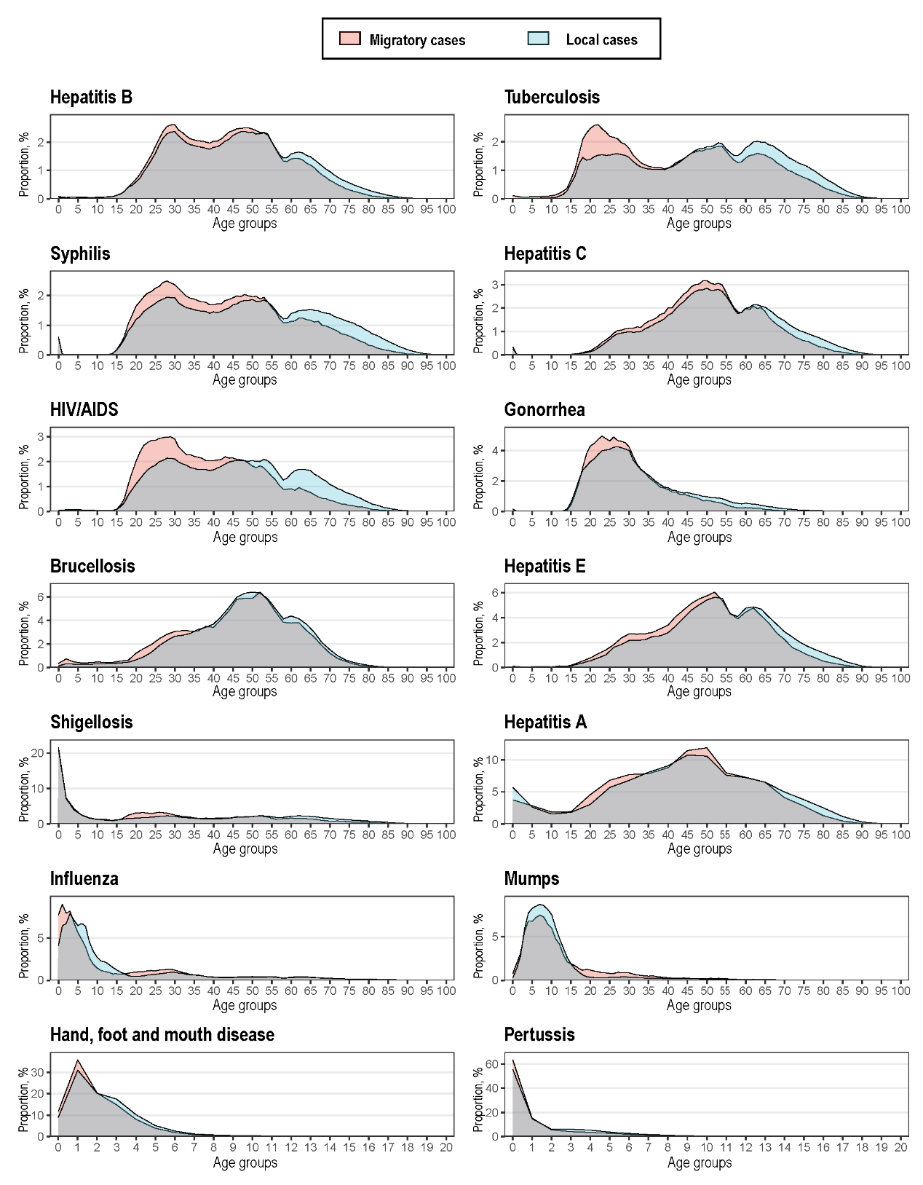


**Figure S6. Temporal trends in the weekly proportion of migratory cases among all reported cases for the 14 major types of notifiable infectious diseases in China between 2016 and 2020. The migratory proportions for each infectious disease were standardized from 0 to 1 according to percentile rank, and represented by the color scale (from 0 to 1; where 1 is the highest proportion and 0 is the lowest proportion).**


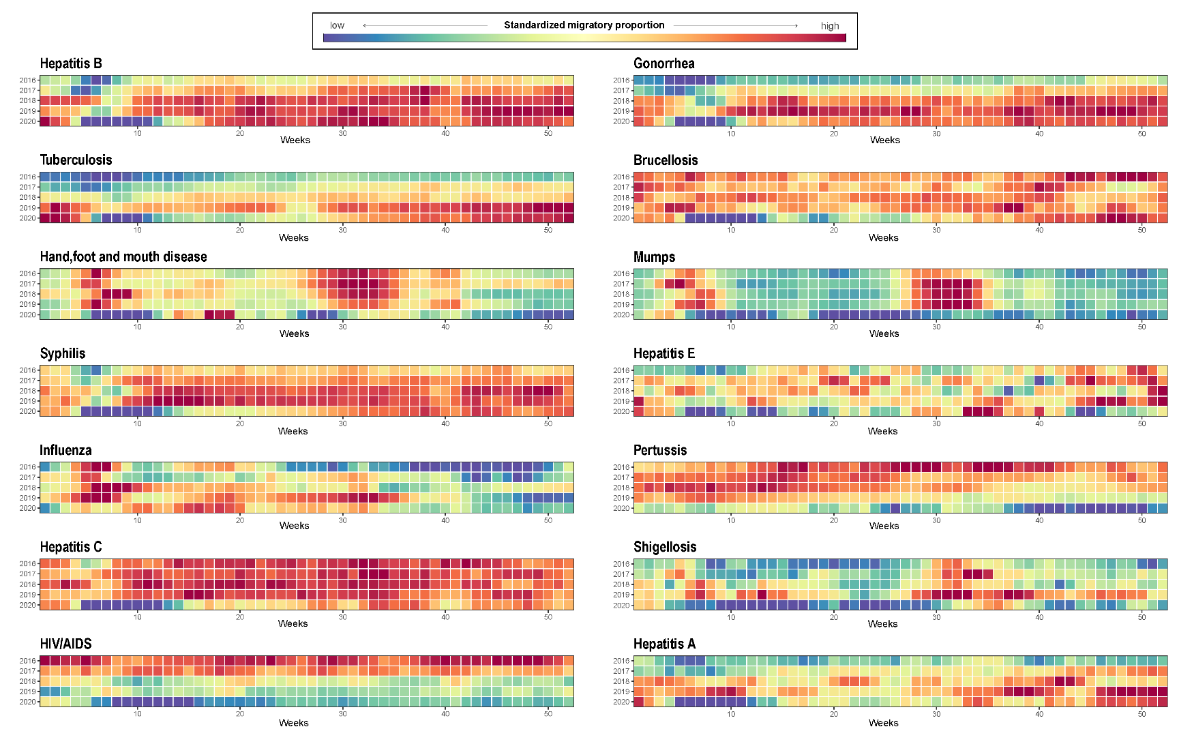


**Figure S7. Temporal trends in the annual proportion of migratory cases among all reported cases for the 14 major types of notifiable infectious diseases in China between 2016 and 2020.**


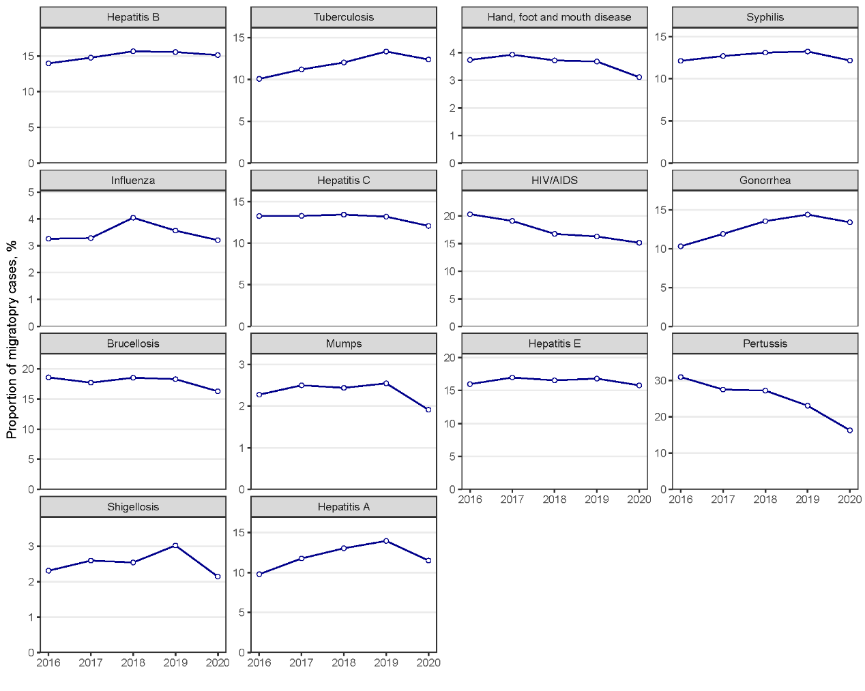


**Figure S8. Spatial distributions of proportions of inflow/outflow cases for 14 major notifiable infectious diseases. These proportions were not calculated for cities with less than 10 reported cases (colored grey) during 2016–2020.**

**
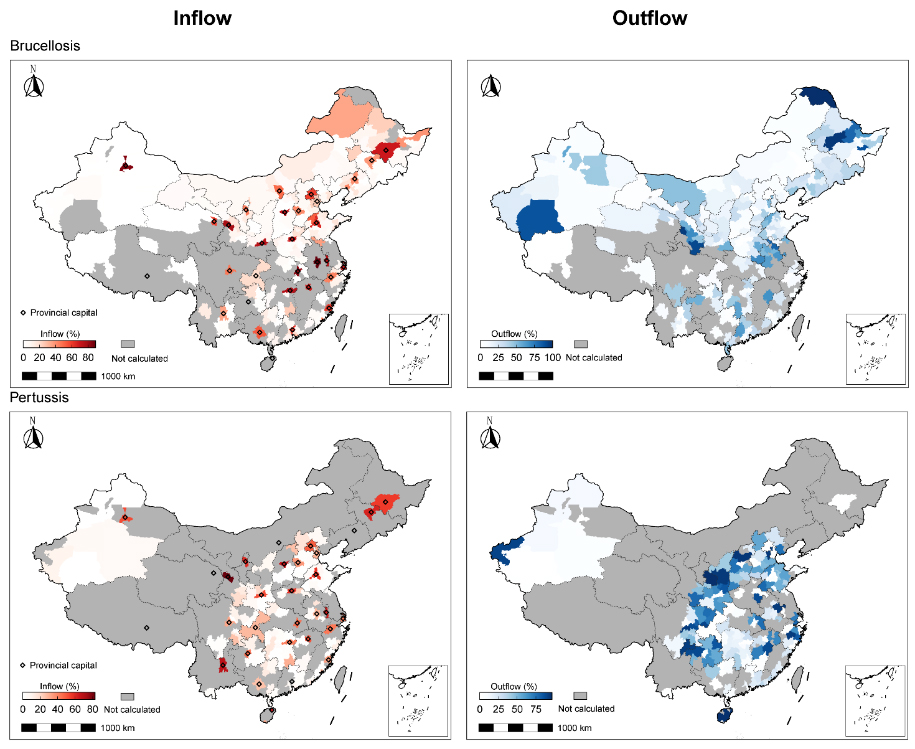
**
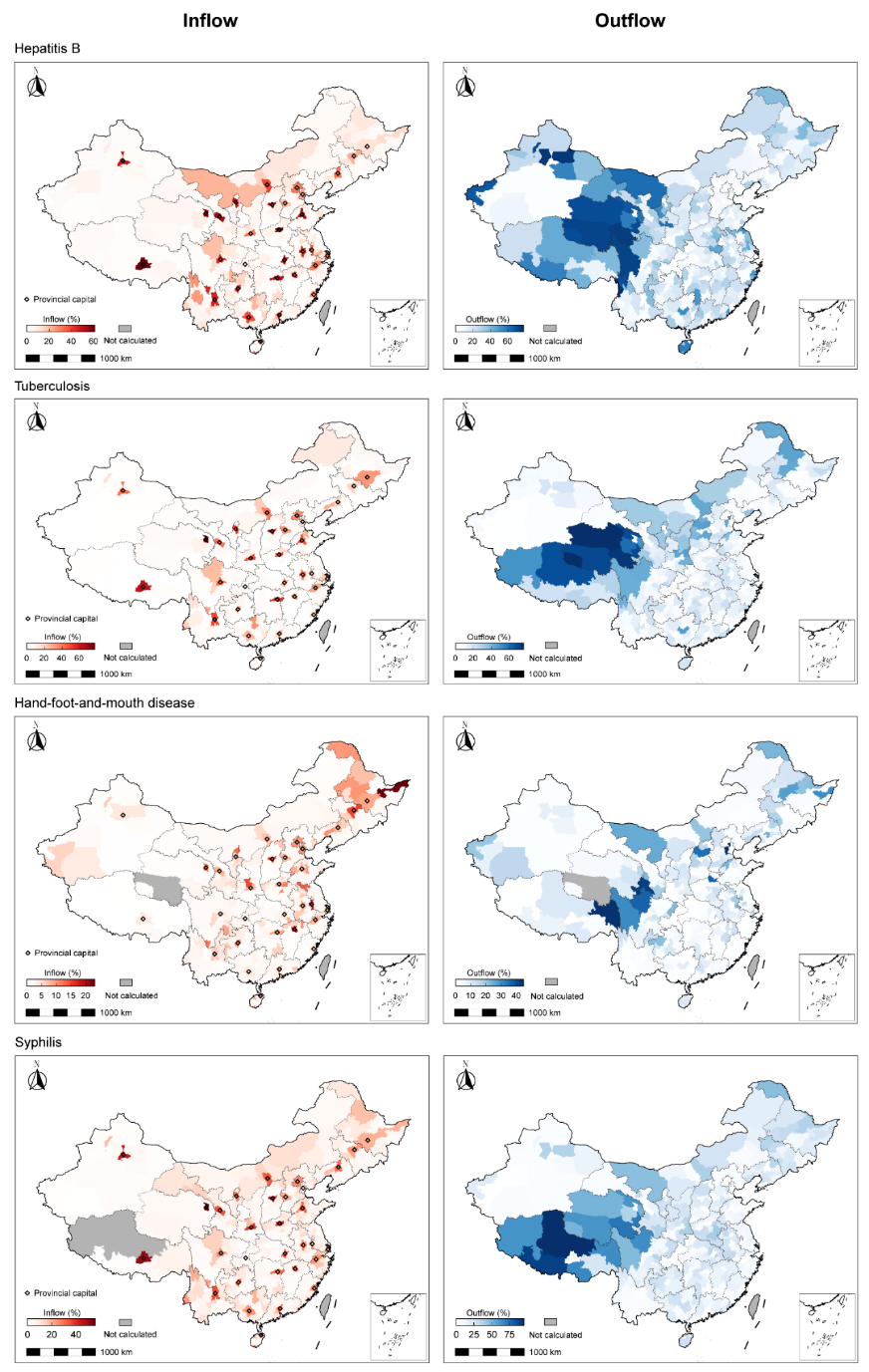


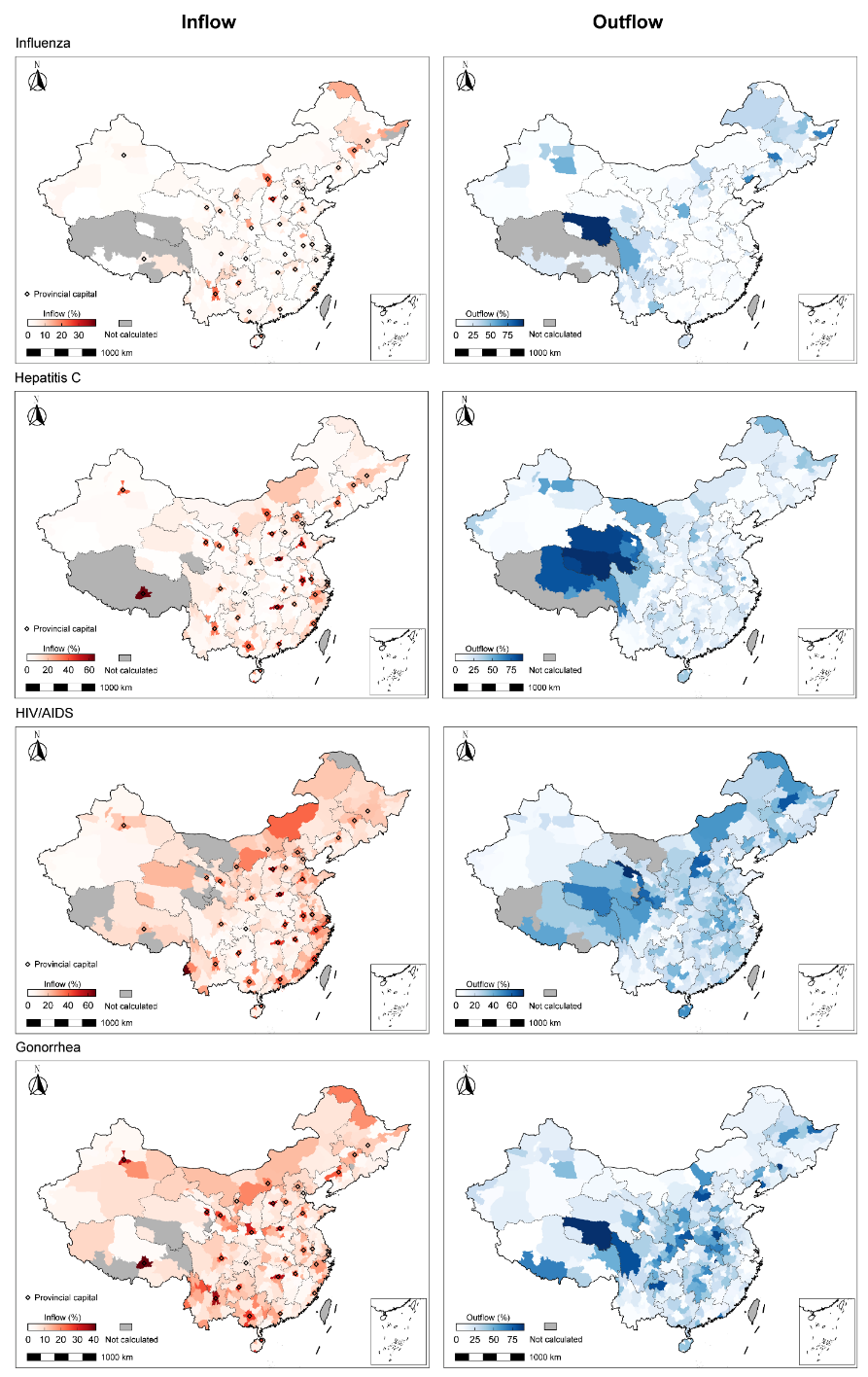

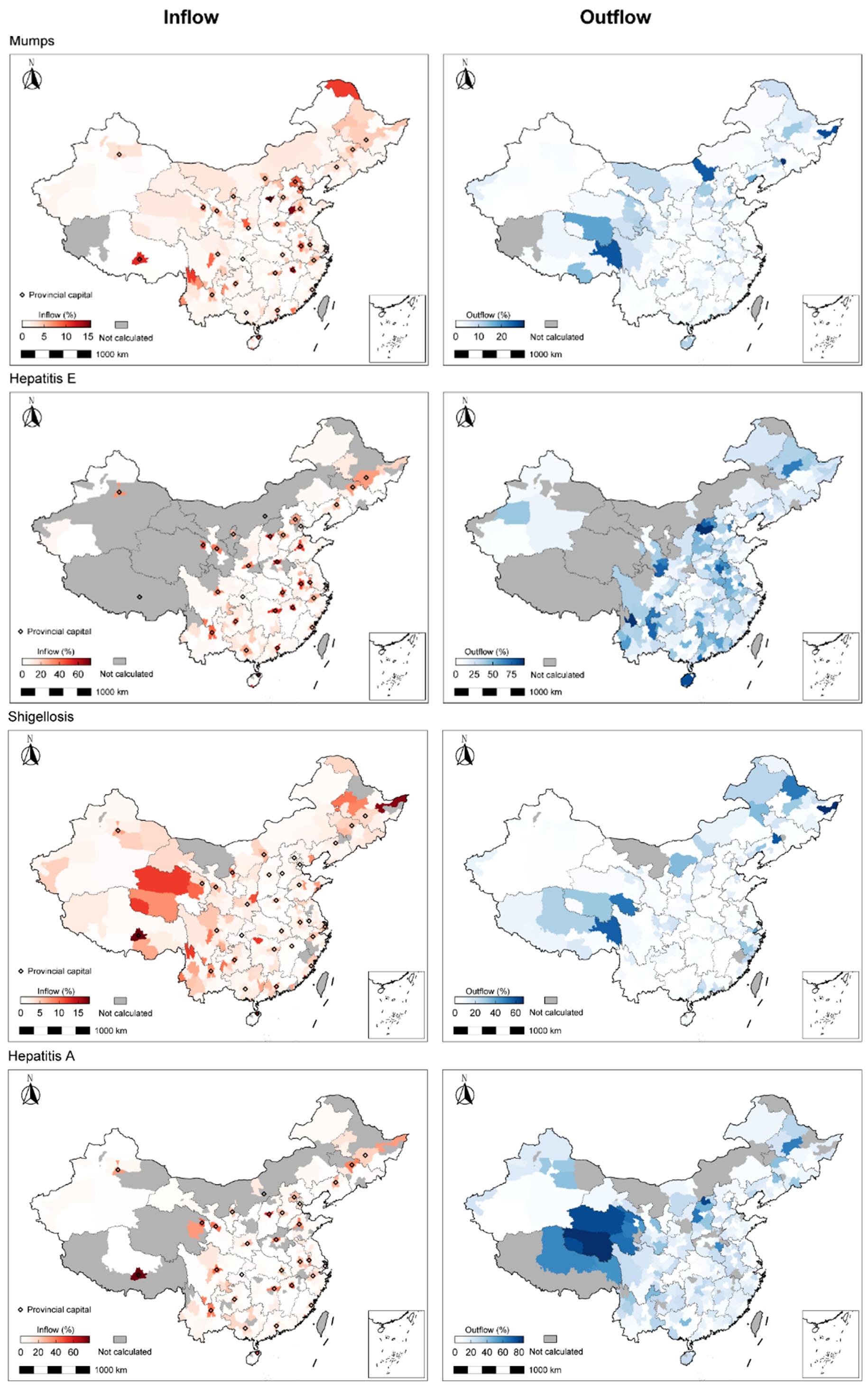


**Figure S9. Distributions of node degrees and node strengths in migration networks of NIDs. The red solid line and the black dashed line indicate the mean and median values of the node degree/strength, respectively.**

**
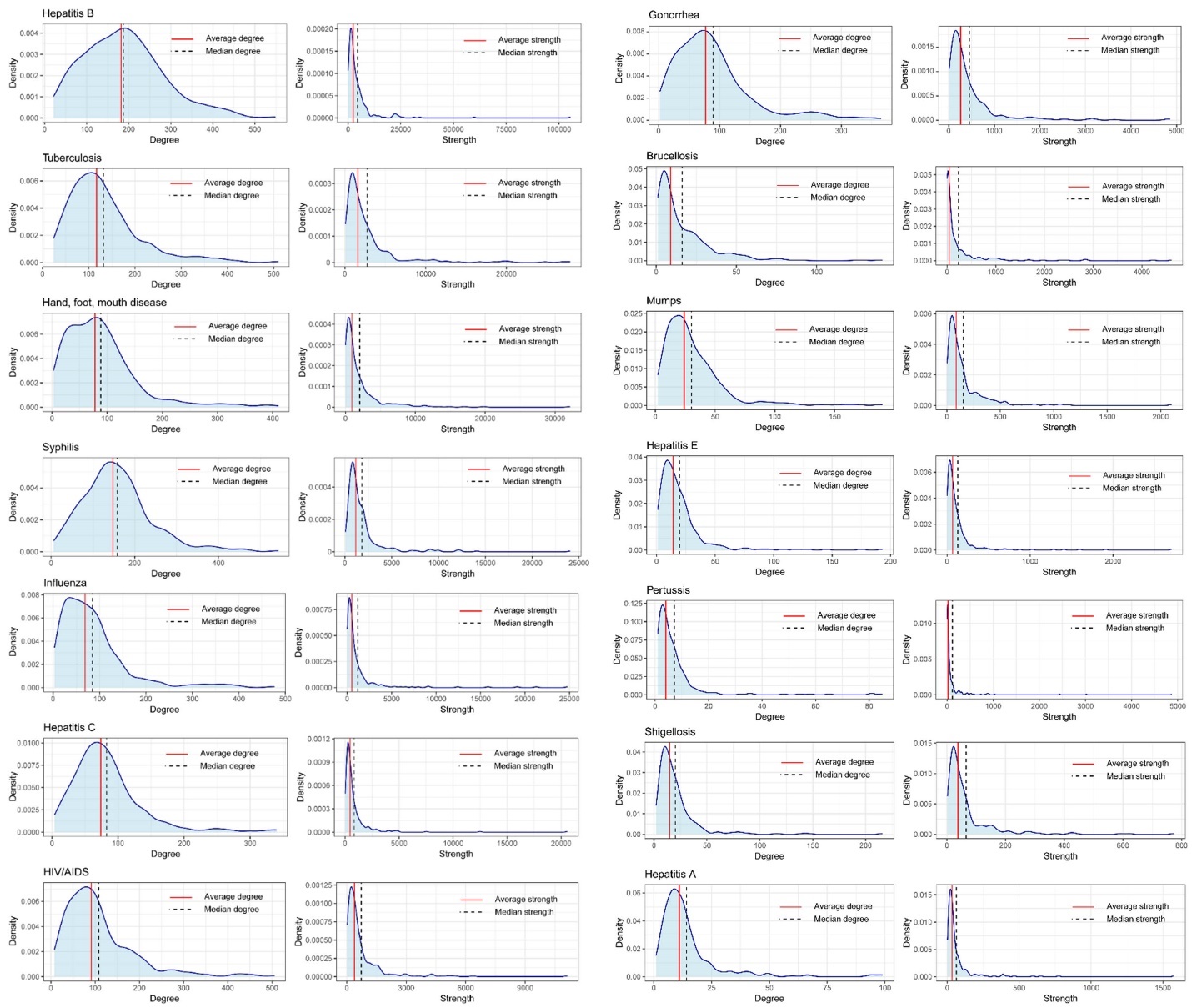
**

**Figure S10. Network backbones of infectious disease migration networks in the short-distance mode. The color of each directed edge changes from dark blue to bright blue to represent the direction of migration from the origin to the destination. The size of each node (city) represents the node strength of the city in the migration network. Each node is colored by the node betweenness in the network, with darker red indicating a larger value of node betweenness.**

**
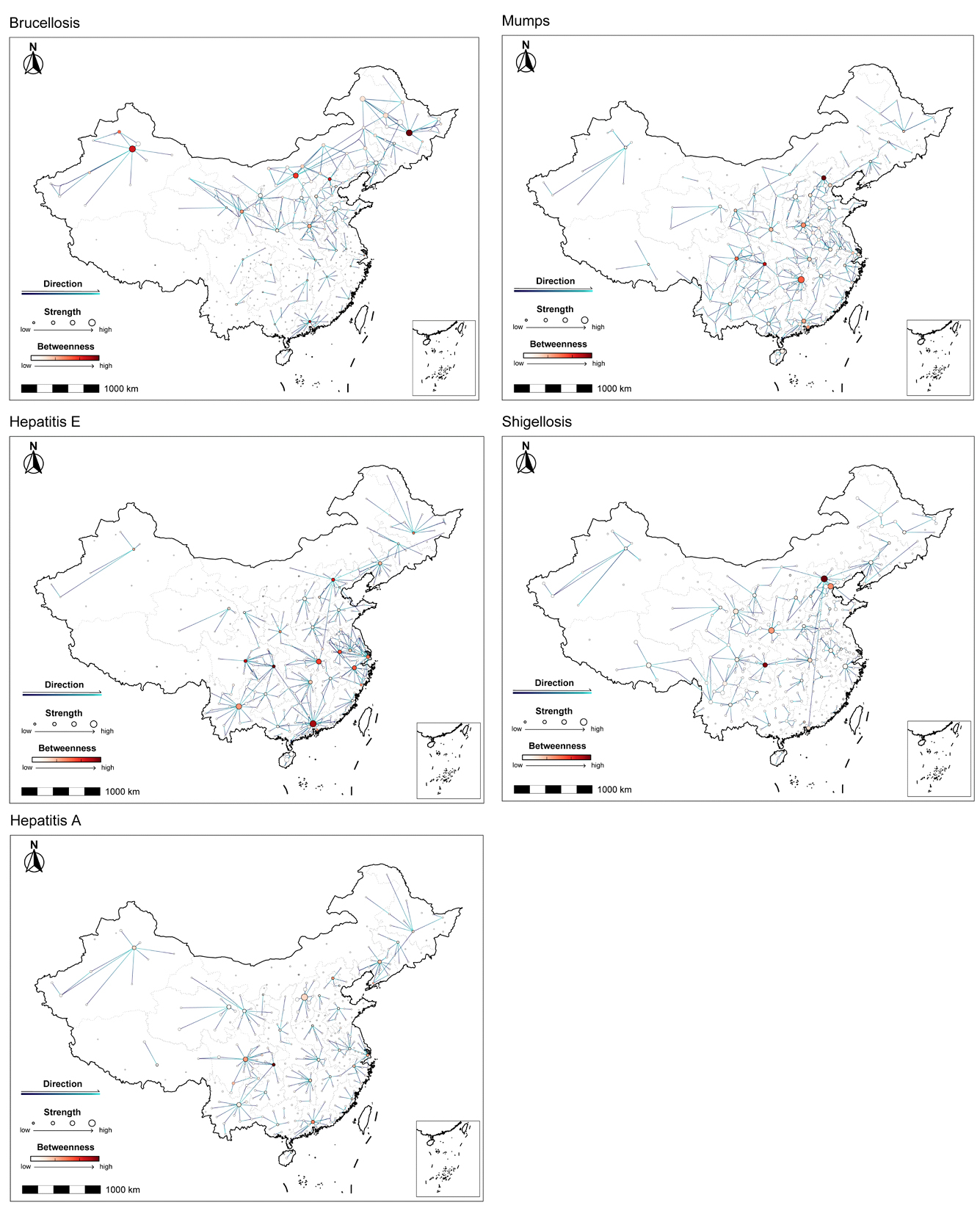
**

**Figure S11. Network backbones of infectious disease migration networks in the long-distance mode. The color of each directed edge changes from dark blue to bright blue to represent the direction of migration from the origin to the destination. The size of each node (city) represents the node strength of the city in the migration network. Each node is colored by the node betweenness in the network, with darker red indicating a larger value of node betweenness.**

**
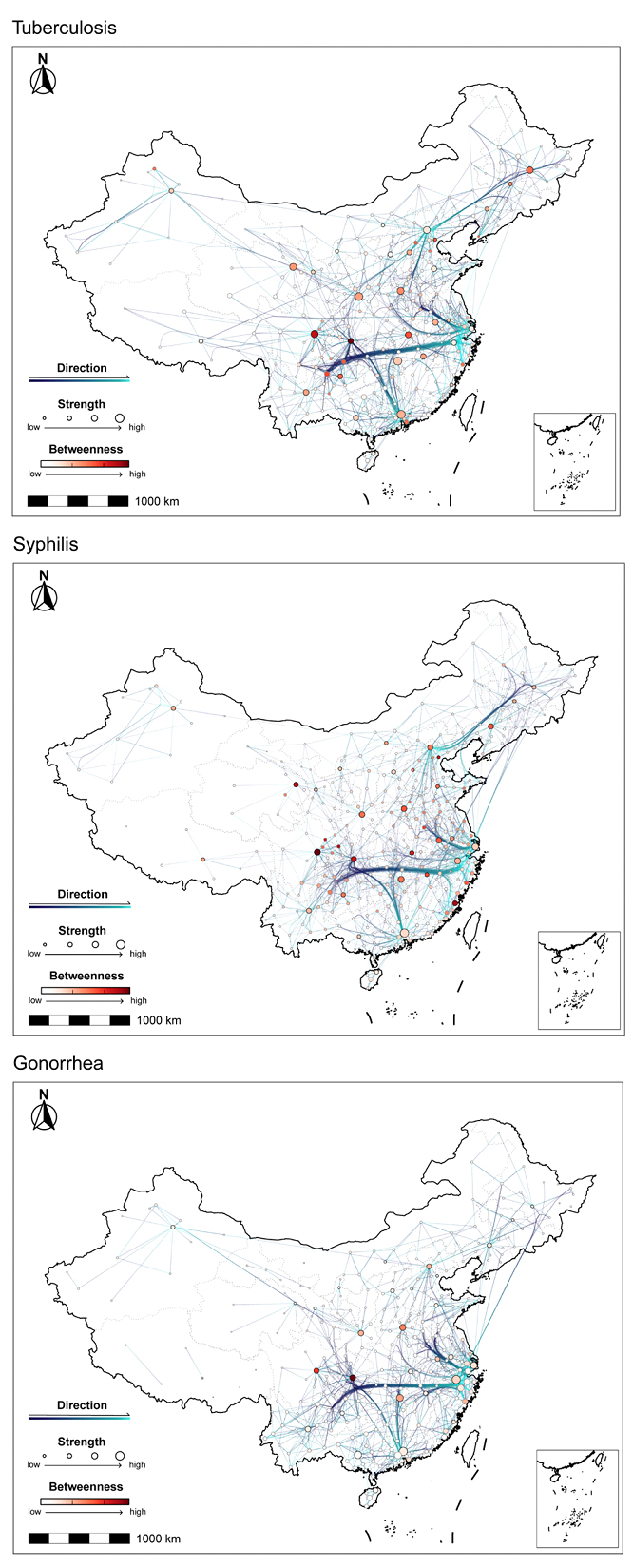
**

**Figure S12. Network backbones of infectious disease migration networks in mixed mode. The color of each directed edge changes from dark blue to bright blue to represent the direction of migration from the origin to the destination. The size of each node (city) represents the node strength of the city in the migration network. Each node is colored by the node betweenness in the network, with darker red indicating a larger value of node betweenness.**

**
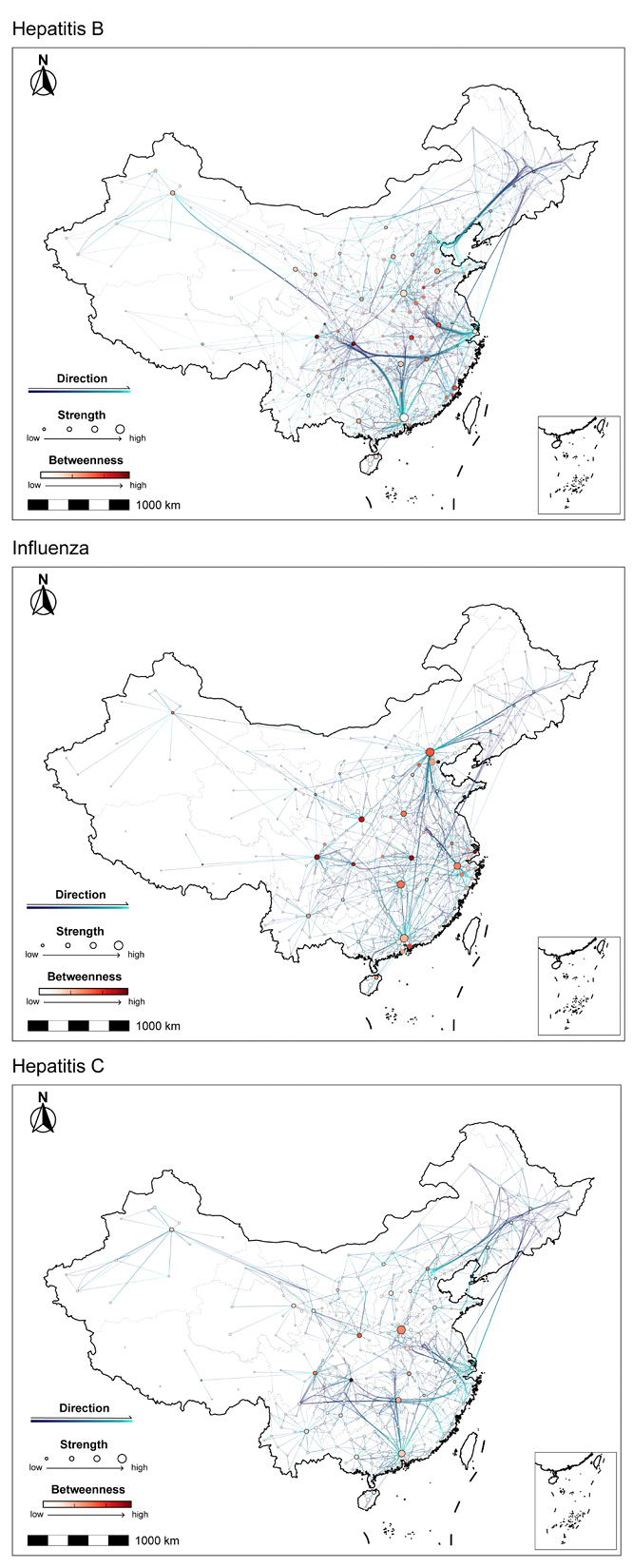
**

**Figure S13. Comparing goodness of fit between the XGBoost model and the gravity model for 14 major notifiable infectious diseases. Data distributions are shown by the histograms in the top and right margins.**


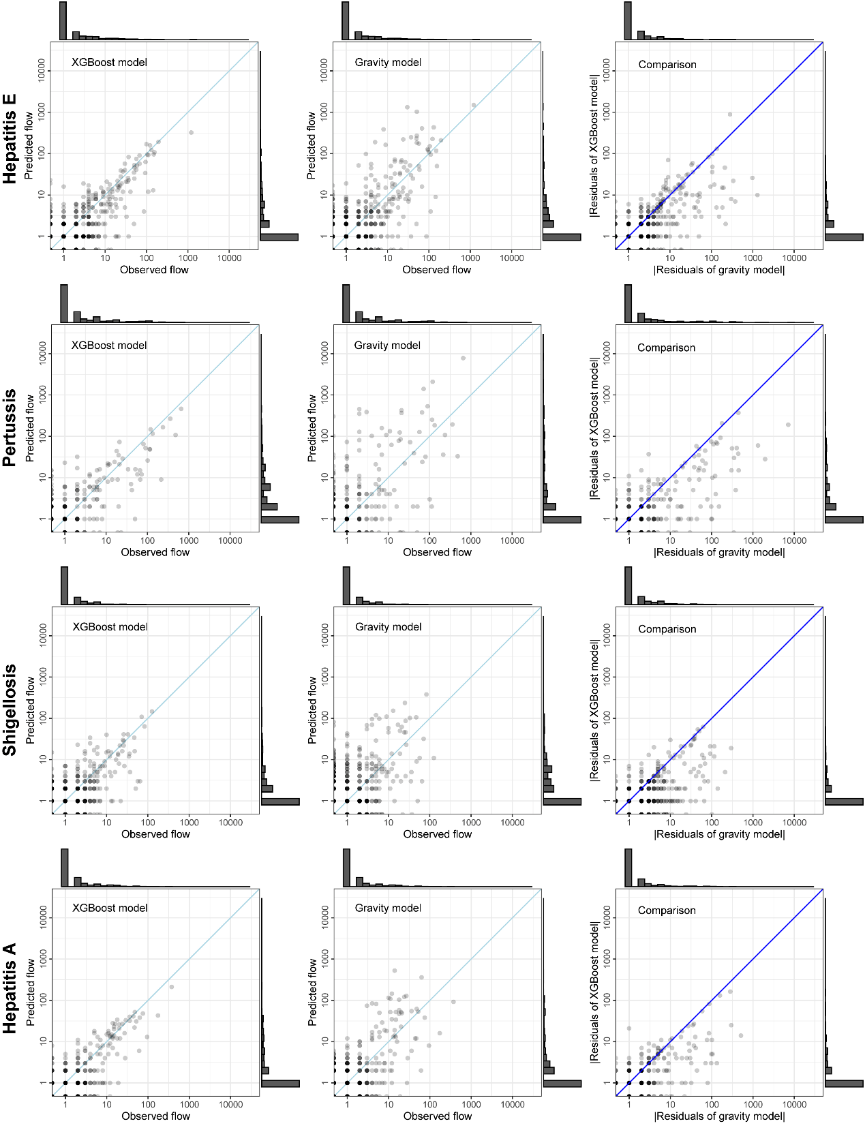

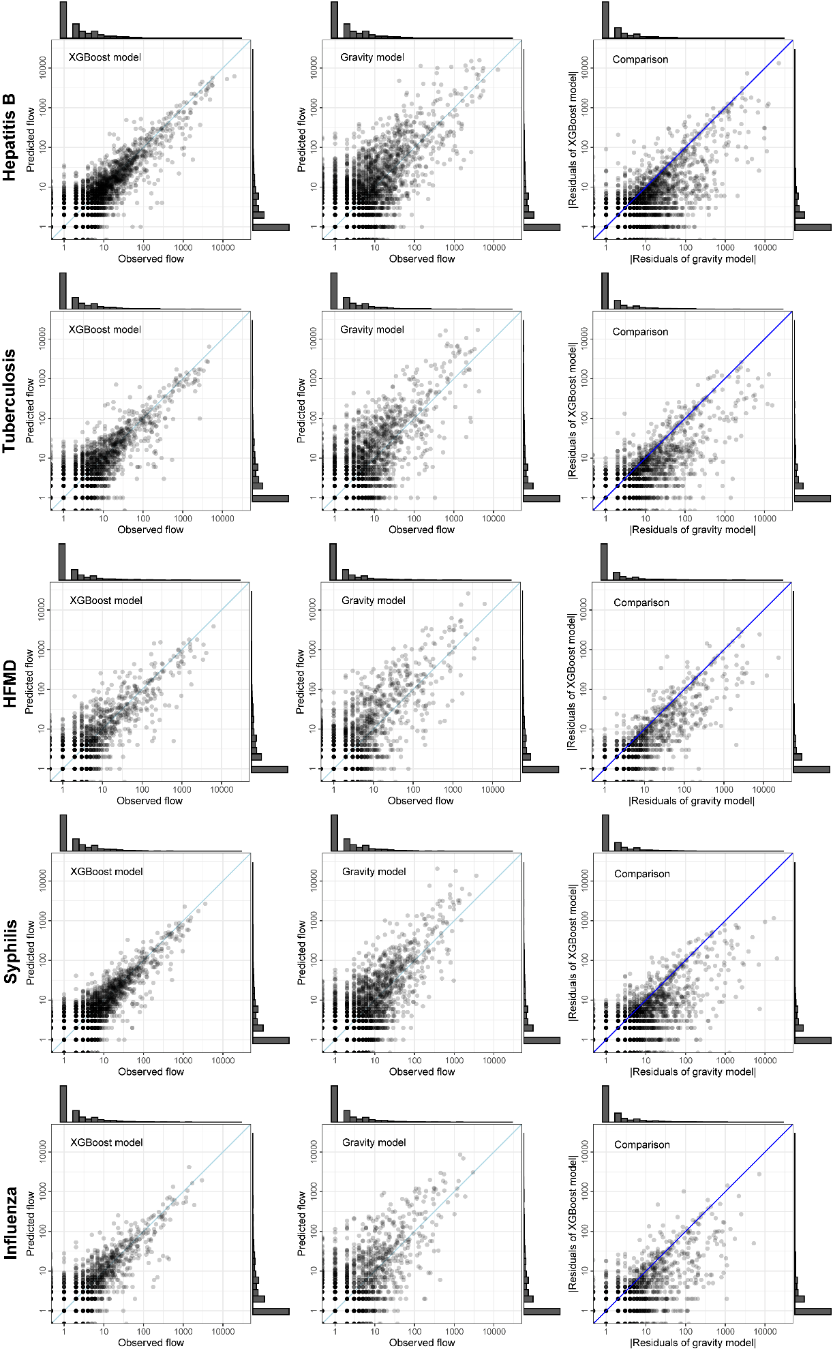

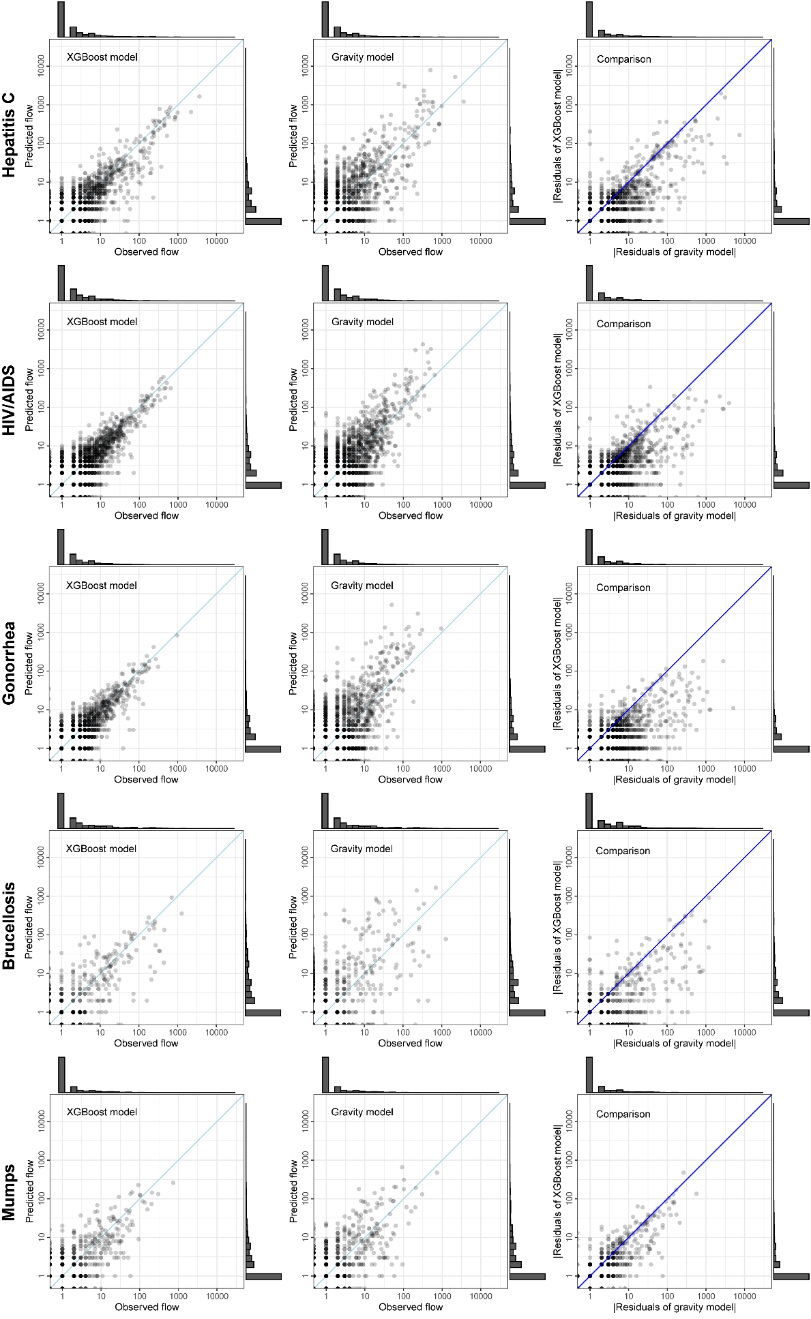


**Figure S14. The impact of human mobility intensity on inter-city migratory case flow as shown by the variation of SHAP values across its range for 14 major notifiable infectious diseases. Each dot represents a record in the test dataset.**

**
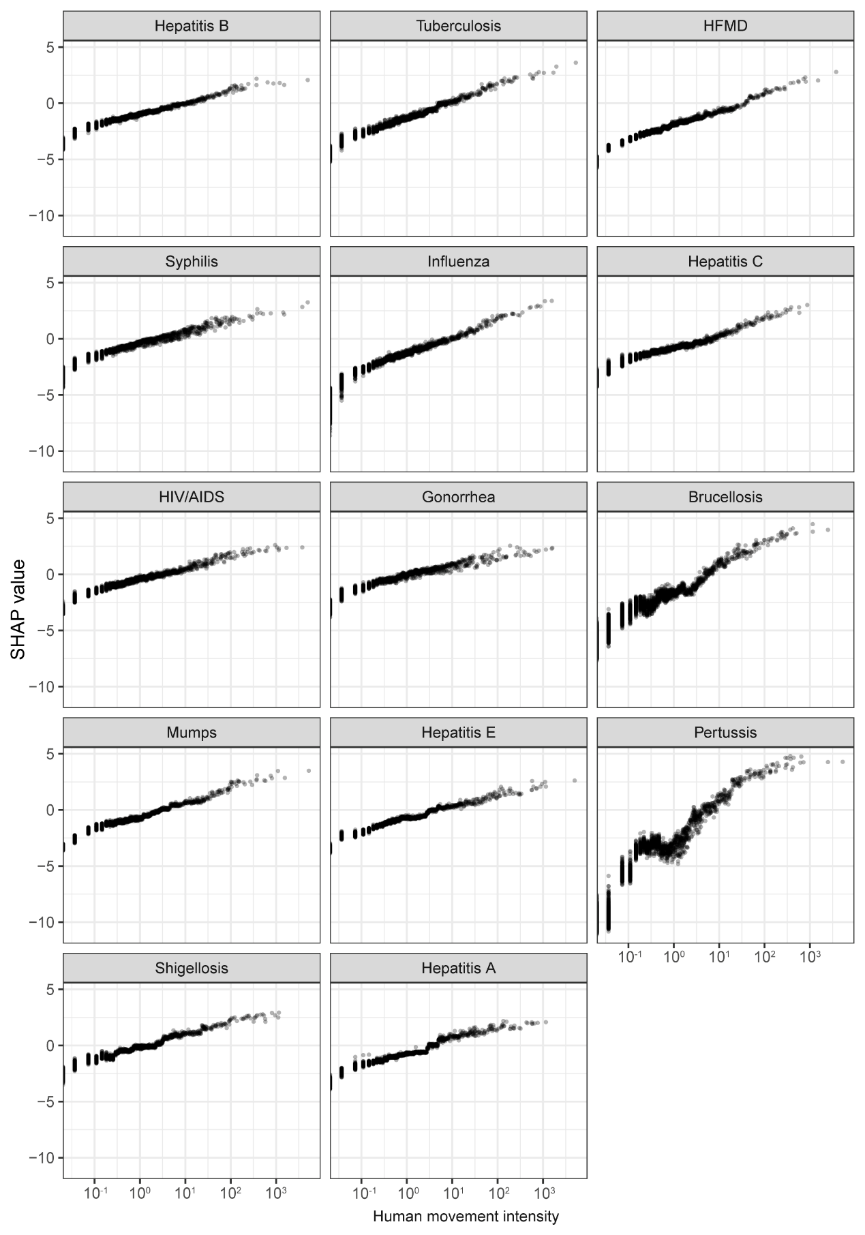
**

**Figure S15. The impact of labor flow intensity on inter-city migratory case flow as shown by the variation of SHAP values across its range for 14 major notifiable infectious diseases. Each dot represents a record in the test dataset.**

**
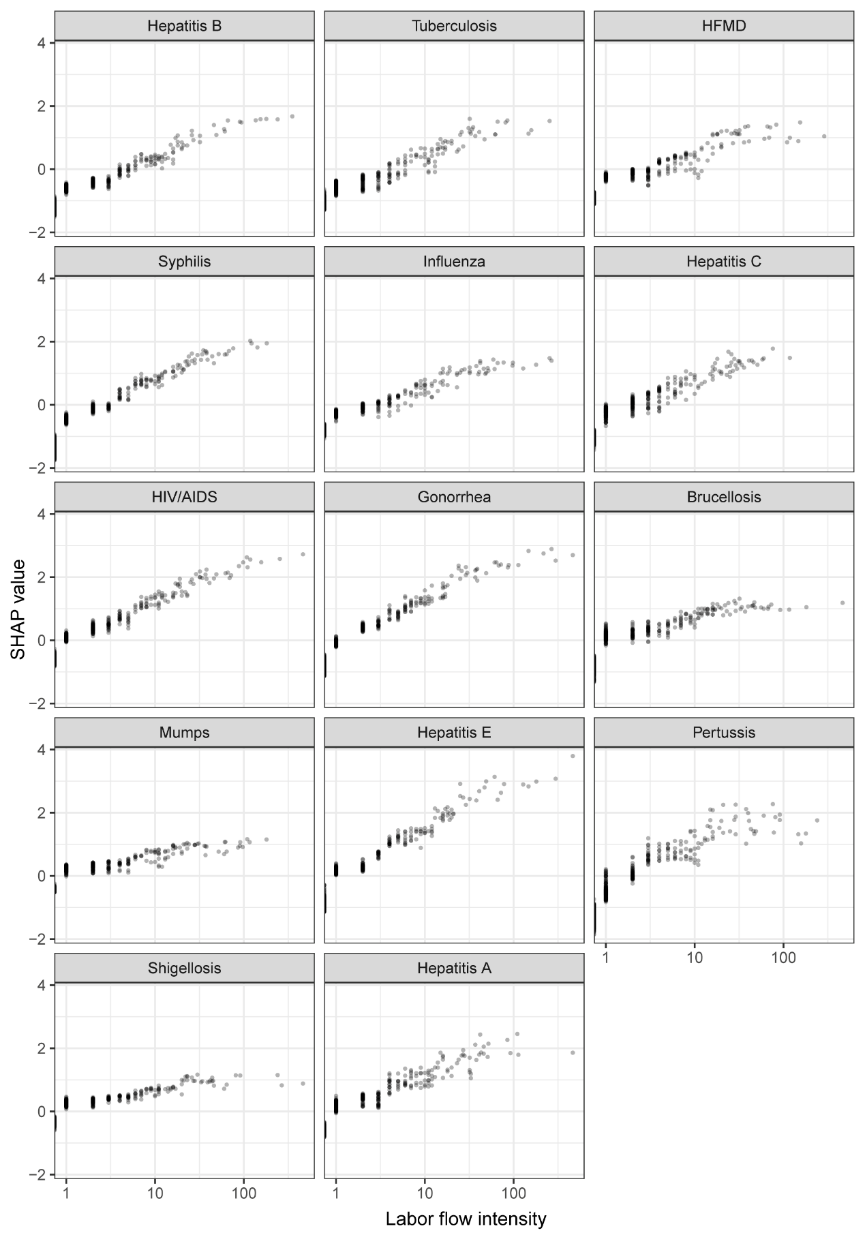
**

**Figure S16. The impact of geographic distance on inter-city migratory case flow as shown by the variation of SHAP values across its range for 14 major notifiable infectious diseases. Each dot represents a record in the test dataset.**

**
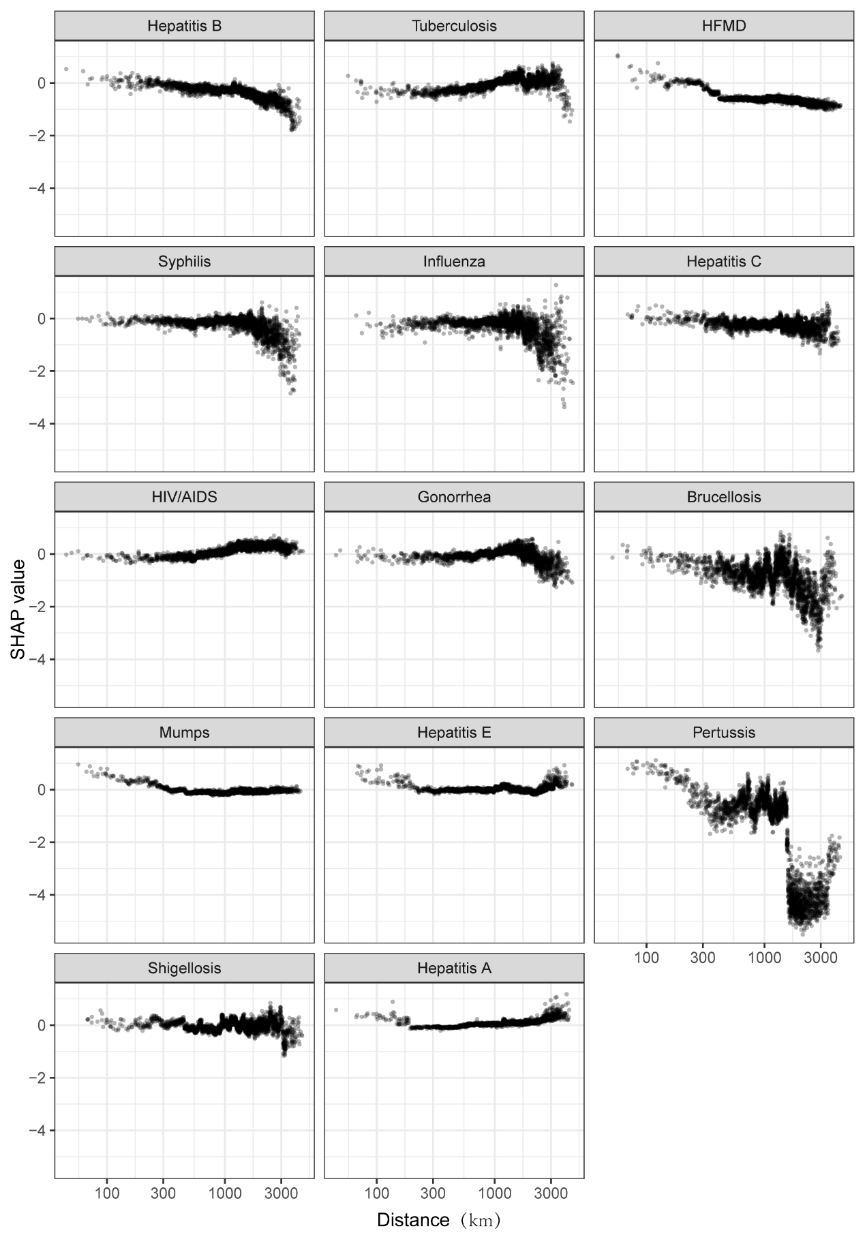
**

**Figure S17. The impact of disease annual incidence at the origin city on inter-city migratory case flow as shown by the variation of SHAP values across its range for 14 major notifiable infectious diseases. Each dot represents a record in the test dataset.**

**
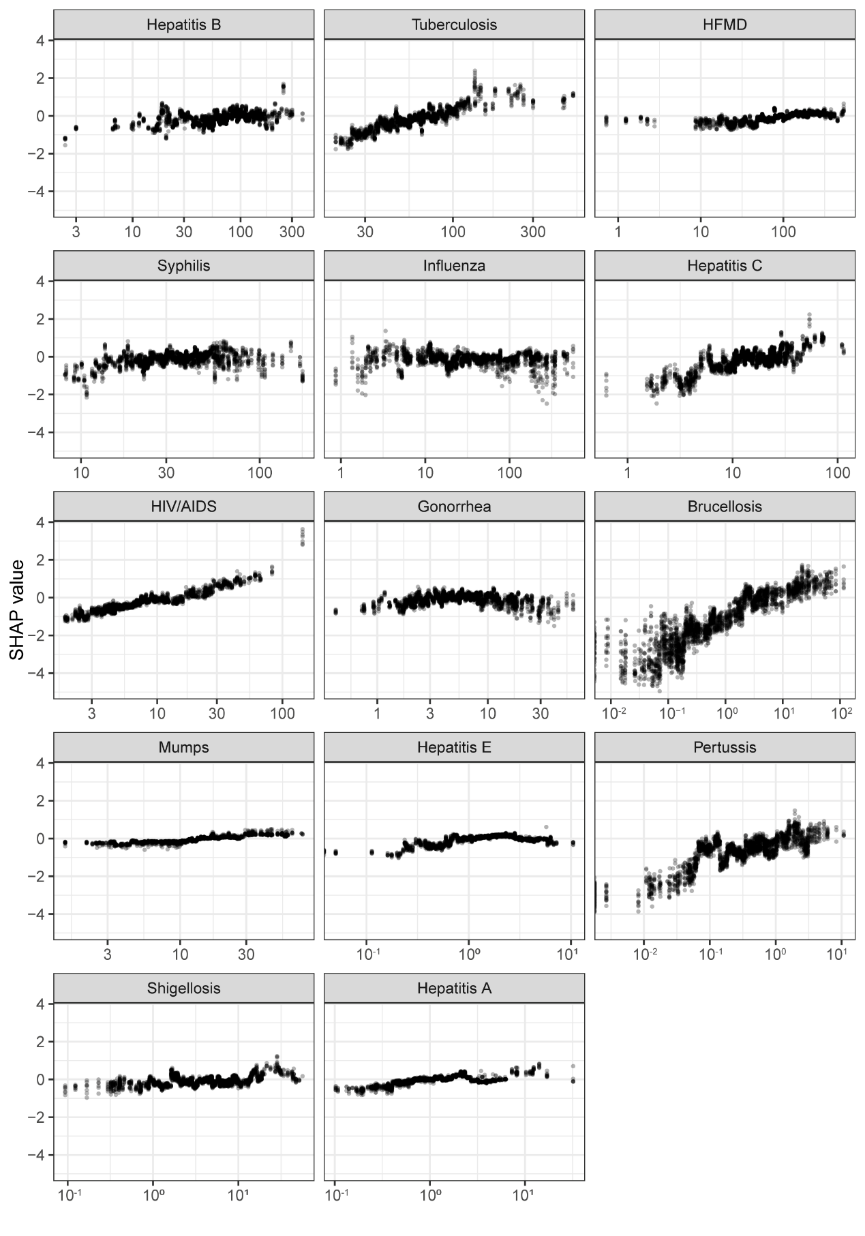
**

**Figure S18. The impact of per capita GRP at the destination city on inter-city migratory case flow as shown by the variation of SHAP values across its range for 14 major notifiable infectious diseases. Each dot represents a record in the test dataset.**

**
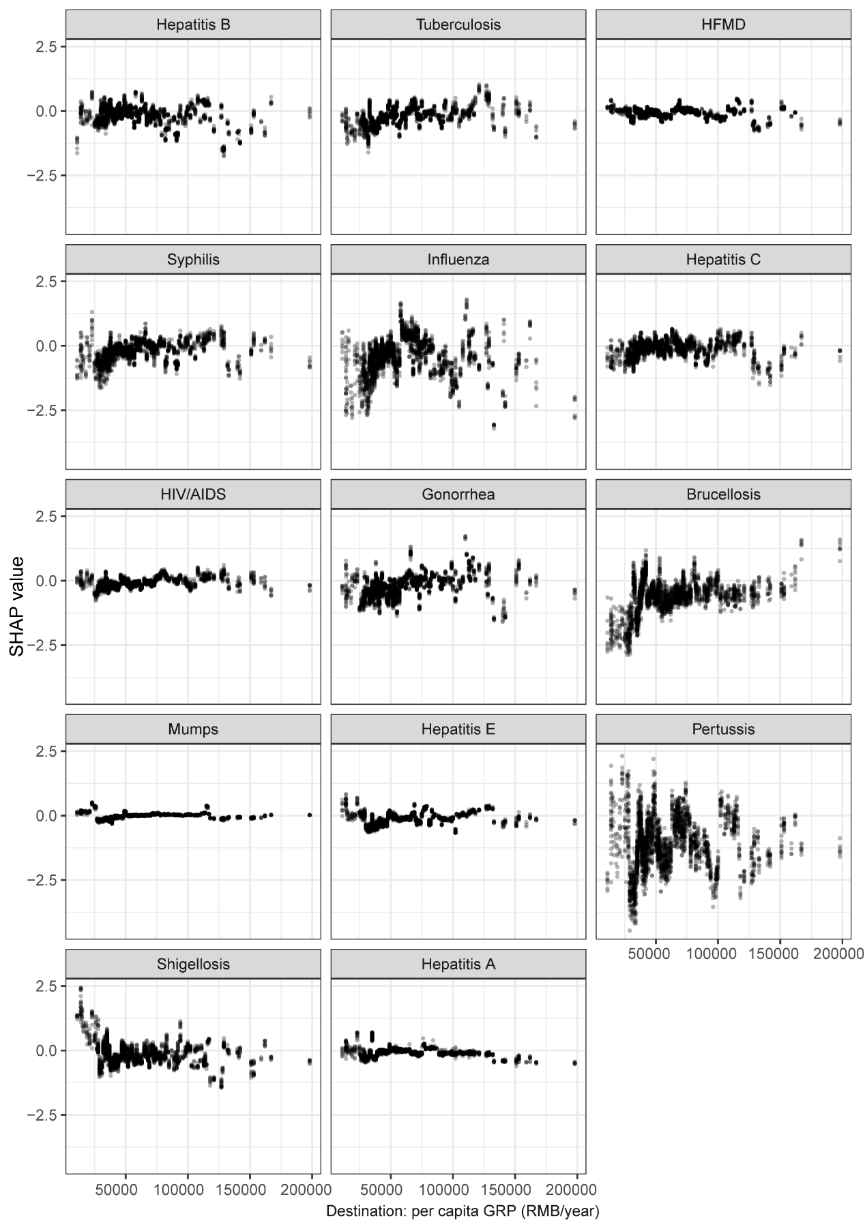
**

**Figure S19. The impact of per capita GRP at the origin city on inter-city migratory case flow as shown by the variation of SHAP values across its range for 14 major notifiable infectious diseases. Each dot represents a record in the test dataset.**

**
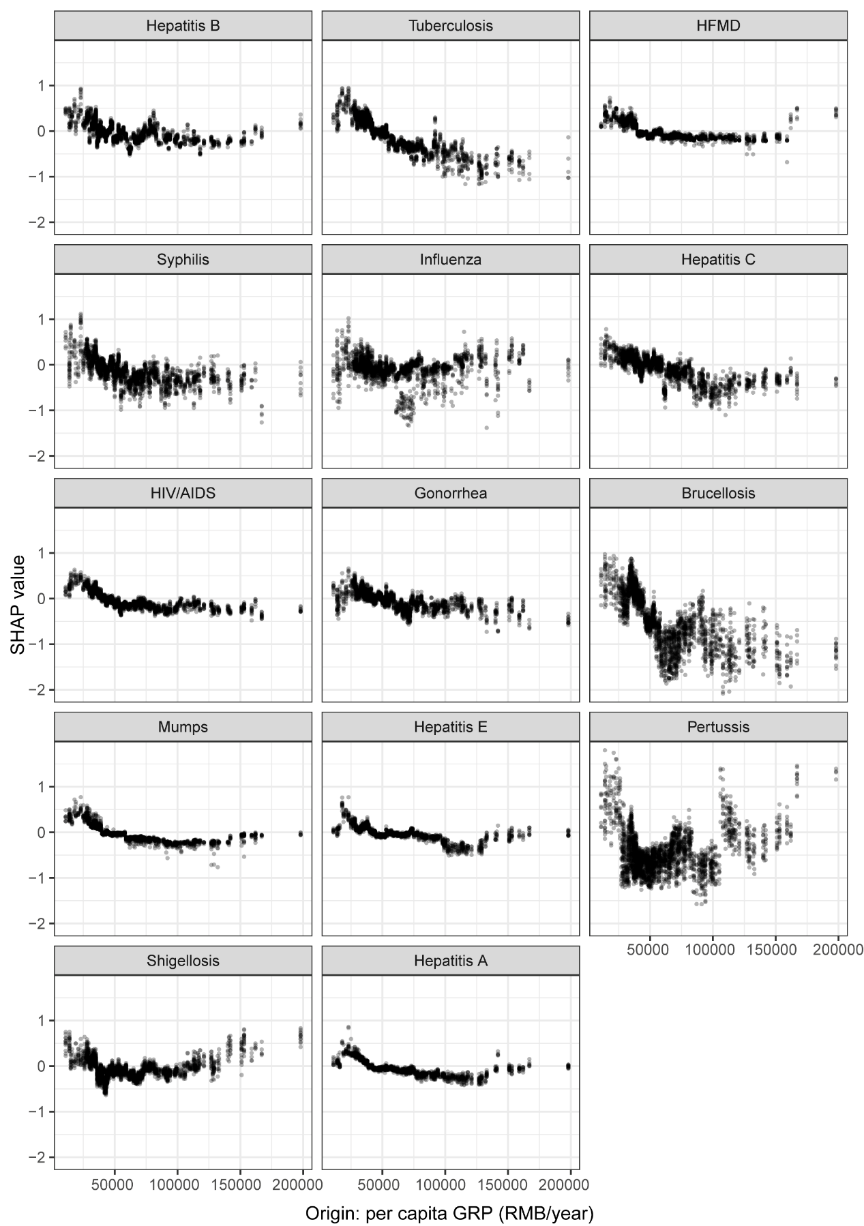
**

**Figure S20. The impact of the proportion (%) of secondary and tertiary industry sectors in local GRP at the destination city on inter-city migratory case flow as shown by the variation of SHAP values across its range for 14 major notifiable infectious diseases. Each dot represents a record in the test dataset.**

**
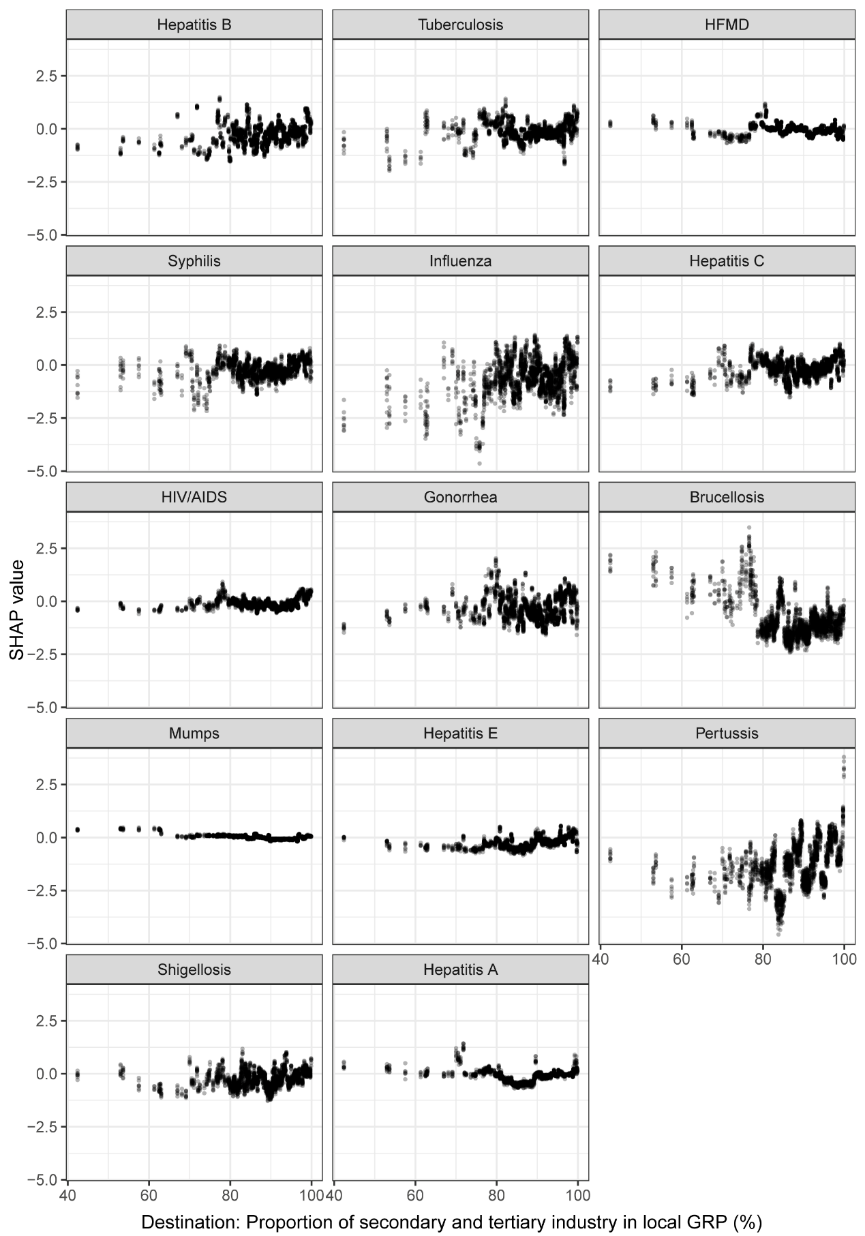
**

**Figure S21. The impact of the proportion (%) of secondary and tertiary industry sectors in local GRP at the origin city on inter-city migratory case flow as shown by the variation of SHAP values across its range for 14 major notifiable infectious diseases. Each dot represents a record in the test dataset.**

**
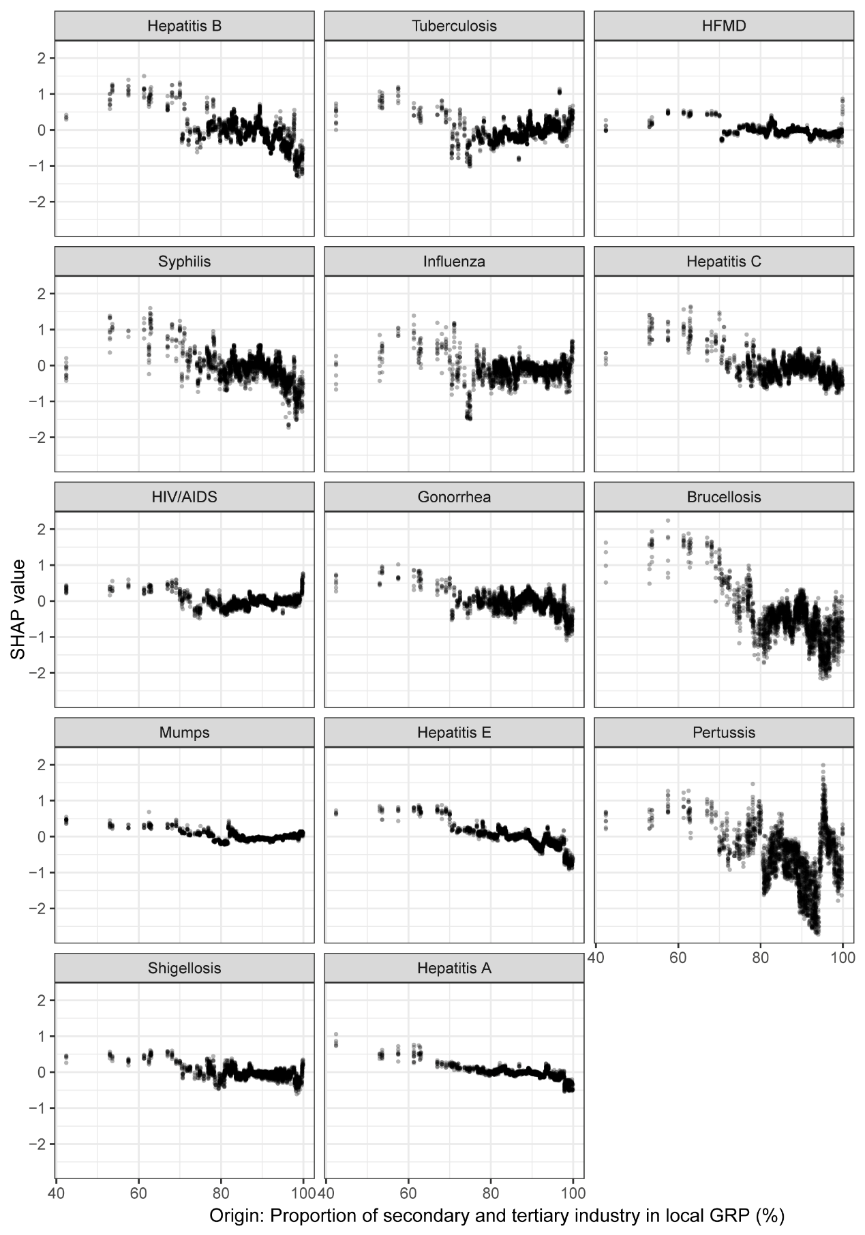
**

**Figure S22. The impact of the four indicators reflecting the administrative level and geographical relationship on inter-city migratory case flow as shown by the variation of SHAP values across its range for 14 major notifiable infectious diseases.**

**
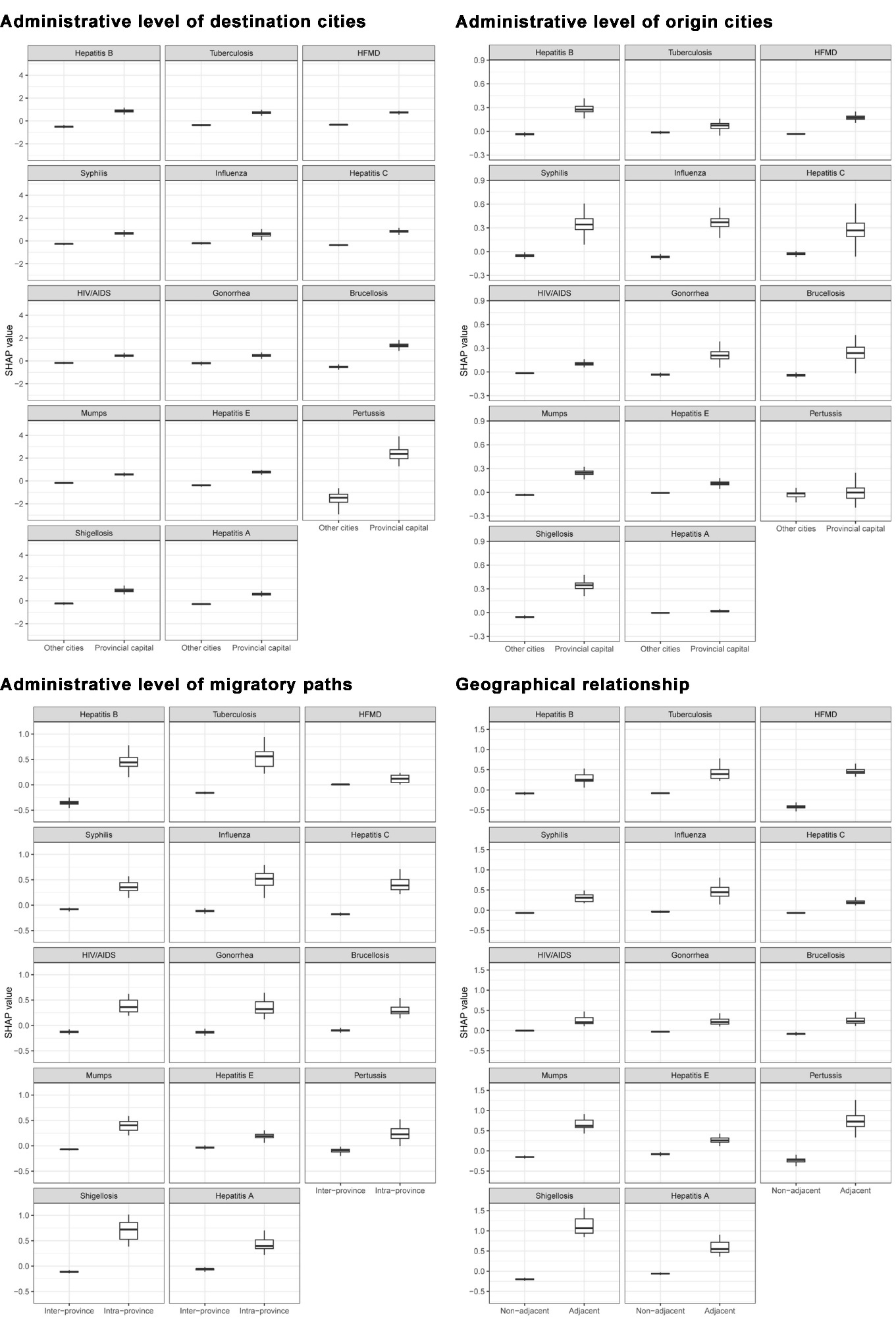
**

**Figure S23. Monthly disease spectrum of migratory cases of notifiable infectious diseases in China during 2016–2020. Pertussis, hepatitis E, Shigellosis and hepatitis A are combined in the “other” category.**

**
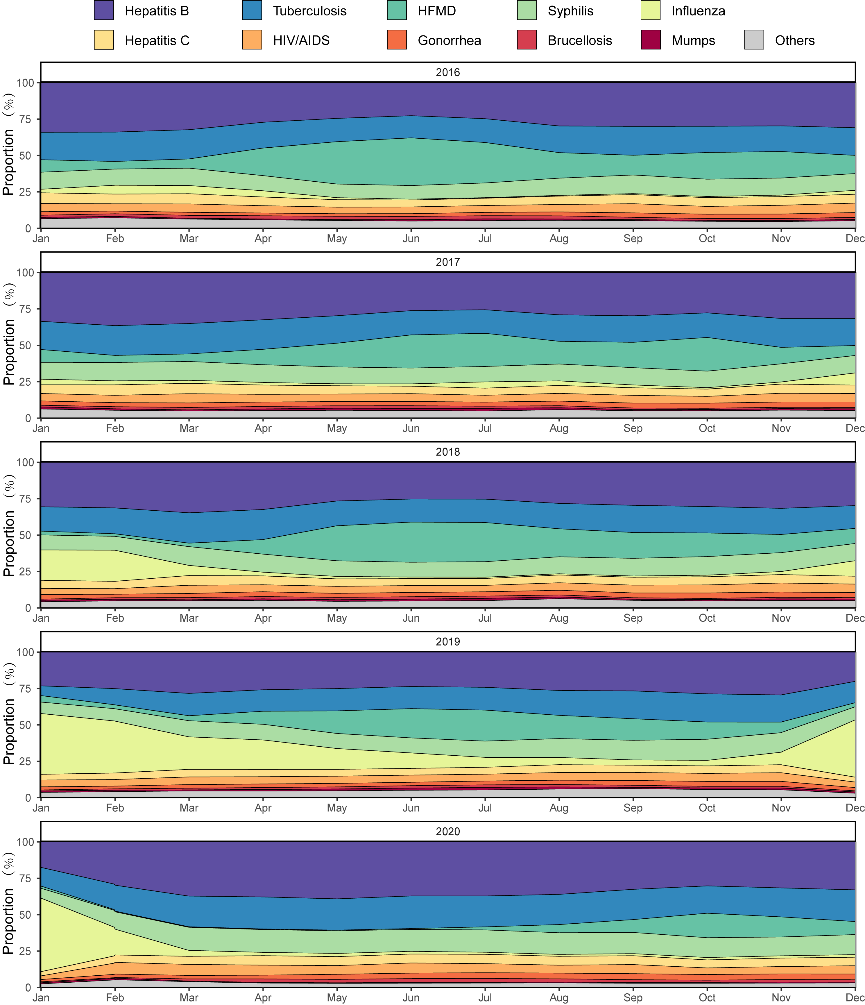
**
